# New Insights Into the Plastome Evolution of the Millettioid/Phaseoloid Clade (Papilionoideae, Leguminosae)

**DOI:** 10.3389/fpls.2020.00151

**Published:** 2020-03-10

**Authors:** Oyetola Oyebanji, Rong Zhang, Si-Yun Chen, Ting-Shuang Yi

**Affiliations:** ^1^ Germplasm Bank of Wild Species, Kunming Institute of Botany, Chinese Academy of Sciences, Kunming, China; ^2^ Kunming College of Life Science, University of Chinese Academy of Sciences, Beijing, China

**Keywords:** evolutionary relationships, inversion, IR expansion/contraction, Leguminosae, Plastome, the Millettioid/Phaseoloid clade

## Abstract

The Millettioid/Phaseoloid (MP) clade from the subfamily Papilionoideae (Leguminosae) consists of six tribes and ca. 3,000 species. Previous studies have revealed some plastome structural variations (PSVs) within this clade. However, many deep evolutionary relationships within the clade remain unresolved. Due to limited taxon sampling and few genetic markers in previous studies, our understanding of the evolutionary history of this clade is limited. To address this issue, we sampled 43 plastomes (35 newly sequenced) representing all the six tribes of the MP clade to examine genomic structural variations and phylogenetic relationships. Plastomes of the species from the MP clade were typically quadripartite (size ranged from 140,029 to 160,040 bp) and contained 109–111 unique genes. We revealed four independent gene losses (*ndhF*, *psbI*, *rps16*, and *trnS-GCU*), multiple IR-SC boundary shifts, and six inversions in the tribes Desmodieae, Millettieae, and Phaseoleae. Plastomes of the species from the MP clade have experienced significant variations which provide valuable information on the evolution of the clade. Plastid phylogenomic analyses using Maximum Likelihood and Bayesian methods yielded a well-resolved phylogeny at the tribal and generic levels within the MP clade. This result indicates that plastome data is useful and reliable data for resolving the evolutionary relationships of the MP clade. This study provides new insights into the phylogenetic relationships and PSVs within this clade.

## Introduction

The plastid genome (plastome) usually shows a quadripartite structure including a large-single-copy (LSC, 60–90 kb) region, a small-single-copy (SSC, 7–27 kb) region, and a pair of inverted repeats (IR, 20–76 kb) (Raubeson and Jansen, 2005; Zhang et al., 2018). As a circular genome of about 108–218 kb in size, the plastome contains ca. 90–130 unique genes including 80–90 protein-coding genes (PCGs), 30–31 transfer RNA (tRNAs) and 4 ribosomal RNAs (rRNAs) (Bock, 2007; Wu et al., 2009; Green, 2011). The plastome structure of autotrophic plants is usually conserved (Kim and Jansen, 2005; Lin et al., 2015). However, significant structural variations including IR loss, IR contraction/expansion, inversion, pseudogenization, gene duplication, and gene loss have been reported in some gymnosperms (Wu and Chaw, 2016) and angiosperm families such as Campanulaceae (Cosner et al., 1997; Haberle et al., 2008), Geraniaceae (Chumley et al., 2006; Guisinger et al., 2011; Weng et al., 2014), Oleaceae (Lee et al., 2007), Petrosaviaceae (Logacheva et al., 2014), and Leguminosae (Lavin et al., 1990; Wojciechowski, 2003; Luo et al., 2016; Choi and Choi, 2017; Wang et al., 2017).

Some species of Leguminosae, especially those of the subfamily Papilionoideae, have acquired significant plastome structural variations (PSVs) during their evolution. These PSVs includes loss of IR (e.g., Lavin et al., 1990; Doyle et al., 1996), gene or plastome segment inversion (Choi and Choi, 2017), IR expansion, and/or contraction (Choi and Choi, 2017), and gene loss (Jansen et al., 2007; Sabir et al., 2014; Asaf et al., 2017). Most members of papilionoids, with the exception of a few early diverging lineages, share a 50-kb inversion in the LSC (Doyle et al., 1996). Previous studies have reported multiple inversions of 23, 24, or 36-kb in the Genistoid clade (Martin et al., 2014; Choi and Choi, 2017; Feng et al., 2017; Keller et al., 2017), a 39-kb inversion in *Robinia* (Schwarz et al., 2015), and a large 78-kb inversion in the subtribe Phaseolinae of tribe Phaseoleae (Bruneau et al., 1990). However, only a few studies have examined PSV in the Millettioid/Phaseoloid clade (hereafter referred as the MP clade), one of the most species-rich clades within subfamily Papilionoideae.

The MP clade consists of more than 3,000 extant species with a global distribution (Schrire, 2005a; Schrire, 2005b; Schrire, 2005c and Schrire, 2005d; Schrire et al., 2009). Many species of this clade are economically important (Simpson and Ogorzaly, 2001; Baker, 2004), as edible seeds [*Glycine max* (L.) Merr (soybean), *Cajanus cajan* (L.) Millsp. (pigeon pea), *Phaseolus vulgaris* L. (kidney bean), *Vigna unguiculata* (L.) Walp. (cowpea), and *Pachyrhizus erosus* (L.) Urb (Mexican yam bean)], medicines [*Abrus precatorius* L. (crab eye)], ornamentals [*Canavalia gladiata* (Jacq.) DC. (Sword beans pea) and *Millettia pinnata* (L.) Panigrahi (Indian beech)], forages [*Pueraria phaseoloides* Benth (tropical kudzu)], and woods [*M*. *laurentii* De Wild. (African rosewood)].

Some previous studies based on nuclear ribosomal ITS (Hu et al., 2002) and a few plastid loci (Hu et al., 2000; Kajita et al., 2001; Pennington et al., 2001; Wojciechowski et al., 2004; Cardoso et al., 2013; LPWG, 2017) have made progress in clarifying evolutionary relationships of the MP clade. However, some deep relationships, particularly at tribe level, have not been fully resolved, perhaps due to limited phylogenetic signals in these gene loci. Whole plastome sequences have been successfully applied to resolve plant evolutionary relationships (Jansen et al., 2007), and therefore they might be of use for clarifying unresolved relationships in the MP clade. A few recent studies using limited samples have detected multiple types of PSV in this clade, such as a 78-kb inversion in *Vigna radiata* (L.) R. Wilczek and *P. vulgaris*, a 36-kb inversion in *Lupinus luteus* L. (Martin et al., 2014), the loss of *rps16* gene in *Cajanus* Adans. (Guo et al., 2007; Schwarz et al., 2015), the loss of *rpl2* and *clpP* introns (Kaila et al., 2016), and IR contraction/expansion in *G. max* (Saski et al., 2005; Kim et al., 2015). Investigation of the plastome of more taxa of this clade is essential for a better understanding of PSVs across this clade. In this study, we analyzed plastomes of 43 species (35 newly sequenced) representing all the six tribes of the MP clade. We investigated plastome structural diversification, and conducted phylogenetic reconstruction of the clade using plastome sequences. Deep phylogenetic relationships of the MP clade were investigated using the coding genes (CDs), noncoding regions (NCDs) and complete plastomes (CP). Our study provides important new insights into both phylogenetic relationships and PSVs within the MP clade.

## Materials and Methods

### Taxon Sampling, DNA Extraction, and Genome Sequencing

For this study, we used a total of plastomes of 43 species from the MP clade including one plastome from NCBI, seven plastomes from Zhang et al. (2020)’s phylogenetic study of the whole family, and newly sequenced plastomes of 35 species from 35 genera (**Supplementary Table S1**). These species were selected based on the availability of tissues for sampling and their representation of previously recognized tribes in the clade (LPWG, 2013). Total genomic DNA (gDNA) was extracted from either fresh or silica-gel dried leaves using the modified CTAB method (Doyle and Doyle, 1987). The genome skimming method was used to obtain the plastome data (Zeng et al., 2018). The gDNA was fragmented and libraries size were selected for 350 bp inserts. Sequencing with 2 × 150-bp paired-end (PE) reads was performed on the Illumina Hiseq 2500/X-Ten at the Novogene (Tianjin, China) or Illumina Hiseq 2000/2500/4000/X-Ten at the Beijing Genomics Institute (BGI) in Shenzhen, China.

### Plastome Assembly and Annotation

The clean-up and quality control checks of the raw reads were performed using the Next Generation Sequencing (NGS) QC Tool Kit with default settings (Patel and Jain, 2012). Then, we assembled contigs from the PE reads *via de novo* assembly using GetOrganelle (Jin et al., 2019) with K-mer values 21, 45, 65, 85, 105, and 127 calling SPAdes version 3.10 (Bankevich et al., 2012), using a reference genome from subfamily Papilionoideae (*Arachis hypogaea* L., NC_026676). Bandage v.0.80 (Wick et al., 2015) was used to visualize and filter the assembled contigs to generate a complete circular plastome. For incomplete plastomes, we filled the gaps between the contigs with consensus sequences of raw reads that were initially mapped to the reference plastome in order to obtain the complete plastome. The number of the mapped PE reads and the coverage depth were determined by mapping the paired reads against the plastome using Bowtie2 (Langmead and Salzberg, 2012) incorporated in Geneious v. 8.1.4 (Kearse et al., 2012).

The locations of the single copy (SC) and IR boundaries in the newly sequenced plastomes were determined using the same methods as Qu et al. (2019). The ‘find repeat’ function in Geneious was used to flank the IR regions. Then, the paired reads were remapped to the assembled plastomes to validate the SC/IR regions using Bowtie2. Finally, we visualized the read stacks of the newly assembled plastomes and compared the marked SC/IR boundaries in Geneious. The new plastomes were annotated using Dual Organellar Genome Annotator (DOGMA) web-interface (Wyman et al., 2004). We manually checked the consistency of start/stop codons and intron/exon boundaries in Geneious. The ‘Find ORFs’ function in Geneious was used to re-confirm the PCGs annotations, while tRNAscan-SE web service was applied to determine the tRNA genes (Schattner et al., 2005). The OrganellarGenomeDRAW [web server, (Lohse et al., 2013)] was used to draw the physical genomic map (**Supplementary Figure S1**). Finally, the complete newly assembled plastomes (35 in the MP clade and four outgroup species) were deposited in GenBank (**Supplementary Table S1**).

### Plastome Structural Analysis

To investigate the patterns of genomic evolution, we analyzed and compared the structural characteristics of the 43 annotated plastomes. We examined structural characteristics such as plastome size (bp), LSC length (bp), SSC length (bp), IR length (bp), GC content (%), and gene distributions of all studied plastomes (Li et al., 2013; **Supplementary Table S2**; **Table 1**). For the contraction and expansion analysis, we compared the newly sequenced plastomes of the species from the MP clade with the *A*. *hypogaea* plastome. Afterward, we examined the variation of the genes located at the plastome termini and the boundary shifts (IR-SC) in the four junctions (J_LB_ *–*LSC/IR_B_, J_SB_ *–*IR_B_/SSC, J_SA_ *–*SSC/IR_A_, and J_LA_ *–*IR_A_/LSC) (**Supplementary Figure S2**). To confirm inversions, we aligned the 43 plastomes of species from the MP clade with the *A. hypogaea* plastome using the progressiveMauve algorithm (Wang et al., 2017). We used default settings to automatically calculate the seed weight (15), and calculated Locally Collinear Blocks (LCBs) with the minimum LCB score of 30,000 (Darling et al., 2004). The detected inversions were illustrated in **Figure 3** and **Supplementary Figure S3**.

**Table 1:** Plastome content and their functions in the MP clade.

Function	Gene group	Gene
**Biosynthesis of fatty acids**	Acetyl-CoA carboxylase	*accD*
**Genetic apparatus**	Large subunit of ribosomal protein	***rpl2*** ^a,b^, *rpl14* ^c^, *rpl16* ^c,e,f^, ***rpl23***, *rpl32*, *rpl33*, ***rpl36*** ^c^
	Small subunit of ribosomal protein	*rps2*, ***rps3*** ^c^, *rps4*, ***rps*7**, ***rps8*** ^c^, *rps11* ^c^, ***rps12*** ^g^, *rps14*, *rps15*, *rps16*, *rps18*, ***rps19*** ^h^
	Subunits of RNA polymerase	*rpoA*, *rpoB*, *rpoC1*, *rpoC2*
**Photosynthesis pathway**	Photosystem I	*psaA*, *psaB*, *psaI*, *psaJ*
	Photosystem I assembly	*ycf3*, *ycf4*
	Photosystem II	*psbA*-*N*, *psbT*, *psb*Z
	F-type ATP synthase	*atpA*, *atpB*, *atpE*, *atpF*, *atpH*, *atpI*
	NADH-plastoquinone oxidoreductase	*ndhA*, ***ndhB*** ^i^, *ndhC*, *ndhD*, *ndhE*, *ndhF*, *ndhG*, *ndhH*, *ndhI*, *ndhJ*, *ndhK*
	Component of cytochrome b6/f Complex	*petA*, *petB*, *petD*, *petG*, *petL*, *petN*
	Carbon metabolism	*cemA*
	Cytochrome c biogenesis protein	*ccsA*
	Large subunit of Rubisco	*rbcL*
**Protein-modifying**	ATP-dependent protease proteolytic subunit	*clpP*
**Structural RNAs**	Transfer RNAs	*trRNA*-Ala (***trnA-UGC***)*, -*Arg (***trnR-ACG*** *, trnR-UCU*), Asn (***trnN-GUU***)^j^ *, -Cys* (*GCA*)*, -*Gln (*trnQ-UUG*)*,-Glu* (*trnE-UUC*)*, -*fMet (*trnfM-CAU*)*, - Gly* (*trnG- GCC, trnG-UCC*)*, -*His (***trnH-GUG***)^d^ *, -*Ile (***trnI-CAU***, ***trnI*-*GAU***), -Lys (*trnK-UUU*), -Leu (***trnL-CAA*** *, trnL-UAA, trnL-UAG*), -Met (*trnM-CAU*), -Phe (*trnF-GAA*), -Pro *(trnP-UGG*), -Ser (*trnS-GCU, trnS-GGA, trnS-UGA*), -Thr (*trnT-GGU, trnT-UGU*), -Trp (trn*W-CCA*), -Tyr (*trnY-GUA*), -Val (***trnV-GAC*** *, trnV-UAC*),
	Ribosomal RNAs	***rrn16***, ***rrn23***, ***rrn4*.*5***, ***rrn5***
**Post-transcriptional modification**	Maturase	*matK*
**Protein of unknown function**	Other genes	***ycf1*** ^k^, ***ycf2***

Boldface for genes duplicated in the IR regions,

a Exon not duplicated in H. ormocarpioides.

b Intron 1 duplicated in S. macrobotrys.

c,d Duplicated in the IR expansion of C. cathartica and S. macrobotrys respectively.

e Duplicated exon 1 and 2 in C. cathartica.

f Duplicated intron 1 in C. cathartica.

g trans-spliced gene.

h Duplicated in the IR of all species except D. araripensis, L. domingensis, O. pinnata, P. violacea, X. stuhlmannii, I. linifolia and tinctoria.

i Duplicated in the IR of C. pubescens and D. falciformis.

j Duplicated in the IR of all species except L. domingensis.

k Duplicated in C. carthatica and L. cuneata.

### Phylogenetic Analysis

A total of 49 plastomes (including 43 species of the MP clade and six outgroups) were used for the phylogenetic analysis. The outgroups included two loosely related species of the subfamily Caesalpinioideae (*Tamarindus indica* L., NC026685, and *Ceratonia siliqua* L., NC026678) with plastome data downloaded from GenBank, and four more closely related species (newly sequenced) of the subfamily Papilionoideae [*Parochetus communis* Buch.-Ham. ex D.Don, *Kotschya aeschynomenoides* (Welw. ex Baker) Dewit & P.A.Duvign., *Pterocarpus violaceus* Vogel, and *Podalyria calyptrata* Willd.]. We could not perform whole plastome alignment due to high PSVs in the legume plastomes. For this reason, we used the python script “get_annotated_regions_from_gb” (https://github.com/Kinggerm/PersonalUtilities) to extract the CDs and NCDs from the plastomes. We performed individual gene/region alignment in MAFFT v.7.4.0 (Katoh and Standley, 2013) with LINSI algorithm. All alignments were visualized and manually adjusted in Geneious. To reduce systematic error, we excluded noncoding loci with less than 70% taxon occupancy or alignment lengths less than 100 bp. We generated three data matrices for the phylogenetic analyses that included the CDs (81 genes for all species), NCDs (113 loci for all species), and CP (concatenated CDs and NCDs for all species).

The substitution models for the three data matrices were determined using PartitionFinder2 v.2.1.1 (Lanfear et al., 2017). The evolutionary best fit models and data partitioning schemes (**Supplementary Table S3**) were selected using the corrected Akaike Information Criterion (AICc). Phylogenetic relationships were reconstructed using Maximum Likelihood (ML) and Bayesian Inference (BI). The ML analysis was performed using the IQ-TREE (Nguyen et al., 2015; Chernomor et al., 2016). We used the best partitioning schemes, -spp option (allowing partition-specific rates), and the ultrafast bootstrap replicates at 1000 for the analyses. The BI was performed using MrBayes v.3.2.6 (Ronquist and Huelsenbeck, 2003). The Bayesian posterior probability (PP) was estimated with two independent Markov Chain Monte Carlo (MCMC) runs, which included one cold chain and three hot chains for 10,000,000 generations and the tree sampling frequency at every 1,000 generations. The MCMC convergence was determined, and the first 20% were discarded as burn*-*in using TRACER v.1.6 (Rambaut and Drummond, 2004). Each parameter for each run obtained a sufficient effective sample size (ESS > 250). The majority-rule consensus tree was generated from the post burn-in trees. The resulting trees (ML and BI) were viewed and edited in FigTree v.1. 3.1 software (Rambaut, 2009).

## Results

### Plastome Organization and Size

**Figure 1:**
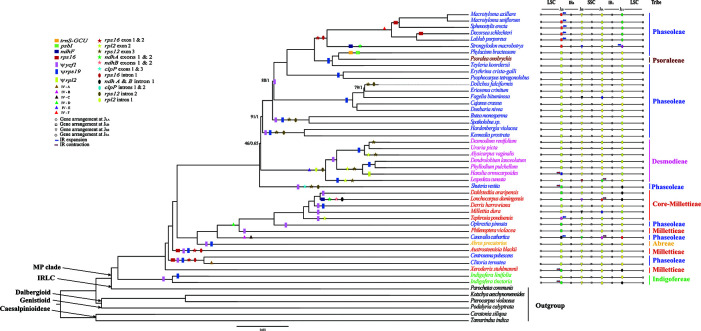
The ML tree of the MP clade reconstructed based on the CP and the variation of IR/SC junctions. Numbers at nodes correspond to ML bootstrap percentages (only values <100% are shown) and Bayesian inference (BI) posterior probabilities (only probabilities <1.0 are shown). Genes loss, pseudogenes, inversions (IV), exon and intron loss, in the plastome, are indicated on the branches using coloured squares, rectangles, triangles, stars and oval shapes, respectively. The IR expansion and contraction are shown by blue and red arrow, respectively.

The mean plastome coverage ranged between 162.0 × (*Philenoptera violacea* (Klotzsch) Schrire, Millettieae) and 1,536.4 × [*Cajanus crassus* (Prain ex King) Maesen, Phaseoleae]. The plastomes of the 43 species from the MP clade exhibited a typical quadripartite structure (**Figure 1**; **Supplementary Figure S1**). The plastome size ranged from 148,889 bp in *Lonchocarpus domingensis* DC. of Millettieae to 160,040 bp in *Indigofera linifolia* (L.f.) Retz. of Indigofereae. Substantial length variation was evident in the LSC, ranging from 77,970 bp in *Canavalia cathartica* Thouars. of Phaseoleae to 90,459 bp in *I. linifolia* of Indigofereae. The SSC length ranged from 14,869 bp in *Strongylodon macrobotrys* A.Gray of Phaseoleae to 18,965 bp in *C. cathartica*. Finally, the IR ranged from 24,111 bp in *Desmodium renifolium* Schindl. of Desmodieae to 30,644 bp in *C. cathartica* (**Supplementary Table S2**). We observed only marginal variation in the GC content, which ranged from 34.2% in *Dolichos falciformis* E.Mey. of Phaseoleae to 35.8% in *Indigofera* spp. of Indigofereae (**Supplementary Table S2**).

Each plastome contained 109–111 unique genes, including 73–90 PCGs, 30 tRNAs, and four rRNAs (**Table 1**). Nine genes (*atpF*, *ndhA*, *ndhB*, *petB*, *petD*, *rpl2*, *rpl16*, *rpoC1*, and *rps16*) had one intron, while two genes (*clpP* and *ycf3*) had two introns (**Table 1**). The *rps12* gene of most species was trans-spliced into three exons (exon 1 in the LSC, and exons 2 and 3 in the IR). Four genes were absent from some species and lineages: the *rps16* gene from *Austrosteenisia blackii* (F.Muell.) R.Geesink*, Centrosema pubescens* Benth.*, Clitoria ternatea* L.*, Decorsea schlechteri* (Harms) Verdc*., Macrotyloma axillare* (E.Mey.) Verdc., *Macrotyloma uniflorum (*Lam.) Verdc., and *Sphenostylis erecta* Hutch. ex Baker f. (Phaseoleae); the *psbI* and *trnS-GCU* genes from *Phylacium bracteosum* Benn. (Phaseoleae); and the *ndhF* gene from *L. domingensis* (Millettieae) and *S. macrobotrys* (Phaseoleae). We detected pseudogenization of *ycf1, rpl2,* and *rps19* in one to multiple species (**Figure 1**; **Supplementary Figure S2**).

### Plastome Structural Variations in the MP Clade

**Figure 2:**
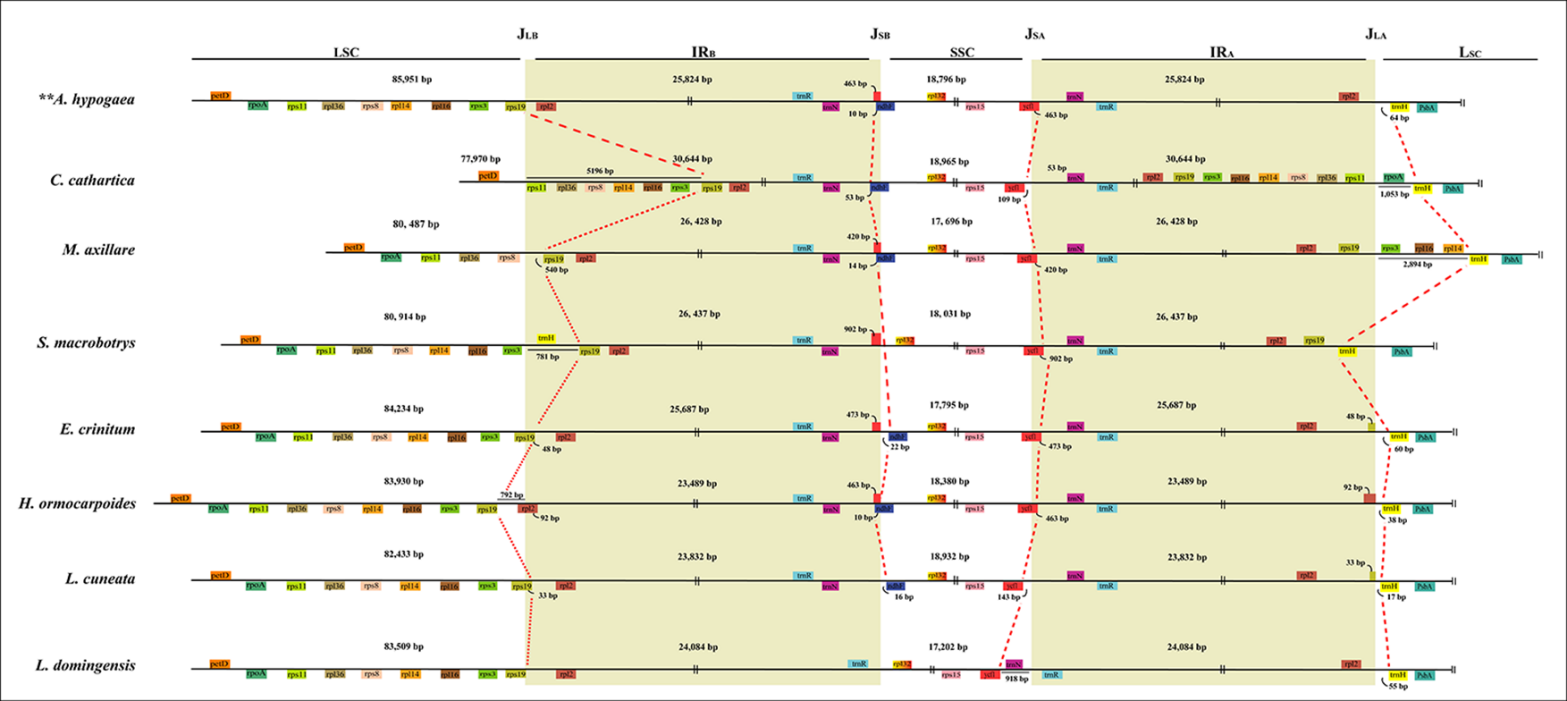
Comparison of LSC, IRs, and SSC junction positions among plastomes of the MP clade. J_LB_, J_SB_, J_SA_, J_LA_ refer to junctions of LSC/IR_B_, SSC/IR_B_, SSC/IR_A_, LSC/IR_A_, respectively.

The locations of IR-SC junctions in many species of the MP clade have experienced significant variations in some species (**Figures 1** and **2**; **Supplementary Figure S2**). Mostly, the SSC/IR_B_ (J_SB_) border lies within the *ndhF* gene, with the duplication of 3’-ends of this gene (from 1 bp in *Psophocarpus tetragonolobus* DC. to 53 bp in *C. cathartica*) at the boundary of the IRA/SSC junction (J_SA_). However, some species contracted their IRs following the shift of the J_SB_ into the IGS region. The J_SB_ lies within the IGS region between *trnN* and *rpl32* in *S*. *macrobotrys* because of the loss of *ndhF*. Instead, the J_SB_ lies within the IGS region between *trnR* and *rpl32* in *L. domingensis* because of the loss of *ndhF* and the translocation of *trnN* into the SSC region. The J_SA_ lies within the *ycf1* gene in most species, with the duplication of 3’-ends of this gene (from 374 bp in *Shuteria vestita* Wight & Arn. to 1,240 bp in *Erythrina crista-galli* L.) at the boundary of the J_SB_. The IR is contracted at this boundary following the shift of J_SA_ into the IGS region between *ycf1* and *trnN* in *Lespedeza cuneata* G.Don and *C. cathartica*, between *trnN* and *trnR* in *L*. *domingenesis*.

Typically, the LSC/IR_B_ junction (J_LB_) lies within the *rps19* gene, resulting in the duplication of the 5’-ends of this gene (from 2 bp in *A. blackii* to 68 bp in *E. crista-galli*) at the boundary of the IR_A_/LSC junction (J_LA_). The J_LB_ has experienced expansion into the LSC by 5,196-bp in *C*. *cathartica* to include the intact *rps3, rps8, rps11, rpl36, rps14, rps16,* and *rps19* genes. The J_LB_ lies between *petD* and *rps11* in *C*. *cathartica* and between *rps19* and *rps8* in *M. axillare*, *M. uniflorum*, *S. erecta*, *D. schlechteri*, and *Lablab purpureus* (L.) Sweet. Also, the J_LB_ lies between *rps3* and *trnH* in *S. macrobotrys*, and between *rps3* and *rps19* in *Tephrosia pondoensis* (Codd) Schrire. Likewise, the J_LB_ has experienced contraction into *rpl2* in *Hanslia ormocarpoides* (DC.) H. Ohashi, and the IGS region between *rps19* and *rpl2* in *S. vestita*, *Dahlstedtia araripensis* (Benth.) M.J.Silva & A.M.G.Azevedo, *L. domingensis*, *Ophrestia pinnata* (Merr.) Verdc., *P. violacea*, *Xeroderris stuhlmannii* (Taub.) Mendonça & E.P.Sousa, *Indigofera tinctoria* Gouan and *Millettia dura* Dunn. The J_LA_ is mostly between *rps19* and *rpl2* in the IR and *trnH* in the LSC. However, the J_LA_ lies between *rps11* and *rpoA* in *C. cathartica*, between *rps19* and *rps3* in *M. axillare*, *M. uniflorum*, *S. erecta*, *D. schlechteri* and *L. purpureus*, and between *trnH* and *psbA* in *S. macrobotrys*.

**Figure 3:**
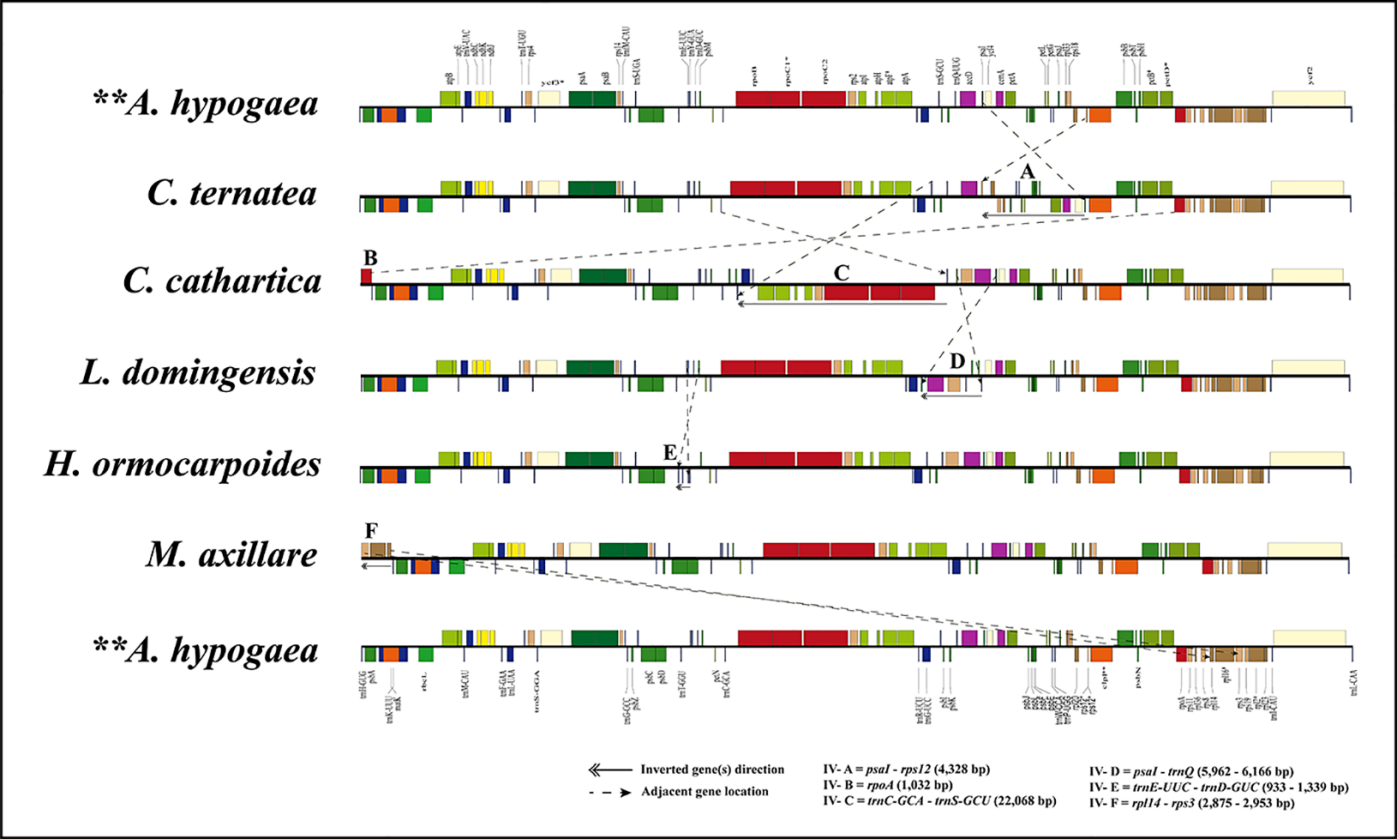
Plastome inversions in representative species of the MP clade. Gene arrangement as the reference plastome of *Arachis hypogaea*.

Multiple inversions (IVs A to F) and intragenomic relocations were detected in the LSC region of some species in the MP clade (**Figure 3**; **Supplementary Figure S3**), including a 4,328-kb inversion (IV A) from *psaI* to *rps12* in *C. ternatea*; a 1,032-kb inversion (IV B) of the *rpoA* gene; a 22,060-kb inversion (IV C) from *trnC-GCA* to *trnS-GCU* in *C. cathartica*; a 5,962- to 6,166-bp inversion (IV D) from *trnQ-UUG* to *psaI* in the *P. violacea* + *O. pinnata* + *T. pondoensis* + *M. dura* + *Derris harrowiana* + *L. domingensis* + *D. araripensis* clade; a 933- to 1,339-bp inversion (IV E) from *trnE-UUC* to *trnD-GUC* in the *L. cuneata* + *H. ormocarpoides* + *Phyllodium pulchellum* (L.) Desv. + *Dendrolobium lanceolatum* (Dunn) Schindl. + *Alysicarpus vaginalis* (Schumach.) J.Léonard + *Uraria picta* (Jacq.) Desv. ex DC. + *D. renifolium* clade; and a 2,875- to 2,952-kb inversion (IV F) from *rpl14* to *rps3* in the *L. purpureus* + *D. schlechteri* + *S. erecta* + *M. uniflorum* + *M. axillare* clade. Interestingly, the *rpoA* gene translocated from one end of the LSC near the J_LB_ to another end of the LSC near the J_LA_ in *C. cathartica*. Additionally, the segment comprising the genes *rpl14*, *rpl16* and *rps3* was translocated from one end of the LSC near the J_LB_ to another end of the LSC near J_LA_ in a subclade of the tribe Phaseoleae (*L. purpureus* + *D. schlechteri* + *S. erecta* + *M. uniflorum* + *M. axillare*).

### Phylogenetic Relationships of the MP Clade

**Figure 4:**
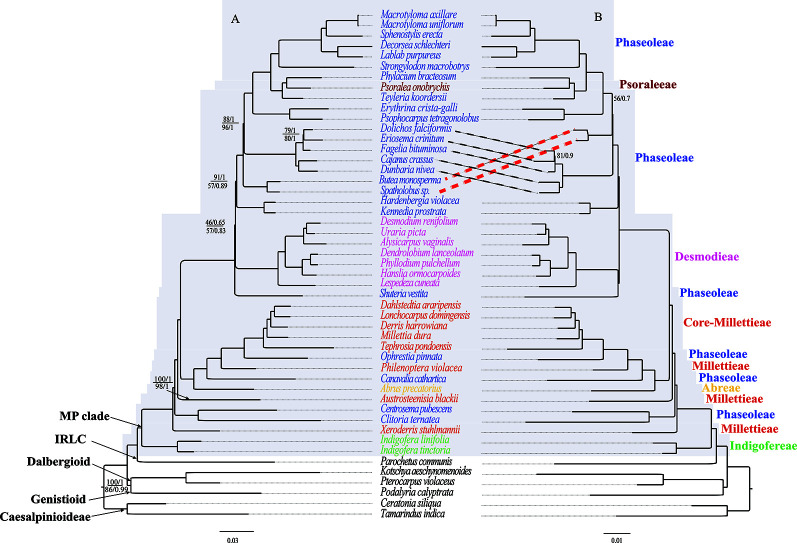
The ML and BI phylogenetic relationships reconstructed for the MP clade. **(A)** CP and NCDs, and **(B)** CDs. Numbers at nodes correspond to ML bootstrap percentages (only values <100% are shown) and Bayesian inference (BI) posterior probabilities (only probabilities <1.0 are shown). For **(A)**, the values above and below the line represents support values for the CP and NCDs, respectively. The thick dotted lines indicate topology differences. The scale bar represents the mean nucleotide substitutions per site along the branch.

The phylogenies of the MP clade inferred from the three data matrices and two methods (ML and BI) yielded largely similar topologies, including well-resolved deep relationships of the MP clade (**Figure 4**). Our phylogenetic analyses strongly supported (BS ≥ 95 %, and PP = 1.0) the monophyly of the MP clade and most lineages. However, the lineage consisting of *Butea monosperma* (Lam.) Kuntze and *Spatholobus* Hassk sp. has different phylogenetic position in trees of CP and NCDs, and that of CDs, but both relationships were weakly supported. Also, the tribe Desmodieae was weakly supported to bemonophyletic in CP and NCDs datamatrices whereas strongly supported by CDs data. The tribe Indigofereae was strongly supported as sister to the remainder of the MP clade (BS = 100%, and PP = 1.0). Based on the current sampling, it is not sure if the tribe Desmodieae is monophyletic, while the tribes Millettieae and Phaseoleae appear non-monophyletic. *Psoralea onobrychis* Nutt. of the tribe Psoraleeae was nested within a big clade of the tribe Phaseoleae.

## Discussion

### Evolutionary Pattern of PSV in the MP Clade

#### Gene Loss and Pseudogenization Events

Previous studies documented the loss of the genes *rpl22* and *inf*A in *Lotus japonicus* (Regel) K.Larsen of the Robinioid clade (Kato et al., 2000), *Trifolium subterraneum* L. of the IRLC (Cai et al., 2008), and *G. max* of the MP clade (Saski et al., 2005); this study confirmed the loss of both genes in all studied species of this clade. These two genes (*rpl22* and *inf*A) were reported lost in all the previously studied legume species (Saski et al., 2005) and almost all rosids (Millen et al., 2001). The functional copies of *rpl22* and *infA* might have been transferred into nuclear genome [e.g., *Pisum sativum* L., (Gantt et al., 1991); *Lupine* L. species, (Martin et al., 2014)]. Previous studies suggested the loss of the *ycf4* gene in *Cicer* L. sp., *Glycine* Wild. sp., and *Medicago* L. sp. (Magee et al., 2010; Kaila et al., 2016), or as pseudogene in *P*. *sativum* (Smith et al., 1991). Interestingly, we found *ycf4* to be a normal gene in all newly sequenced plastomes of the species from the MP clade. We therefore attribute the absence of this gene in previous studies to inaccurate genome annotation, as the *ycf4* gene is highly divergent (Kaila et al., 2016). The loss of the *rps16* gene has been reported in some legumes (Doyle et al., 1995). Again, we detected the loss of this gene in *C. pubescens*, *C. ternatea*, *D. schlechteri*, *S. erecta*, *M. uniflorum* and *M. axillare* of the tribe Phaseoleae of the MP clade (**Figure 1**).

The loss of introns (e.g., *rpl2* intron 1) has occurred frequently in the plastomes of some angiosperm families as Convolvulaceae, Menyanthaceae, and Saxifragaceae (Downie et al., 1991), Leguminosae (Lee and Hymowitz, 2001; Jansen et al., 2008), and Lythraceae (Gu et al., 2016). Introns, especially those located at specific regions, are momentous in the transformational functionality and regulation of gene expressions (Xu et al., 2003). According to this study, with the exception of the loss of the *clpP* introns 1 and 2 in a single species of *S. vestita* (Phaseoleae) and the loss of *ndh A* and *ndh B* intron 1 in a single species of *L. domingensis* (Millettieae), two other introns (*rps16* and *rps12*) have experienced multiple independent loss during the plastome evolution of the species from the MP clade. This finding agrees with the previous studies on the independent loss of *rpsl2*, *rps16*, and *clpP* introns in the MP clade (Guo et al., 2007; Schwarz et al., 2015; Kaila et al., 2016).

Consistent with previous studies in legumes, we observed the *rps12* gene to have been trans-spliced (located in LSC region and the duplicated end in IR_A_) during the plastome evolution of the species from the MP clade (Fonseca and Lohmann, 2017; Wang et al., 2017). Our results showed the expression of two distinct transcripts from a single gene. Previously, the *rps12* gene ligation between exon 1 and 2 had been affirmed through complementary DNA sequencing of *rps12* messenger RNA (mRNA) (Sharp, 1985). Thus, this evidence suggests that the *rps12* gene was trans-spliced (exon 1 and exons 2–3) because of separate transcription. Trans-spliced events of a single gene during evolution are linked with two distinct transcripts encoding protein structural domains (Sharp, 1985) and reverse transcription of the trans-spliced, sequel to the insertion in the plastome (Baltimore, 1985). The exon-rearrangement paradigm during gene evolution propounds that gene fragments coding for protein structural domains (exon) are affected by reorganization into other genes (Gilbert et al., 1986). Also, RNA trans-splicing coding for *rpsl2* exon 1 with transcripts from other genes may yield polypeptide variations in the plastome. These may be the underlying factor responsible for the *rps12* gene trans-splicing event in the plastomes of the species from the MP clade.

Previous studies have documented pseudogenes in some species of the MP clade, for example *rps16* and *rpl33* in *P. vulgaris* (Guo et al., 2007); *ycf15*, *rpl33*, *rps16*, *ycf68* and *ycf1* in *Cajanus scarabaeoides* (L.) Thouars (Kaila et al., 2016); and *rps16* in *Lupinus* (Keller et al., 2017). Our study identified *rpl2*, *rps19,* and *ycf1* as pseudogenes (based on the presence premature stop codons and their reduced length) in most species of the MP clade (**Figure 3**; **Table 1**), while the *rps16* and *rpl33* genes were detected as normal genes in the species of the MP clade. The pseudogenization of these genes has been reported in other species, e.g. *Melianthus villosus* Bolus in Melianthaceae (Weng et al., 2014), *Phalaenopsis aphrodite* Rchb.f. in Orchidaceae (Chang et al., 2006), and *Tylosema* spp. in Mimosoideae (Wang et al., 2017). Pseudogenization of some genes is common in the plastomes of some plant taxa (Kim et al., 2015; Naumann et al., 2016; Keller et al., 2017). In previous studies, gene loss/pseudogenization in the plastome is attributed to rate of sequence evolution, gene transfer to the nucleus, or substitution by a nuclear-encoded protein for a plastid gene product (Ueda et al., 2008; Magee et al., 2010; Jansen and Ruhlman, 2012; Williams et al., 2015).

#### IR Contraction and Expansion

IR-SC boundary shifts played a significant role in the plastome size variation of the species from the MP clade (**Figure 1**; **Supplementary Figure S2**). Significantly, a substantial expansion of the IR to include six ribosomal protein genes (*rps3*, *rps8*, *rps11*, *rpl14*, *rpl16* and *rpl36*) resulted in the large plastome of *C*. *cathartica* (Phaseoleae) (**Figure 2**; **Supplementary Figure S2**). In contrast, in *L. domingensis* (Millettieae), the *trnN* and *ycf1* genes have been relocated into the SSC following IR contraction, resulting in the smallest plastome studied of the MP clade. Additionally, the contraction/expansion of IR regions in the MP clade accounts for new positions of J_LA_ between *rps11* and *rpoA*; *rps19* and *rps3*, and *trnH* and *psbA*.

The IR contraction/expansions are frequent evolutionary events in angiosperm lineages, resulting in dramatic differences in the plastome length variations (e.g., Guisinger et al., 2011; Zeng et al., 2017). The rate of gene conversion during cell division/evolution and high content of short repeats (AT-rich) have also been noted as explanations for IR boundary shifts among several angiosperm lineages (Wang et al., 2008; Dugas et al., 2015; Wang et al., 2017). The same mechanisms might explain IR boundary shifts in plastomes of the species from the MP clade. The IR expansion to include the whole *rps19* gene is a synapomorphic character for the *M. axillare* + *M. uniflorum* + *S. erecta* + *D. schlechteri* + *L. purpureus* clade. Most other IR contractions/expansions occurred independently across the MP clade.

Gene relocation within plastome has been reported in multiple previous studies (e.g., Lee et al., 2007; Kaila et al., 2016; Mower et al., 2019). For instance, the intragenomic transfer of *ycf2* from the LSC region to the SSC region in lycophytes (Mower et al., 2019), the relocations of *ycf3* and *ycf4* within the LSC region of *Menodora longiflora* Engelm. ex A.Gray (Oleaceae, Lee et al., 2007), and the transfer of a block of ribosomal protein genes (*rps19*–*rps8*) from one end of the LSC region to the other end in the legumes— e.g. *Vigna* Sav*i* (Perry et al., 2002), *Phaseolus* L. (Bruneau et al., 1990) and *Cajanus* spp. (Kaila et al., 2016). Similarly, our study detected translocation of genes within the LSC region in the plastomes of multiple species from the MP clade (**Figure 3**). Additionally, we documented the relocation of a single gene (*rpoA*) in *C*. *cathartica*, and three ribosomal protein genes (*rpl14*, *rpl16* and *rps3*) in a clade of Phaseoleae from one end of the LSC region to the other. Gene relocation can be associated with the subsequent contraction and expansion of the IR as observed in *Pelargonium* L’Hér. ex Aiton (Bruneau et al., 1990; Chumley et al., 2006). Alternatively, overlapping inversions and IR direction have been applied to explain the relocation of genes in the plastome of Oleaceae (Lee et al., 2007) and lycophytes (Mower et al., 2019), respectively. The IR expansion to include these genes is followed by the IR contraction at another end to relocate these genes into the SSC region. This appears to represent a more parsimonious explanation for the relocation of the *rpoA* gene and the segment comprising the genes *rpl14*, *rpl16* and *rps3*.

#### Inversions

Several inversions including a 421-bp inversion in the mimosoid species (Wang et al., 2017), a 7.5-kb inversion in the Cercioideae (Kim and Cullis, 2017), and a large inversion of 50-kb in the subfamily Papilionoideae (Guo et al., 2007; Cai et al., 2008; Keller et al., 2017) occur in legumes. A few studies have documented the presence of inversions in species of the MP clade, such as *V. radiata* (Jansen et al., 2007), *L. luteus* (Martin et al., 2014), and *P. vulgaris* (Bruneau et al., 1990). Importantly, an early molecular investigation (Bruneau et al., 1990) on plastome DNA inversions in Papilionoideae detected a large inversion (78-kb in size) between the *psbA* and *rps11* genes in nine species of the tribe Phaseoleae. Also, prior studies documented a 50-kb inversion that spans the genes *rbcL* and *rps16* in the plastomes of *C. cajan* and *C*. *scarabaeoides* (Kaila et al., 2016) and *Cyamopsis tetragonoloba* (L.) Taub. (Kaila et al., 2017) in the MP clade. By analyzing additional taxa of the MP clade, we discovered six new inversions in three tribes (Desmodieae, Milletieae, and Phaseoleae) of the MP clade (**Figure 1**; **Supplementary Figure S3**), with the largest size being 22-kb (IV-C, **Figure 3**). These newly discovered inversions significantly increase the number of documented plastome rearrangements in Leguminosae.

Inversions might be linked with IR contraction/expansion (Bruneau et al., 1990), as shown by IV-A, B, and F in the study. The regions flanking three inversions (IV-C, D, and E) contain tRNA genes, which is consistent with the assumption that tRNA activity may influence inversion in plastome (Walker et al., 2014). Also, recombination through repeated sequences can induce inversions in plastome (Rogalski et al., 2006). We failed to detect any repeats in the breakpoint regions of these six inversions. Rearrangements such as inversions in plastid genomes of land plants are considered a useful marker to infer evolutionary relationships (Doyle et al., 1992). Large inversions have been considered informative for defining clades in legumes (Bruneau et al., 1990; Doyle et al., 1996; Dugas et al., 2015). For example, the inversion (IV-E) is synapomorphy of the monophyletic tribe Desmodieae excluding *S*. *vestita*. The IV-D occurs multiple times in tribes Millettieae and Phaseoleae. The other four inversions (IV-A, B, C, and F) occur in multiple separate lineages of Phaseoleae.

### Phylogenetic Relationships in the MP Clade

Appropriate data partitioning is important for achieving accurate phylogenetic result in simultaneous utilization of multiple genes (Li et al., 2013; Saarela et al., 2018; He et al., 2019), a way may greatly abate the erroneous phylogenetic inferences caused by unequal rates and patterns of nucleotide substitutions in plastomes (Li et al., 2008). Our results indicated that ML and BI analyses with multiple genes partitioned models (CDs, NCDs, and CP) presents well-resolved evolutionary relationships of the MP clade. This study underscores the utility of plastid phylogenomics for resolving intertribal and intergeneric relationships within the MP clade (**Figure 4**). Evolutionary relationships among the major lineages, tribes, and genera were resolved with high support values. Consisted with previous studies (Hu et al., 2000; Wojciechowski et al., 2004; Cardoso et al., 2013; de Queiroz et al., 2015; LPWG, 2017), our analyses supported the tribe Indigofereae as sister to the remaining members of the MP clade. Desmodieae was supported as monophyletic group in previous studies (Bruneau et al., 1994; Doyle et al., 1997; Kajita et al., 2001; Stefanovic et al., 2009; Cardoso et al., 2013; de Queiroz et al., 2015; Egan et al., 2016), however this tribe was strongly supported as monophyletic by CDs but weakly supported by CP and NCDs (**Figure 4**). Our phylogenetic analyses suggested the polyphyly of the tribes Millettieae and Phaseoleae, which are consistent with previous studies (Hu et al., 2000; Wojciechowski et al., 2004; de Queiroz et al., 2015; Vatanparast et al., 2018). Previous studies (Wojciechowski et al., 2004; Cardoso et al., 2013; de Queiroz et al., 2015; LPWG, 2017) included multiple genera and supported the monophyly Psoraleeae. The phylogenetic analysis of Stefanovic et al. (2009) based on eight plastid genes supported the tribe Psoraleeae as sister to Phaseoleae, whereas it is nested within the Phaseoleae in this study and several other studies (e.g., Hu et al., 2000; de Queiroz et al., 2015; Vatanparast et al., 2018).

Our study benefits from having a more comprehensive taxon sampling and involving whole plastome sequences for phylogenetic analysis; thus, it marks the beginning of a better understanding of evolutionary relationships in the MP Clade. For instance, our study highly supported the relationships of (1) *C*. *ternatea* + C. *pubescens* (BS = 100/PP = 1) and (2) *A*. *blackii* + *C*. *ternatea* + *C*. *pubescens* (BS = 100/PP = 1); these relationships were only weakly supported in previous studies (Kajita et al., 2001; Vatanparast et al., 2018). Notably, our multi-locus plastome data suggested (BS = 100%, PP = 1) the evolutionary position of *S. vestita* as sister to the tribe Desmodieae, in contrast with previous placement close to the subtribe Kennediinae of the tribe Phaseoleae (e.g., de Queiroz et al., 2015). Formerly, the genus *Shuteria* was included in the tribe Phaseoleae based on flower structures shared with core Phaseoleae species (e.g., *Amphicarpaea* Elliott ex Nutt., *Cologania* Kunth, and *Dumasia* DC., Lackey et al., 1981). It is noteworthy that a similar phylogenetic placement in the MP clade has been shown from analysis based on the single plastid region *matK* (de Queiroz et al., 2015). Therefore, our phylogeny supports the placement of *S. vestita* as sister to the tribe Desmodieae. Nevertheless, we expect that future phylogenetic studies would improve the understanding of the phylogenetic relationships of the genus Shuteria within the clade. Collectively our results provide important insights on the backbone relationships of the MP clade. However, additional phylogenetic study, perhaps integrating additional molecular data with morphological traits, will be necessary to fully clarify the evolutionary relationships of this clade.

### Insights Into the Plastomic Evolution of the MP Clade

Some large inversions in the MP clade seem to have phylogenetic signal for the MP clade (**Figure 1**). The IV-A was only found in *C*. *ternatea*, IV-B and IV-C only in *C*. *cathartica*, and IV-F in the clade of *L*. *purpureus* + *M*. *axillare* + *M. uniflorum* + *S. erecta* + *D*. *schlechteri*. The IV-E was only detected in the tribe Desmodieae, which supports the monophyly of the tribe. Of note, the IV-D is a synapomorphy of one subclade of the tribes Phaseoleae and core-Millettieae, which is congruent with their closely related evolutionary relationships. Consistent with some previous studies (Martin et al., 2014; Dugas et al., 2015; Choi and Choi, 2017), our results suggest that significant plastome structural rearrangements such as inversion may provide useful information about phylogenetic relationships. However, some previous studies have suggested caution in using inversions in phylogenetic analysis. For example, a 36-kb inversion has been documented in distantly related lineages of papilionoids (Schwarz et al., 2015). Also, a 29-kb inversion has been reported from distantly related species of Ranunculaceae (*Anemone* L. and *Clematis* L., Hoot and Palmer, 1994). Additional sampling is necessary to better evaluate the utility of large PSVs for phylogenetic reconstruction in the MP clade. The independent loss of genes, exons, and introns was observed across different lineages of the MP clade. These results are consistent with previous studies that have shown multiple independent losses of specific genes in plastomes of different plant groups (e.g., Gu et al., 2016; Kaila et al., 2016). These kinds of PSV therefore seem to have low phylogenetic signal. Similarly, pseudogenization events have occurred independently across the MP lineages, indicating that these as well are likely not useful for inferences of phylogenetic relationships. Many observed PSVs in the MP clade plastomes suggest significant structural variation following the diversification of this lineage. In total, this study provides new insights into the phylogenetic relationships and PSVs within the MP clade.

## Data Availability Statement

The datasets generated for this study can be found in Genbank; the list of accession can be found in **Supplementary Table 1**.

## Author Contributions

T-SY, OO, and RZ designed the research. OO and RZ performed the experiments and assembled the plastomes. OO, RZ, and S-YC conducted the analysis. OO and T-SY wrote the manuscript. All authors revised the manuscript and approved the final manuscript.

## Funding

This study was supported by grants from the Large-scale Scientific Facilities of the Chinese Academy of Sciences (No. 2017-LSF-GBOWS-02), the Strategic Priority Research Program of Chinese Academy of Sciences (XDB31010000), the National Natural Science Foundation of China [key international (regional) cooperative research project No. 31720103903].

## Conflict of Interest

The authors declare that the research was conducted in the absence of any commercial or financial relationships that could be construed as a potential conflict of interest.

## References

[B1] Asaf S. Khan A. L. Khan M. A. Imran M. Q. Kang S. M. Al-Hosni K.. (2017). Comparative analysis of complete plastid genomes from wild soybean (*Glycine soja*) and nine other *Glycine* species. PloS One 12, e0182281. 10.1371/journal.pone.0182281 28763486 PMC5538705

[B2] Baker M. (2004). Wood for Woodturners (Sussex: Guild of Master Craftsmen Publications).

[B3] Baltimore D. (1985). Retroviruses and retrotransposons: the role of reverse transcription in shaping the eukaryotic genome. Cell 40, 481–482. 10.1016/0092-8674(85)90190-4 2578883

[B4] Bankevich A. Nurk S. Antipov D. Gurevich A. A. Dvorkin M. Kulikov A. S.. (2012). SPAdes: a new genome assembly algorithm and its applications to single-cell sequencing. J. Comput. Biol. 19, 455–477. 10.1089/cmb.2012.0021 22506599 PMC3342519

[B5] Bock R. (2007). “Structure, function, and inheritance of plastid genomes,” in Cell and molecular biology of plastids, vol. 29–63 . Ed. Bock R. (Berlin, Heidelberg: Springer Berlin Heidelberg).

[B6] Bruneau A. Doyle J. J. Palmer J. D. (1990). A chloroplast DNA Inversion as a subtribal character in the phaseoleae (Leguminosae). *Syst* . Botany 15, 378–386. 10.2307/2419351

[B7] Bruneau A. Doyle J. J. Doyle J. A. (1994). “Phylogenetic relationships in Phaseoleae: evidence from chloroplast DNA restriction site characters,” in Advances in legume systematics, Part 7. Eds. Crisp M. Doyle J. J. (Richmond, Surrey, UK: Royal Botanic Gardens, Kew), 309– 330.

[B8] Cai Z. Guisinger M. Kim H. G. Ruck E. Blazier J. C. McMurtry V.. (2008). Extensive reorganization of the plastid genome of *Trifolium subterraneum* (Fabaceae) is associated with numerous repeated sequences and novel DNA insertions. J. Mol. Evol. 67, 696–704. 10.1007/s00239-008-9180-7 19018585

[B9] Cardoso D. Pennington R. T. de Queiroz L. P. Boatwright J. S. Van Wyk B. E. Wojciechowski M. F.. (2013). Reconstructing the deep-branching relationships of the papilionoid legumes. S. Afr. J. Bot. 89, 58–75. 10.1016/j.sajb.2013.05.001

[B10] Chang C. C. Lin H. C. Lin I. P. Chow T. Y. Chen H. H. Chen W. H.. (2006). The chloroplast genome of *Phalaenopsis aphrodite* (Orchidaceae): comparative analysis of evolutionary rate with that of grasses and its phylogenetic implications. Mol. Biol. Evol. 23, 279–291. 10.1093/molbev/msj029 16207935

[B11] Chernomor O. von Haeseler A. Minh B. Q. (2016). Terrace aware data structure for phylogenomic inference from supermatrices. Syst. Bot. 65, 997–1008. 10.1093/sysbio/syw037 PMC5066062 27121966

[B12] Choi I. S. Choi B. H. (2017). The distinct plastid genome structure of *Maackia fauriei* (Fabaceae: Papilionoideae) and its systematic implications for Genistoids and tribe Sophoreae. PloS One 12, e0173766. 10.1371/journal.pone.0173766 28399123 PMC5388331

[B13] Chumley T. W. Palmer J. D. Mower J. P. Fourcade H. M. Calie P. J. Boore J. L.. (2006). The complete chloroplast genome sequence of *Pelargonium* x *hortorum*: organization and evolution of the largest and most highly rearranged chloroplast genome of land plants. Mol. Biol. Evol. 23, 2175–2190. 10.1093/molbev/msl089 16916942

[B14] Cosner M. E. Jansen R. K. Palmer J. D. Downie S. R. (1997). The highly rearranged chloroplast genome of *Trachelium caeruleum* (Campanulaceae): multiple inversions, inverted repeat expansion and contraction, transposition, insertions/deletions, and several repeat families. Curr. Genet. 31, 419–429. 10.1007/s002940050225 9162114

[B15] Darling A. C. E. Mau B. Blattner F. R. Perna N. T. (2004). Mauve: Multiple alignment of conserved genomic sequence with rearrangements. Genome Res. 14, 1394–1403. 10.1101/gr.2289704 15231754 PMC442156

[B16] de Queiroz L. P. Pastore J. F. Cardoso D. Snak C. de C Lima A. L. Gagnon E.. (2015). A multilocus phylogenetic analysis reveals the monophyly of a recircumscribed papilionoid legume tribe diocleae with well-supported generic relationships. Mol. Phylogenet. Evol. 90, 1–19. 10.1016/j.ympev.2015.04.016 25934529

[B17] Downie S. R. Olmstead R. G. Zurawski G. Soltis D. E. Soltis P. S. Watson J. C.. (1991). Six independent losses of the chloroplast DNA *rpl2* intron in dicotyledons: molecular and phylogenetic implications. Evolution 45, 1245–1259. 10.1111/j.1558-5646.1991.tb04390.x 28564183

[B18] Doyle J. J. Doyle J. L. (1987). A rapid DNA isolation procedure for small quantities of fresh leaf tissue. Phytochemical Bull. 19, 11–15.

[B19] Doyle J. J. Davis J. I. Soreng R. J. Garvin D. Anderson M. J. (1992). Chloroplast DNA inversions and the origin of the grass family (Poaceae). Proc. Natl. Acad. Sci. U. S. A. 89, 7722–7726. 10.1073/pnas.89.16.7722 1502190 PMC49783

[B20] Doyle J. J. Doyle J. L. Palmer J. D. (1995). Multiple independent losses of two genes and one intron from legume chloroplast genomes. Syst. Bot. 20, 272–294. 10.2307/2419496

[B21] Doyle J. J. Doyle J. L. Ballenger J. A. Palmer J. D. (1996). The distribution and phylogenetic significance of a 50-kb chloroplast DNA inversion in the flowering plant family leguminosae. Mol. Phylogenet. Evol. 5, 429–438. 10.1006/mpev.1996.0038 8728401

[B22] Doyle J. J. Doyle J. L. Ballenge J. A. Dickson E. E. Kajita T. Ohashi H. (1997). A phylogeny of the chloroplast gene rbcL in the Leguminosae: taxonomic correlations and insights into the evolution of nodulation. Am. J. Bot. 84, 541–554. 10.2307/2446030 21708606

[B23] Dugas D. V. Hernandez D. Koenen E. J. M. Schwarz E. Straub S. Hughes C. E.. (2015). Mimosoid legume plastome evolution: IR expansion, tandem repeat expansions, and accelerated rate of evolution in *clpP* . Sci. Rep. 5, 16958. 10.1038/srep16958 26592928 PMC4655330

[B24] Egan A. N. Vatanparast M. Cagle W. (2016). Parsing polyphyletic *Pueraria*: delimiting distinct evolutionary lineages through phylogeny. Mol. Phylogenet. Evol. 104, 44–59. 10.1016/j.ympev.2016.08.001 27495827

[B25] Feng L. Gu L. F. Luo J. Fu A. S. Ding Q. Yiu S. M.. (2017). Complete plastid genomes of the genus *Ammopiptanthus* and identification of a novel 23-kb rearrangement. Conserv. Genet. Resour. 9, 647–650. 10.1007/s12686-017-0747-8

[B26] Fonseca L. H. M. Lohmann L. G. (2017). Plastome rearrangements in the “*Adenocalymma*-*Neojobertia*“ clade (Bignonieae, Bignoniaceae) and its phylogenetic implications. Front. Plant Sci. 8, 1875. 10.3389/fpls.2017.01875 29163600 PMC5672021

[B27] Gantt J. S. Baldauf S. L. Calie P. J. Weeden N. F. Palmer J. D. (1991). Transfer of *rpl22* to the nucleus greatly preceded its loss from the chloroplast and involved the gain of an intron. EMBO J. 10, 3073–3078. 10.1002/j.1460-2075.1991.tb07859.x 1915281 PMC453023

[B28] Gilbert W. Marchionni M. McKnight G. (1986). On the antiquity of introns. Cell 46, 151–153. 10.1016/0092-8674(86)90730-0 2424613

[B29] Givnish T. J. Spalink D. Ames M. Lyon S. P. Hunter S. J. Zuluaga A.. (2015). Orchid phylogenomics and multiple drivers of their extraordinary diversification. Proc. R. Soc B. 282, 1–10. 10.1098/rspb.2015.1553 PMC4571710 26311671

[B30] Green B. R. (2011). Chloroplast genomes of photosynthetic eukaryotes. Plant J. 66, 34–44. 10.1111/j.1365-313X.2011.04541.x 21443621

[B31] Gu C. Tembrock L. R. Johnson N. G. Simmons M. P. Wu Z. (2016). The complete plastid genome of *Lagerstroemia fauriei* and loss of *rpl2* intron from *Lagerstroemia* (Lythraceae). PloS One 11, e0150752. 10.1371/journal.pone.0150752 26950701 PMC4780714

[B32] Guisinger M. M. Kuehl J. V. Boore J. L. Jansen R. K. (2011). Extreme reconfiguration of plastid genomes in the angiosperm family Geraniaceae: rearrangements, repeats, and codon usage. Mol. Biol. Evol. 28, 583–600. 10.1093/molbev/msq229 20805190

[B33] Guo X. Castillo-Ramírez S. González V. Bustos P. Fernández-Vázquez J. L. Santamaría R. I.. (2007). Rapid evolutionary change of common bean (*Phaseolus vulgaris* L.) plastome, and the genomic diversification of legume chloroplasts. BMC Genom. 8, 228. 10.1186/1471-2164-8-228 PMC1940014 17623083

[B34] Haberle R. C. Fourcade H. M. Boore J. L. Jansen R. K. (2008). Extensive rearrangements in the chloroplast genome of *Trachelium caeruleum* are associated with repeats and tRNA genes. J. Mol. Evol. 66, 350–361. 10.1007/s00239-008-9086-4 18330485

[B35] He J. Yao M. Lyu R. D. Lin L. L. Liu H. J. Pei L. Y.. (2019). Structural variation of the complete chloroplast genome and plastid phylogenomics of the genus *Asteropyrum* (Ranunculaceae). Sci. Rep. 9, 15285. 10.1038/s41598-019-51601-2 31653891 PMC6814708

[B36] Hoot S. B. Palmer J. D. (1994). Structural rearrangements, including parallel inversions, within the chloroplast genome of Anemone and related genera. J. Mol. Evol. 38, 274–281. 10.1007/bf00176089 8006994

[B37] Hu J. M. Lavin M. Wojciechowski M. F. Sanderson M. J. (2000). Phylogenetic systematics of the tribe Millettieae (Leguminosae) based on *trnK*/*matK* sequences, and implications for evolutionary patterns in Papilionoideae. Am. J. Bot. 87, 418–430. 10.2307/2656638 10719003

[B38] Hu J. M. Lavin M. Wojciechowski M. F. Sanderson M. J. (2002). Phylogenetic analysis of nuclear ribosomal ITS/5.8 S sequences in the tribe Millettieae (Fabaceae): *Poecilanthe*-*Cyclolobium*, the core Millettieae, and the *Callerya* group. Sys. Bot. 27, 722–733.

[B39] Jansen R. K. Ruhlman T. A. (2012). “Plastid genomes of seed plants,” in Genomics of Chloroplasts and Mitochondria. Eds. Bock R. Knoop V. (Dordrecht: Springer), 103–126.

[B40] Jansen R. K. Cai Z. Raubeson L. A. Daniell H. Depamphilis C. W. Leebens-Mack J.. (2007). Analysis of 81 genes from 64 plastid genomes resolves relationships in angiosperms and identifies genome-scale evolutionary patterns. Proc. Natl. Acad. Sci. U. S. A. 104, 19369–19374. 10.1073/pnas.0709121104 18048330 PMC2148296

[B41] Jansen R. K. Wojciechowski M. F. Sanniyasi E. Lee S. B. Daniell H. (2008). Complete plastid genome sequence of the chickpea (*Cicer arietinum*) and the phylogenetic distribution of *rps12* and *clpP* intron losses among legumes (Leguminosae). Mol. Phylogenet. Evol. 48, 1204–1217. 10.1016/j.ympev.2008.06.013 18638561 PMC2586962

[B42] Jin J.-J. Yu W.-B. Yang J.-B. Song Y. dePamphilis C.W. Yi T.-S. Li D.-Z. (2018). GetOrganelle: a fast and versatile toolkit for accurate *de novo* assembly of oraganelle genomes. bioRxiv, 256479. 10.1101/256479 PMC7488116 32912315

[B43] Kaila T. Chaduvla P. K. Saxena S. Bahadur K. Gahukar S. J. Chaudhury A.. (2016). Chloroplast Genome Sequence of Pigeonpea (*Cajanus cajan* (L.) Millspaugh) and *Cajanus scarabaeoides* (L.) Thouars: Genome organization and comparison with other legumes. Front. Plant Sci. 7, 1847. 10.3389/fpls.2016.01847 28018385 PMC5145887

[B44] Kaila T. Chaduvla P. K. Rawal H. C. Saxena S. Tyagi A. Mithra S. V. A.. (2017). Chloroplast genome sequence of cluster bean (*Cyamopsis tetragonoloba* L.): Genome structure and comparative analysis. Genes 8, E212. 10.3390/genes8090212 28925932 PMC5615346

[B45] Kajita T. Ohashi H. Tateishi. Y. Bailey C. D. Doyle J. J. (2001). *rbcL* and legume phylogeny, with particular reference to Phaseoleae, Millettieae, and allies. Syst. Bot. 26, 15–536. 10.1043/0363-6445-26.3.515

[B46] Kato T. Kaneko T. Sato S. Nakamura Y. Tabata S. (2000). Complete structure of the chloroplast genome of a legume, *Lotus japonicus* . DNA Res. 7, 323–330. 10.1093/dnares/7.6.323 11214967

[B47] Katoh K. Standley D. M. (2013). MAFFT multiple sequence alignment software version 7: improvements in performance and usability. Mol. Biol. Evol. 30, 772–780. 10.1093/molbev/mst010 23329690 PMC3603318

[B48] Kearse M. Moir R. Wilson A. Stones-Havas S. Cheung M. Sturrock S.. (2012). Geneious Basic: an integrated and extendable desktop software platform for the organization and analysis of sequence data. Bioinformatics 28, 1647–1649. 10.1093/bioinformatics/bts199 22543367 PMC3371832

[B49] Keller J. Rousseau-Gueutin M. Martin G. E. Morice J. Boutte J. Coissac E.. (2017). The evolutionary fate of the chloroplast and nuclear *rps16* genes as revealed through the sequencing and comparative analyses of four novel legume chloroplast genomes from *Lupinus* . DNA Res. 24, 343–358. 10.1093/dnares/dsx006 28338826 PMC5737547

[B50] Kim Y. Cullis C. (2017). A novel inversion in the chloroplast genome of marama (*Tylosema esculentum*). J. Exp. Bot. 68, 2065–2072. 10.1093/jxb/erw500 28158587 PMC5429017

[B51] Kim K. J. Jansen R. K. (2005). Two chloroplast DNA inversions originated simultaneously during the early evolution of the sunflower family (Asteraceae). Mol. Biol. Evol. 22, 1783–1792. 10.1093/jxb/erw500 15917497

[B52] Kim H. T. Kim J. S. Moore M. J. Neubig K. M. Williams N. H. Whitten W. M.. (2015). Seven new complete plastome sequences reveal rampant independent loss of the *ndh* gene family across orchids and associated instability of the inverted repeat/small single-copy region boundaries. PloS One 10, e0142215. 10.1371/journal.pone.0142215 26558895 PMC4641739

[B53] Lackey J. A. Polhill R. M. Raven P. H. (1981). “Phaseoleae,” in Advances in Legume Systematics, part 1 (UK: Royal Botanic Gardens, Kew), 301– 327.

[B54] Lanfear R. Frandsen P. B. Wright A. M. Senfeld T. Calcott B. (2017). PartitionFinder 2: new methods for selecting partitioned models of evolution for molecular and morphological phylogenetic analyses. Mol. Biol. Evol. 34, 772–773. 10.1093/molbev/msw260 28013191 10.1093/molbev/msw260

[B55] Langmead B. Salzberg S. L. (2012). Fast gapped-read alignment with Bowtie 2. Nat. Methods 9, 357–359. 10.1038/nmeth.1923 22388286 PMC3322381

[B56] Lavin M. Doyle J. J. Palmer J. D. (1990). Evolutionary significance of the loss of the Chloroplast-DNA inverted repeat in the Leguminosae subfamily Papilionoideae. Evolution 44, 390–402. 10.2307/2409416 28564377

[B57] Lee J. Hymowitz T. (2001). A molecular phylogenetic study of the subtribe Glycininae (Leguminosae) derived from the chloroplast DNA *rps16* intron sequences. Am. J. Bot. 88, 2064–2073. 10.2307/3558432 21669638

[B58] Lee H. L. Jansen R. K. Chumley T. W. Kim K. J. (2007). Gene relocations within chloroplast genomes of *Jasminum* and *Menodora* (Oleaceae) are due to multiple, overlapping inversions. Mol. Biol. Evol. 24, 1161–1180. 10.1093/molbev/msm036 17329229

[B59] Li C. H. Lu G. Q. Orti G. (2008). Optimal data partitioning and a test case for ray-finned fishes (Actinopterygii) based on ten nuclear loci. Syst. Biol. 57, 519–539. 10.1080/10635150802206883 18622808

[B60] Li R. Ma P. F. Wen J. Yi T. S. (2013). Complete sequencing of five araliaceae chloroplast genomes and the phylogenetic implications. PloS One 8, e78568. 10.1371/journal.pone.0078568 24205264 PMC3799623

[B61] Lin C. S. Chen J. J. W. Huang Y. T. Chan M. T. Daniell H. Chang W. J.. (2015). The location and translocation of *ndh* genes of chloroplast origin in the Orchidaceae family. Sci. Rep. 5, 1–10. 10.1038/srep09040 PMC4356964 25761566

[B62] Logacheva M. D. Schelkunov M. I. Nuraliev M. S. Samigullin T. H. Penin A. A. (2014). The plastid genome of mycoheterotrophic monocot *Petrosavia stellaris* exhibits both gene losses and multiple rearrangements. Genome Biol. Evol. 6, 238–246. 10.1093/gbe/evu001 24398375 PMC3914687

[B63] Lohse M. Drechsel O. Kahlau S. Bock R. (2013). OrganellarGenomeDRAW - a suite of tools for generating physical maps of plastid and mitochondrial genomes and visualizing expression data sets. Nucleic Acids Res. 41, W575–W581. 10.1093/nar/gkt289 23609545 PMC3692101

[B64] LPWG (2013). Legume phylogeny and classification in the 21st century: progress, prospects and lessons for other species-rich clades. Taxon 62, 217–248. 10.5167/uzh-78167

[B65] LPWG (2017). A new subfamily classification of the Leguminosae based on a taxonomically comprehensive phylogeny. Taxon 66, 44–77. 10.12705/661.3

[B66] Luo Y. Ma P. F. Li H. T. Yang J. B. Wang H. Li D. Z. (2016). Plastid phylogenomic analyses resolve Tofieldiaceae as the root of the early diverging monocot Order Alismatales. Genome Biol. Evol. 8, 932–945. 10.1093/gbe/evv260 26957030 PMC4823975

[B67] Ma P. F. Zhang Y. X. Zeng C. X. Guo Z. H. Li D. Z. (2014). Chloroplast phylogenomic analyses resolve deep-level relationships of an intractable bamboo tribe Arundinarieae (Poaceae). Syst. Biol. 63, 933–950. 10.1093/sysbio/syu054 25092479

[B68] Magee A. M. Aspinall S. Rice D. W. Cusack B. P. Sémon M. Perry A. S.. (2010). Localized hypermutation and associated gene losses in legume chloroplast genomes. Genome Res. 20, 1700–1710. 10.1101/gr.111955.110 20978141 PMC2989996

[B69] Martin G. E. Rousseau-Gueutin M. Cordonnier S. Lima O. Michon-Coudouel S. Naquin D.. (2014). The first complete chloroplast genome of the Genistoid legume *Lupinus luteus*: evidence for a novel major lineage-specific rearrangement and new insights regarding plastome evolution in the legume family. Ann. Bot. 113, 1197–1210. 10.1093/aob/mcu050 24769537 PMC4030815

[B70] Millen R. S. Olmstead R. G. Adams K. L. Palmer J. D. Lao N. T. Heggie L.. (2001). Many parallel losses of *infA* from chloroplast DNA during angiosperm evolution with multiple independent transfers to the nucleus. Plant Cell 13, 645–658. 10.1105/tpc.13.3.645 11251102 PMC135507

[B71] Mower J. P. Ma P. F. Grewe F. Taylor A. Michael T. P. VanBuren R.. (2019). Lycophyte plastid genomics: extreme variation in GC, gene and intron content and multiple inversions between a direct and inverted orientation of the rRNA repeat. New Phytol. 222, 1061–1075. 10.1111/nph.15650 30556907 PMC6590440

[B72] Naumann J. Der J. P. Wafula E. K. Jones S. S. Wagner S. T. Honaas L. A.. (2016). Detecting and characterizing the highly divergent plastid genome of the nonphotosynthetic parasitic plant *Hydnora visserim* (Hydnoraceae). Genome Biol. Evol. 8, 345–363. 10.1093/gbe/evv256 26739167 PMC4779604

[B73] Nguyen L. T. Schmidt H. A. von Haeseler A. Minh B. Q. (2015). IQTREE: A fast and effective stochastic algorithm for estimating maximum likelihood phylogenies. Mol. Biol. Evol. 32, 268–274. 10.1093/molbev/msu300 25371430 PMC4271533

[B74] Patel R. K. Jain M. (2012). NGS QC toolkit: a toolkit for quality control of next generation sequencing data. PloS One 7, e30619. 10.1371/journal.pone.0030619 22312429 PMC3270013

[B75] Pennington R. T. Lavin M. Ireland H. Klitgaard B. B. Preston J. (2001). Phylogenetic relationships of basal papilionoid legumes based upon sequences of the chloroplast *trnL* intron. Syst. Bot. 26, 537–566. Retrieved from http://www.jstor.org/stable/3093980.

[B76] Perry A. S. Brennan S. Murphy D. J. Wolfe K. H. (2002). Evolutionary re-organization of a large operon in Adzuki bean chloroplast DNA caused by inverted repeat movement. DNA Res. 9, 157–162. 10.1093/dnares/9.5.157 12465715

[B77] Qu X. J. Fan S. J. Wicke S. Yi T. S. (2019). Plastome reduction in the only parasitic gymnosperm Parasitaxus is due to losses of photosynthesis but not housekeeping genes and apparently involves the secondary gain of a large inverted repeat. Genome Biol. Evol. 11, 2789–2796. 10.1093/gbe/evz187 31504501 PMC6786476

[B78] Rambaut A. (2009). FigTree version 1.3.1 [computer program] http://tree.bio.ed.ac.uk.

[B79] Rambaut A. Drummond A. J. (2004). Tracer version 1.5 [computer program] http://beast.bio.ed.ac.uk

[B80] Raubeson L. A. Jansen R. K. (2005). “Chloroplast Genomes of Plants,” in Plant Diversity and Evolution: Genotypic and Phenotypic Variation in Higher Plants. Ed. Henry R. J. (Cambridge, MA: CABI Press), 45–68.

[B81] Rogalski M. Ruf S. Bock R. (2006). Tobacco plastid ribosomal protein S18 is essential for cell survival. Nucleic Acids Res. 34, 4537–4545. 10.1093/nar/gkl634 16945948 PMC1636375

[B82] Ronquist F. Huelsenbeck J. P. (2003). MrBayes 3: Bayesian phylogenetic inference under mixed models. Bioinformatics 19, 1572–1574. 10.1093/bioinformatics/btg180 12912839

[B83] Saarela J. M. Burke S. V. Wysocki W. P. Barrett M. D. Clark L. G. Craine J. M. (2018). A 250 plastome phylogeny of the grass family (Poaceae): topological support under different data partitions. PeerJ 6, e4299. 10.7717/peerj.4299 29416954 PMC5798404

[B84] Sabir J. Schwarz E. Ellison N. Zhang J. Baeshen N. A. Mutwakil M.. (2014). Evolutionary and biotechnology implications of plastid genome variation in the inverted-repeat-lacking clade of legumes. Plant Biotechnol. J. 12, 743–754. 10.1111/pbi.12179 24618204

[B85] Saski C. Lee S. B. Daniell H. Wood T. C. Tomkins J. Kim H. G.. (2005). Complete chloroplast genome sequence of *Glycine max* and comparative analyses with other legume genomes. Plant Mol. Biol. 59, 309–322. 10.1007/s11103-005-8882-0 16247559

[B86] Schattner P. Brooks A. N. Lowe T. M. (2005). The tRNAscan-SE, snoscan and snoGPS web servers for the detection of tRNAs and snoRNAs. Nucleic Acids Res. 33, W686–W689. 10.1093/nar/gki366 15980563 PMC1160127

[B87] Schrire B. D. Lavin M. Barker N. P. Forest F. (2009). Phylogeny of the tribe Indigofereae (Leguminosae- Papilionoideae): geographically structured more in succulent-rich and temperate settings than in grass-rich environments. Am. J. Bot. 96, 816–852. 10.3732/ajb.0800185 21628237

[B88] Schrire B. D. (2005a). “Indigofereae,” in Legumes of the world. Ed. Lewis (Kew, UK: Royal Botanic Gardens), 361–365.

[B89] Schrire B. D. (2005b). “Millettieae,” in Legumes of the world. Ed. Lewis (Kew, UK: Royal Botanic Gardens), 367–387.

[B90] Schrire B. D. (2005c). “Abreae,” in Legumes of the world. Ed. Lewis (Kew, UK: Royal Botanic Gardens), 389–391.

[B91] Schrire B. D. (2005d). “Phaseoleae,” in Legumes of the world. Ed. Lewis (Kew, UK: Royal Botanic Gardens), 393–431.

[B92] Schwarz E. N. Ruhlman T. A. Sabir J. S. M. Hajrah N. H. Alharbi N. S. Al-Malki A. L.. (2015). Plastid genome sequences of legumes reveal parallel inversions and multiple losses of *rps16* in papilionoids. J. Syst. Evol. 53, 458–468. 10.1111/jse.12179

[B93] Sharp P. A. (1985). On the origin of RNA splicing and introns. Cell 42, 397–400. 10.1016/0092-8674(85)90092-3 2411416

[B94] Simpson B. B. Ogorzaly M. C. (2001). Economic botany: plants in our world. 3rd ed. (New York: McGraw-Hill, Inc.), 544.

[B95] Smith A. G. Wilson R. M. Kaethner T. M. Willey D. L. Gray J. C. (1991). Pea chloroplast genes encoding a 4 kDa polypeptide of photosystem I and a putative enzyme of C1 metabolism. Curr. Genet. 19:403–10. 10.1007/bf00309603 1913879

[B96] Stefanovic S. Pfeil B. E. Palmer J. D. Doyle J. J. (2009). Relationships among phaseoloid legumes based on sequences from eight chloroplast regions. Syst. Bot. 34, 115–128. 10.1600/036364409787602221

[B97] Ueda M. Fujimoto M. Takanashi H. Arimura S. I. Tsutsumi N. Kadowaki K. I. (2008). Substitution of the gene for chloroplast *rps16 was* assisted by generation of dual targeting signal. Mol. Biol. Evol. 25, 1566–1575. 10.1093/molbev/msn102 18453549

[B98] Vatanparast M. Powell A. Doyle J. J. Egan A. N. (2018). Targeting legume loci: a comparison of three methods for target enrichment bait design in Leguminosae phylogenomics. Appl. Plant Sci. 6, e1036. 10.1002/aps3.1036 29732266 PMC5895186

[B99] Walker J. F. Zanis M. J. Emery N. C. (2014). Comparative analysis of complete chloroplast genome sequence and inversion variation in *Lasthenia burkei* (Madieae, Asteraceae). Am. J. Bot. 101, 722–729. 10.3732/ajb.1400049 24699541

[B100] Wang R. J. Cheng C. L. Chang C. C. Wu C. L. Su T. M. Chaw S. M. (2008). Dynamics and evolution of the inverted repeat-large single copy junctions in the chloroplast genomes of monocots. BMC Evol. Biol. 8, 36. 10.1186/1471-2148-8-36 18237435 PMC2275221

[B101] Wang Y. H. Qu X. J. Chen S. Y. Li D. Z. Yi T. S. (2017). Plastomes of Mimosoideae: structural and size variation, sequence divergence, and phylogenetic implication. Tree Genet. Genomes 13, 41. 10.1007/s11295-017-1124-1

[B102] Wang Y. H. Wicke S. Wang H. Jin J. J. Chen S. Y. Zhang S. D.. (2018). Plastid genome evolution in the early-diverging legume subfamily Cercidoideae (Fabaceae). Front. Plant Sci. 9, 138. 10.3389/fpls.2018.00138 29479365 PMC5812350

[B103] Weng M. L. Blazier J. C. Govindu M. Jansen R. K. (2014). Reconstruction of the ancestral plastid genome in Geraniaceae reveals a correlation between genome rearrangements, repeats, and nucleotide substitution rates. Mol. Biol. Evol. 31, 645–659. 10.1093/molbev/mst257 24336877

[B104] Wick R. R. Schultz M. B. Zobel J. Holt K. E. (2015). Bandage: interactive visualization of *de novo* genome assemblies. Bioinformatics 31, 3350–3352. 10.1093/bioinformatics/btv383 26099265 PMC4595904

[B105] Williams A. V. Boykin L. M. Howell K. A. Nevill P. G. Small L. (2015). The complete sequence of the *Acacia ligulata* chloroplast genome reveals a highly divergent *clpP1* gene. PloS One 10, e0138367. 10.1371/journal.pone.0125768 25955637 PMC4425659

[B106] Wojciechowski M. F. Lavin M. Sanderson M. J. (2004). A phylogeny of legumes (Leguminosae) based on analysis of the plastid *matK* gene resolves many well-supported subclades within the family. Am. J. Bot. 91, 1846–1862. 10.3732/ajb.91.11.1846 21652332

[B107] Wojciechowski M. F. (2003). “Reconstructing the phylogeny of legumes (Leguminosae): An early 21st century perspective,” in Advances in Legume Systematics 10. Eds. Klitgaard B. B. Bruneau A. (Kew: Royal Botanic Gardens), 5–35.

[B108] Wu C. S. Chaw S. M. (2016). Large-Scale comparative analysis reveals the mechanisms driving plastomic compaction, reduction, and inversions in Conifers II (Cupressophytes). Genome Biol. Evol. 8, 740–3750. 10.1093/gbe/evw278 PMC5491842 28039231

[B109] Wu C. S. Lai Y. T. Lin C. P. Wang Y. N. Chaw S. M. (2009). Evolution of reduced and compact chloroplast genomes (cpDNAs) in gnetophytes: selection toward a lower-cost strategy. Mol. Phylogenet. Evol. 52, 115–124. 10.1016/j.ympev.2008.12.026 19166950

[B110] Wyman S. K. Jansen R. K. Boore J. L. (2004). Automatic annotation of organellar genomes with DOGMA. Bioinformatics 20, 3252–3255. 10.1093/bioinformatics/bth352 15180927

[B111] Xu J. Feng D. Song G. Wei X. Chen L. Wu X.. (2003). The first intron of rice EPSP synthase enhances expression of foreign gene. Sci. China Ser. C. Life. Sci. 46, 561–569. 10.1360/02yc012 18758713

[B112] Zeng S. Zhou T. Han K. Yang Y. Zhao J. Liu Z. L. (2017). The complete chloroplast genome sequences of six *rehmannia* species. Genes 8, 103. 10.3390/genes8030103 PMC5368707 28294981

[B113] Zeng C. Hollingsworth P. M. Yang J. He Z. S. Zhang Z. R. Li D. Z.. (2018). Genome skimming herbarium specimens for DNA barcoding and phylogenomics. Plant Methods 14, 1–14. 10.1186/s13007-018-0300-0 29928291 PMC5987614

[B114] Zhang S. D. Jin J. J. Chen S. Y. Chase M. W. Soltis D. E. Li H. T.. (2017). Diversification of rosaceae since the late cretaceous based on plastid phylogenomics. New Phytol. 214, 1355–1367. 10.1111/nph.14461 28186635

[B115] Zhang H. Jin J. Moore M. J. Yi T. S. Li D. (2018). Plastome characteristics of Cannabaceae. Plant Divers. 40, 127–137. 10.1016/j.pld.2018.04.003 30175293 PMC6114266

[B116] Zhang R. Wang Y. -H. Jin J. -J. Stull G. W. Bruneau A. Cordoso D.. (2020). Exploration of plastid phylogenomic conflict yields new insights into the deep relationships of Leguminosae. Syst. Biol. 10.1093/sysbio/syaa013 PMC7302050 32065640

