# Corrigendum: New Insights Into the Plastome Evolution of the Millettioid/Phaseoloid Clade (Papilionoideae, Leguminosae)

**DOI:** 10.3389/fpls.2021.652483

**Published:** 2021-03-01

**Authors:** Oyetola Oyebanji, Rong Zhang, Si-Yun Chen, Ting-Shuang Yi

**Affiliations:** ^1^Germplasm Bank of Wild Species, Kunming Institute of Botany, Chinese Academy of Sciences, Kunming, China; ^2^Kunming College of Life Science, University of Chinese Academy of Sciences, Beijing, China

**Keywords:** evolutionary relationships, inversion, IR expansion/contraction, Leguminosae, Plastome, the Millettioid/Phaseoloid clade

In the original article, there was a mistake in the legend for Table 1 as published. The use of “*C. gracilis*” in the ^h^ legend is incorrect. The correct legend appears below.

“^h^Duplicated in the IR of all species except *D. araripensis, L. domingensis, O. pinnata, P. violacea, X. stuhlmannii, I. linifolia and tinctoria*.”

In the original article, there was a mistake in Figure 1 and **4** as published. **The mistakes were:** ***Cochlianthus gracilis* (Phaseoleae)**, ***Craspedolobium schochii*** **(Millettieae), and** ***Shuteria vestita* (Desmodieae)**. The corrected taxonomic names appear below.

**Figure 1:**
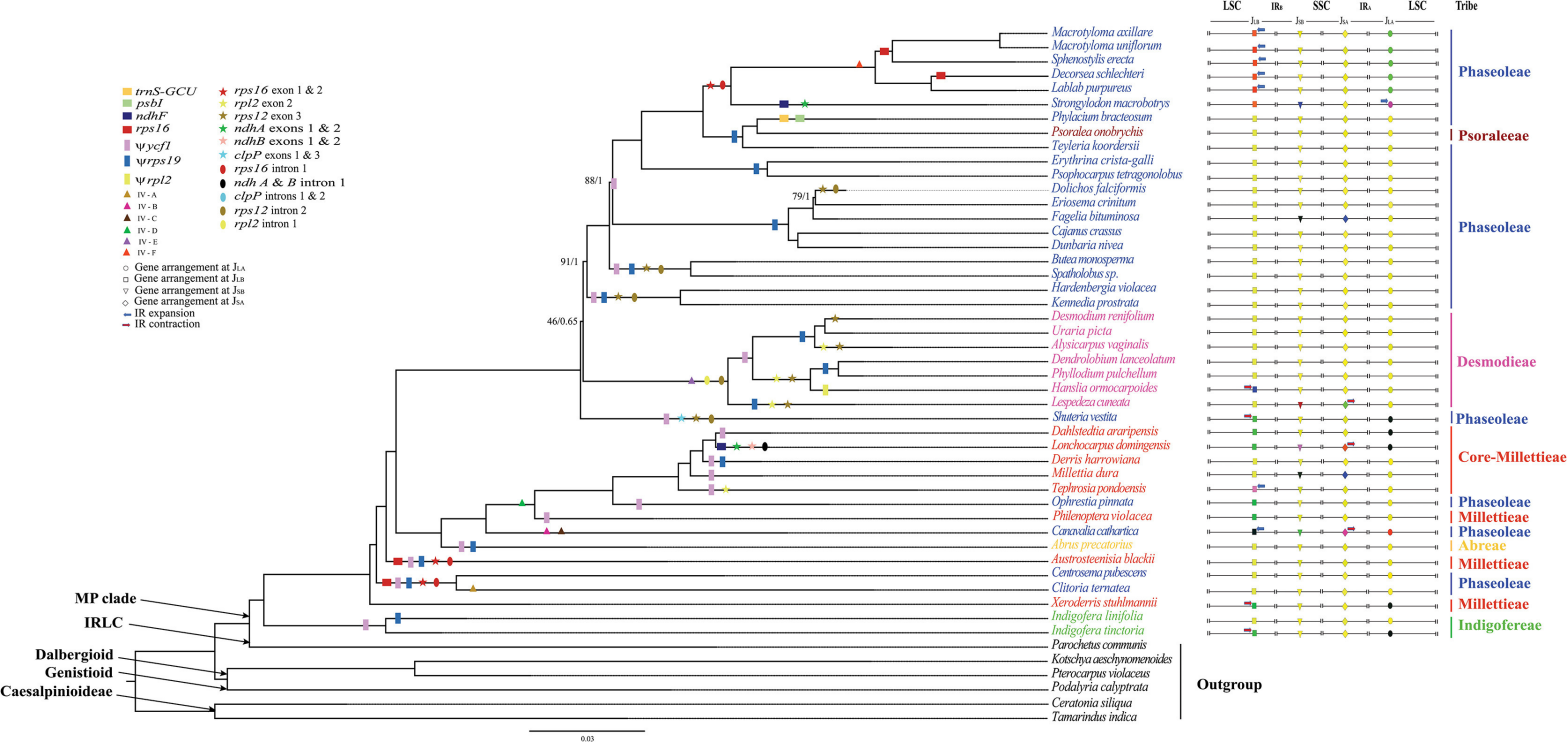
The ML tree of the MP clade reconstructed based on the CP and the variation of IR/SC junctions. Numbers at nodes correspond to ML bootstrap percentages (only values <100% are shown) and Bayesian inference (BI) posterior probabilities (only probabilities <1.0 are shown). Genes loss, pseudogenes, inversions (IV), exon and intron loss, in the plastome, are indicated on the branches using coloured squares, rectangles, triangles, stars and oval shapes, respectively. The IR expansion and contraction are shown by blue and red arrow, respectively.

**Inserted CORRECTED names:** *Philenoptera violacea (*Millettieae), *Spatholobus* sp. (Phaseoleae), and *Shuteria vestita* (Phaseoleae), respectively.

In the original article, there was a mistake in **Supplementary Figure S1** as published. T**he mistakes were:** ***Cochlianthus gracilis* and** ***Craspedolobium schochii***.

**Inserted CORRECTED names:** *Philenoptera violacea* and *Spatholobus* sp. respectively.

In the original article, there was a mistake in **Supplementary Figure S2** as published. T**he mistakes were:** ***Cochlianthus gracilis* and** ***Craspedolobium schochii***.

**Inserted CORRECTED names:** *Philenoptera violacea* and *Spatholobus* sp., respectively.

In the original article, there was a mistake in **Supplementary Figure S3** as published. T**he mistakes were:** ***Cochlianthus gracilis* and** ***Craspedolobium schochii***.

**Inserted CORRECTED names:** *Philenoptera violacea* and *Spatholobus* sp., respectively.

In the original article, there was a mistake in **Supplementary Table S1** as published. **The mistakes were:** ***Cochlianthus gracilis* and** ***Craspedolobium schochii***.

**Inserted CORRECTED names:** *Philenoptera violacea* and *Spatholobus* sp. have been inserted to replace the initial names respectively.

In the original article, there was a mistake in **Supplementary Table S2** as published. **The mistakes were:** ***Cochlianthus gracilis* (Phaseoleae)**, ***Craspedolobium schochii*** **(Millettieae), and** ***Shuteria vestita*** **(Desmodieae)**.

**Inserted CORRECTED names**: *Philenoptera violacea (*Millettieae), *Spatholobus* sp. (Phaseoleae), and *Shuteria vestita* (Phaseoleae), respectively.

In the original article, there was an error: The mean plastome coverage ranged between 162.0 × (*Cochlianthus gracilis* Benth., Phaseoleae) and 1,536.4 × [*Cajanus crassus* (Prain ex King) Maesen, Phaseoleae]. A correction has been made to ***Section: Results, Sub-section-* Plastome Organization and Size**.

**Inserted CORRECTED paragraph:** The mean plastome coverage ranged between 162.0 × (*Philenoptera violacea* (Klotzsch) Schrire, Millettieae) and 1,536.4 × [*Cajanus crassus* (Prain ex King) Maesen, Phaseoleae].

In the original article, there was an error: ***C. gracilis***. A correction has been made to ***Section: Results***, ***Sub-section* Plastome Structural Variations in the MP Clade**.

**Inserted CORRECTED paragraph:** *P. violacea*.

In the original article, there was an error: However, the lineage consisting of *Butea monosperma* (Lam.) Kuntze and *Craspedolobium schochii* Harms has different phylogenetic position in trees of CP and NCDs, and that of CDs, but both relationships were weakly supported. Also, the tribe Desmodieae was weakly supported to be monophyletic in CDs, but being weakly supported to be paraphyletic in CP and NCDs. The tribe Indigofereae was strongly supported as sister to the remainder of the MP clade (BS = 100%, and PP = 1.0). Based on the current sampling, it is not sure if the tribe Desmodieae is monophyletic, while the tribes Millettieae and Phaseoleae appear non-monophyletic. correction has been made to ***Section: Results***, ***Sub-section* Phylogenetic Relationships of the MP Clade**.

**Inserted CORRECTED paragraph:** However, the lineage consisting of *Butea monosperma* (Lam.) Kuntze and *Spatholobus* Hassk sp. has different phylogenetic position in trees of CP and NCDs, and that of CDs, but both relationships were weakly supported. Also, the tribe Desmodieae was weakly supported to be monophyletic in CP and NCDs data matrices whereas strongly supported by CDs data. The tribe Indigofereae was strongly supported as sister to the remainder of the MP clade (BS = 100%, and PP = 1.0). Based on the current sampling, it is not sure if the tribe Desmodieae is monophyletic, while the tribes Millettieae and Phaseoleae appear non-monophyletic.

In the original article, there was an error: According to this study, with the exception of the loss of the *clpP* introns 1 and 2 in a single species of *S. vestita* (Desmodieae) and the loss of *ndh A* and *ndh B* intron 1 in a single species of *L. domingensis* (Millettieae), two other introns (*rps16* and *rps12*) have experienced multiple independent loss during the plastome evolution of the species from the MP clade. This finding agrees with the previous studies on the independent loss of *rpsl2, rps16*, and *clpP* introns in the MP clade (Guo et al., 2007; Schwarz et al., 2015; Kaila et al., 2016). A correction has been made to ***Section: Discussion***, ***Sub-section* Evolutionary Pattern of PSV in the MP Clade**.

**Inserted CORRECTED paragraph:** According to this study, with the exception of the loss of the *clpP* introns 1 and 2 in a single species of *S. vestita* (Phaseoleae) and the loss of *ndh A* and *ndh B* intron 1 in a single species of *L. domingensis* (Millettieae), two other introns (*rps16* and *rps12*) have experienced multiple independent loss during the plastome evolution of the species from the MP clade. This finding agrees with the previous studies on the independent loss of *rpsl2, rps16*, and *clpP* introns in the MP clade (Guo et al.,^2007^; Schwarz et al., 2015; Kaila et al., 2016).

In the original article, there was an error: Desmodieae was supported as monophyletic group in previous studies (Bruneau et al., 1994; Doyle et al., 1997; Kajita et al., 2001; Stefanovic et al., 2009; Cardoso et al., 2013; de Queiroz et al., 2015; Egan et al., 2016), however this tribe was weakly supported as monophyletic by CDs but paraphyletic by CP and NCDs (Figure 4). A correction has been made to ***Section:*** ***Discussion***, ***Sub-section* Phylogenetic Relationships in the MP Clade**.

**Figure 4:**
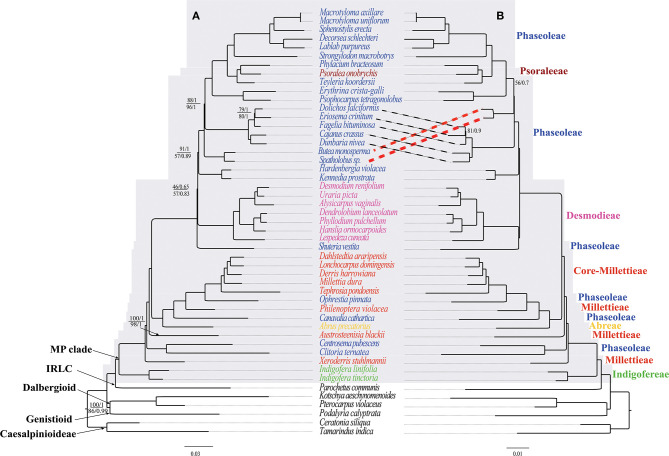
The ML and BI phylogenetic relationships reconstructed for the MP clade. **(A)** CP and NCDs, and **(B)** CDs. Numbers at nodes correspond to ML bootstrap percentages (only values <100% are shown) and Bayesian inference (BI) posterior probabilities (only probabilities <1.0 are shown). For **(A)**, the values above and below the line represents support values for the CP and NCDs, respectively. The thick dotted lines indicate topology differences. The scale bar represents the mean nucleotide substitutions per site along the branch.

**Inserted CORRECTED paragraph:** Desmodieae was supported as monophyletic group in previous studies (Bruneau et al., 1994; Doyle et al., 1997; Kajita et al., 2001; Stefanovic et al., 2009; Cardoso et al., 2013; de Queiroz et al., 2015; Egan et al., 2016), however this tribe was strongly supported as monophyletic by CDs but weakly supported by CP and NCDs (Figure 4).

In the original article, there was an error: Notably, our multi-locus plastome data strongly supported (BS = 100%, PP = 1) the evolutionary position of *S. vestita* within the tribe Desmodieae, in contrast with previous placement in the tribe Phaseoleae (Lackey et al., 1981; de Queiroz et al., 2015). Formerly, the genus *Shuteria* was included in the tribe Phaseoleae based on flower structures shared with core Phaseoleae species (e.g., *Amphicarpaea* Elliott ex Nutt., *Cologania* Kunth, and *Dumasia* DC., Lackey et al., 1981). It is noteworthy that a similar phylogenetic placement in the MP clade has been shown from analysis based on the single plastid region *matK* (de Queiroz et al., 2015). Therefore, our phylogeny supports the placement of *S. vestita* within the tribe Desmodieae. A correction has been made to ***Section: Discussion***, ***Sub-section* Phylogenetic Relationships in the MP Clade**.

**CORRECTED paragraph:** Notably, our multi-locus plastome data suggested (BS = 100%, PP = 1) the evolutionary position of *S. vestita* as sister to the tribe Desmodieae, in contrast with previous placement close to the subtribe Kennediinae of the tribe Phaseoleae (e.g., de Queiroz et al., 2015). Formerly, the genus *Shuteria* was included in the tribe Phaseoleae based on flower structures shared with core Phaseoleae species (e.g., *Amphicarpaea* Elliott ex Nutt., *Cologania* Kunth, and *Dumasia* DC., Lackey et al., 1981). It is noteworthy that a similar phylogenetic placement in the MP clade has been shown from analysis based on the single plastid region *matK* (de Queiroz et al., 2015). Therefore, our phylogeny supports the placement of *S. vestita* as sister to the tribe Desmodieae. Nevertheless, we expect that future phylogenetic studies would improve the understanding of the phylogenetic relationships of the genus *Shuteria* within the clade.

The authors apologize for these errors and state that this does not change the scientific conclusions of the article in any way.

The original article has been updated.
