# Supplementary material for: New Insights Into the Plastome Evolution of the Millettioid/Phaseoloid Clade (Papilionoideae, Leguminosae)
Source: Front Plant Sci. 2020 Mar 10;11:151. doi: 10.3389/fpls.2020.00151 (PMC7076112; doi:10.3389/fpls.2020.00151)
Supplement: Supplementary file 1 [file Presentation_1.zip › Supplementary/Figure S1.PDF]

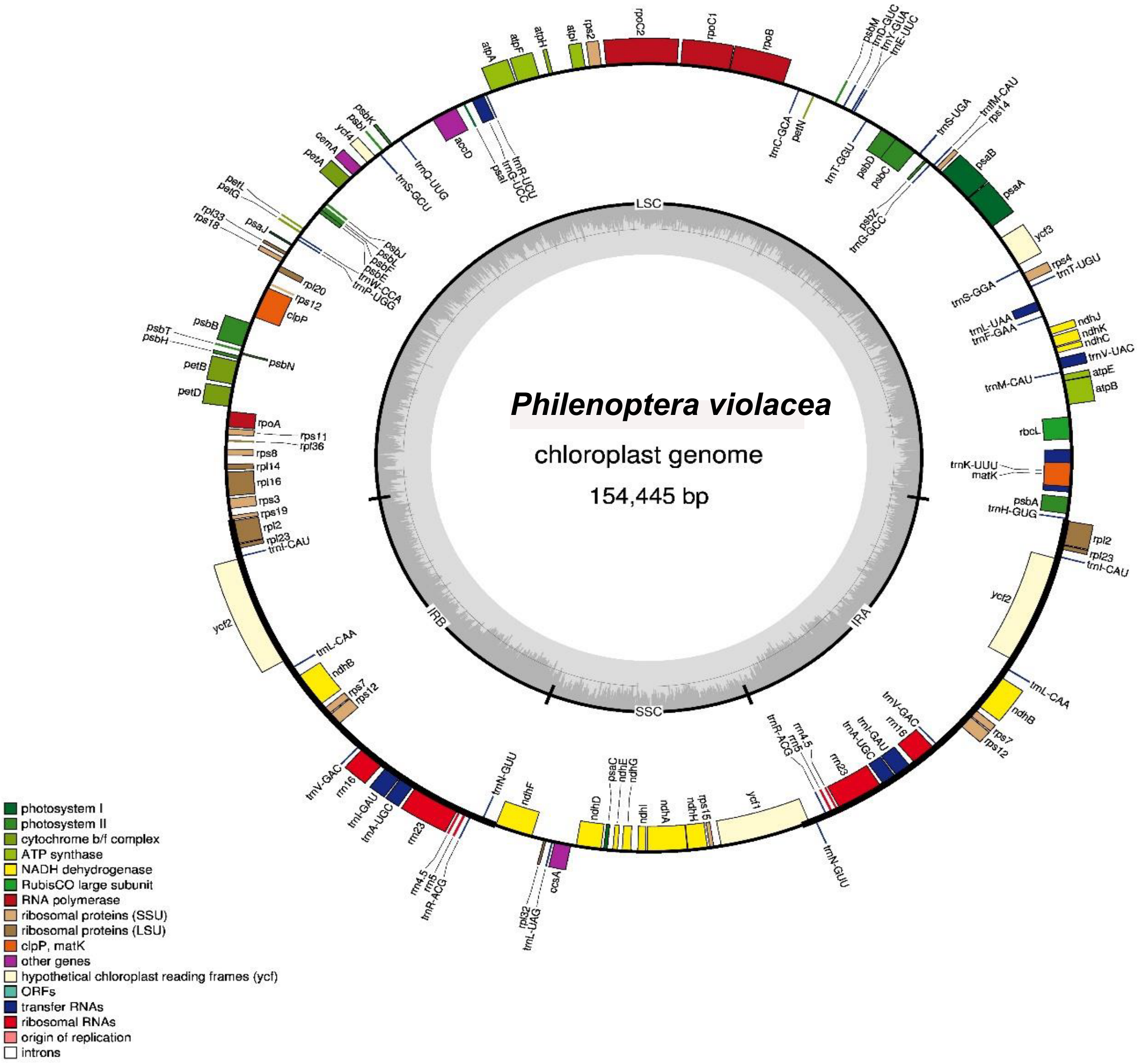
Philenoptera violacea
chloroplast genome
154,445 bp
LSC
IRA
SSC
IRB
photosystem I
photosystem II
cytochrome b/f complex
ATP synthase
NADH dehydrogenase
RubisCO large subunit
RNA polymerase
ribosomal proteins (SSU)
ribosomal proteins (LSU)
clpP, matK
other genes
hypothetical chloroplast reading frames (ycf)
ORFs
transfer RNAs
ribosomal RNAs
origin of replication
introns

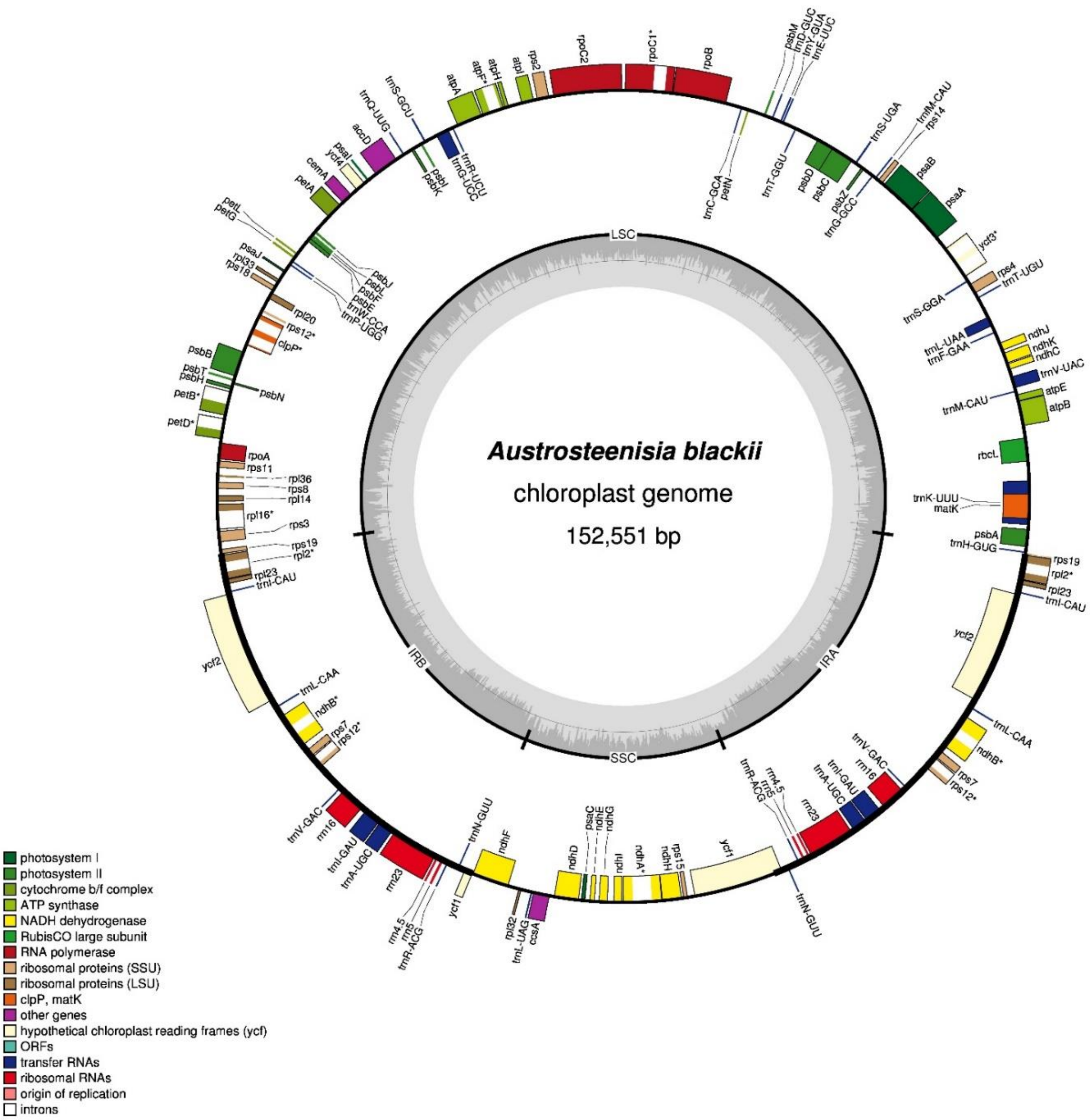

Austrosteenisia blackii
chloroplast genome
152,551 bp
LSC
IRA
SSC
IRB
rpoC2
rpoC1*
rpoB
rps2
atpI
atpH
atpF*
atpA
trnS-GCU
trnQ-UUG
accD
psaI
ycf4
cemA
petA
petL
petG
psaJ
rpl33
rps18
rpl20
rps12*
clpP*
psbB
psbT
psbH
petB*
petD*
psbN
rpoA
rps11
rpl36
rps8
rpl14
rpl16*
rps3
rps19
rpl2*
rpl23
trnI-CAU
ycf2
trnL-CAA
ndhB*
rps7
trnV-GAC
rrn16
trnI-GAU
trnA-UGC
rrn23
rrn4.5
rrn5
trnR-ACG
trnN-GUU
ndhF
ycf1
rpl32
trnL-UAG
ccsA
ndhD
psaC
ndhE
ndhG
ndhI
ndhA*
ndhH
rps15
psbM
trnD-GUC
trnY-GUA
trnE-UUC
trnC-GCA
petN
trnT-GGU
psbD
psbC
psbZ
trnG-GCC
trnS-UGA
trnfM-CAU
rps14
psaB
psaA
ycf3*
rps4
trnT-UGU
trnS-GGA
trnL-UAA
trnF-GAA
ndhJ
ndhK
ndhC
trnV-UAC
atpE
atpB
trnM-CAU
rbcL
trnK-UUU
matK
psbA
trnH-GUG
trnR-UCU
trnG-UCC
psbI
psbK
psbJ
psbL
psbF
psbE
trnW-CCA
trnP-UGG
photosystem I
photosystem II
cytochrome b/f complex
ATP synthase
NADH dehydrogenase
RubisCO large subunit
RNA polymerase
ribosomal proteins (SSU)
ribosomal proteins (LSU)
clpP, matK
other genes
hypothetical chloroplast reading frames (ycf)
ORFs
transfer RNAs
ribosomal RNAs
origin of replication
introns

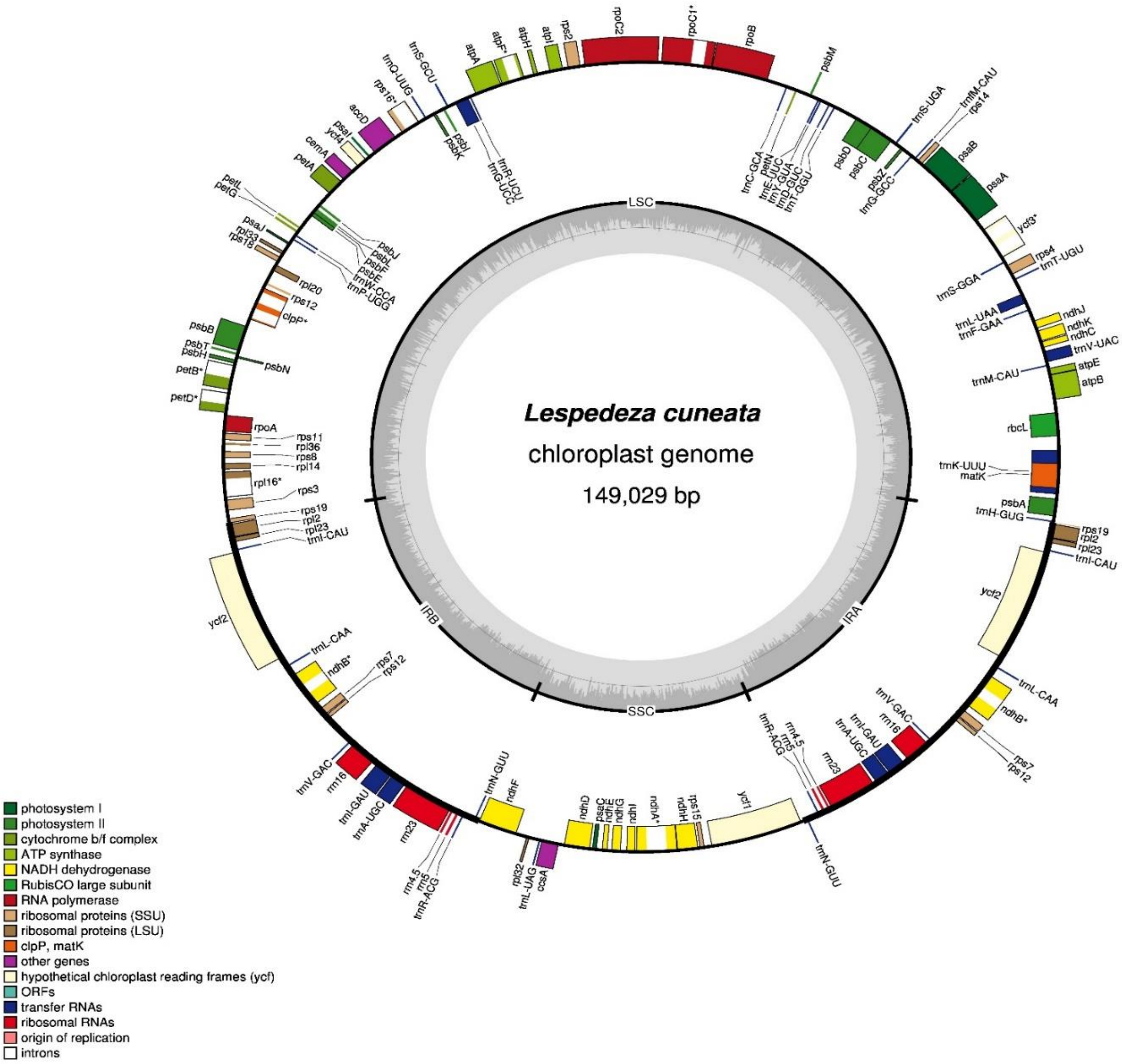
Lespedeza cuneata
chloroplast genome
149,029 bp
LSC
IRA
IRB
SSC
photosystem I
photosystem II
cytochrome b/f complex
ATP synthase
NADH dehydrogenase
RubisCO large subunit
RNA polymerase
ribosomal proteins (SSU)
ribosomal proteins (LSU)
clpP, matK
other genes
hypothetical chloroplast reading frames (ycf)
ORFs
transfer RNAs
ribosomal RNAs
origin of replication
introns

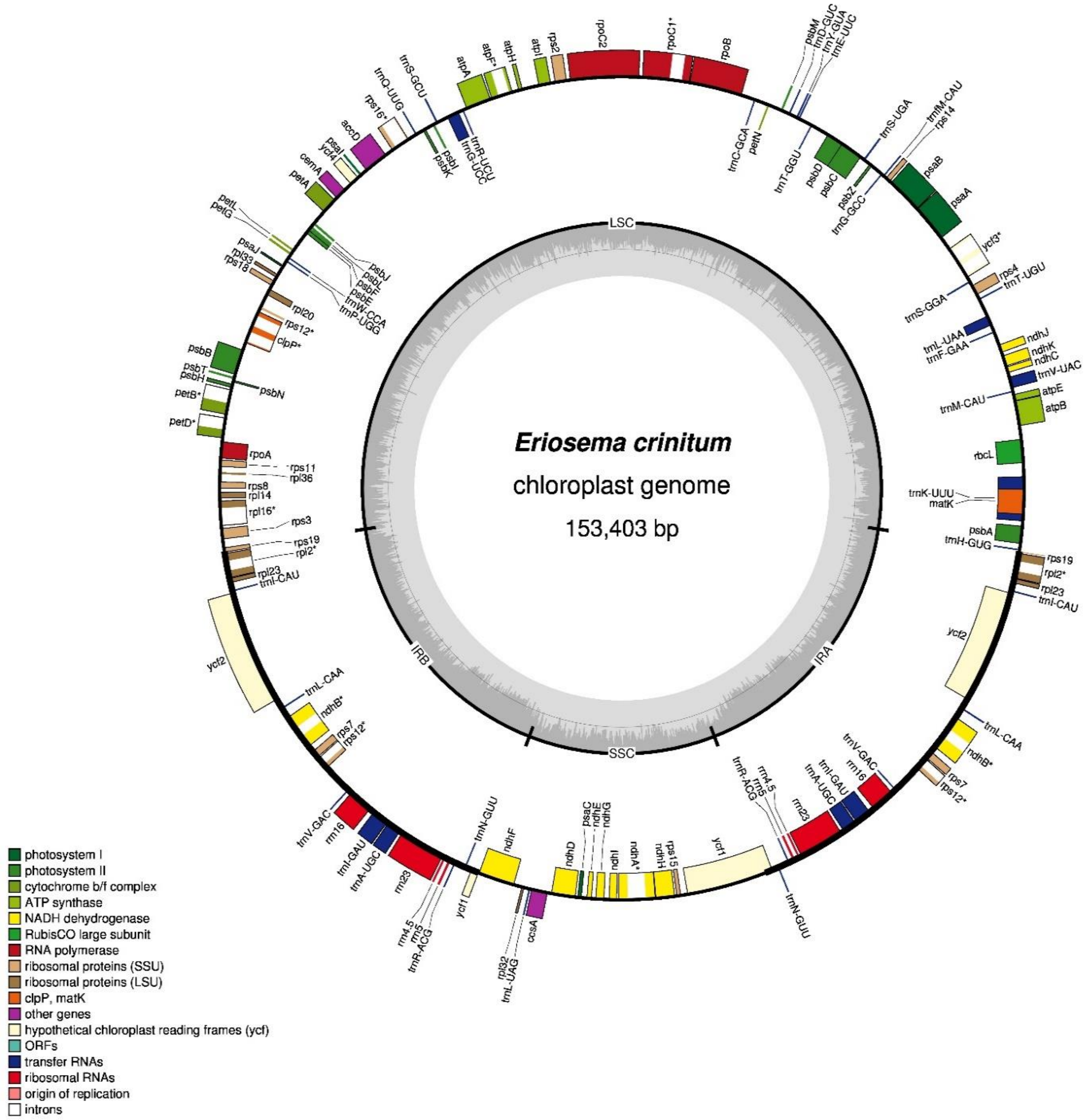
Eriosema crinitum
chloroplast genome
153,403 bp
LSC
IRA
SSC
IRB
photosystem I
photosystem II
cytochrome b/f complex
ATP synthase
NADH dehydrogenase
RubisCO large subunit
RNA polymerase
ribosomal proteins (SSU)
ribosomal proteins (LSU)
clpP, matK
other genes
hypothetical chloroplast reading frames (ycf)
ORFs
transfer RNAs
ribosomal RNAs
origin of replication
introns

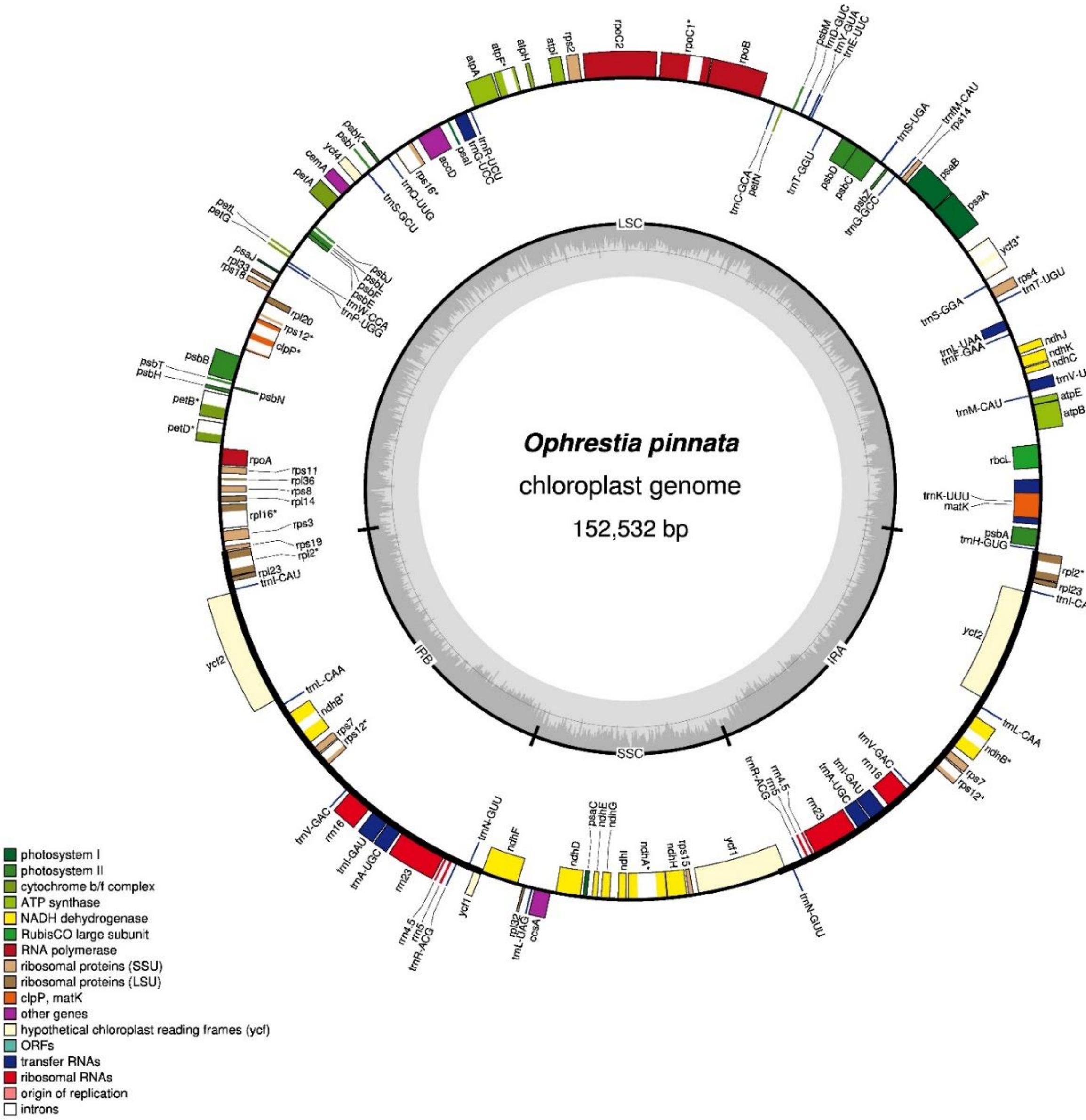

Ophrestia pinnata
chloroplast genome
152,532 bp
LSC
IRA
SSC
IRB
rpoC2
rpoC1*
rpoB
rps2
atpI
atpH
atpF*
atpA
psbM
trnD-GUC
trnY-GUA
trnE-UUC
trnC-GCA
petN
trnT-GGU
psbD
psbC
psbZ
trnG-GCC
trnS-UGA
trnfM-CAU
rps14
psaB
psaA
ycf3*
rps4
trnT-UGU
trnS-GGA
trnL-UAA
trnF-GAA
ndhJ
ndhK
ndhC
trnV-UAC
trnM-CAU
atpE
atpB
rbcL
trnK-UUU
matK
psbA
trnH-GUG
rpl2*
rpl23
trnI-CAU
ycf2
trnL-CAA
ndhB*
rps7
rps12*
trnV-GAC
rrn16
trnI-GAU
trnA-UGC
rrn23
rrn4.5
rrn5
trnR-ACG
trnN-GUU
ycf1
rps15
ndhH
ndhA*
ndhI
ndhG
ndhE
psaC
ndhD
ccsA
trnL-UAG
rpl32
ndhF
rpoA
rps11
rpl36
rps8
rpl14
rpl16*
rps3
rps19
psbB
psbT
psbH
psbN
petB*
petD*
rpl20
rps12*
clpP*
psaJ
rpl33
rps18
petL
petG
psbJ
psbL
psbF
psbE
trnW-CCA
trnP-UGG
petA
cemA
ycf4
psbI
psbK
trnQ-UUG
trnS-GCU
rps16*
accD
psaI
trnG-UCC
trnR-UCU
photosystem I
photosystem II
cytochrome b/f complex
ATP synthase
NADH dehydrogenase
RubisCO large subunit
RNA polymerase
ribosomal proteins (SSU)
ribosomal proteins (LSU)
clpP, matK
other genes
hypothetical chloroplast reading frames (ycf)
ORFs
transfer RNAs
ribosomal RNAs
origin of replication
introns

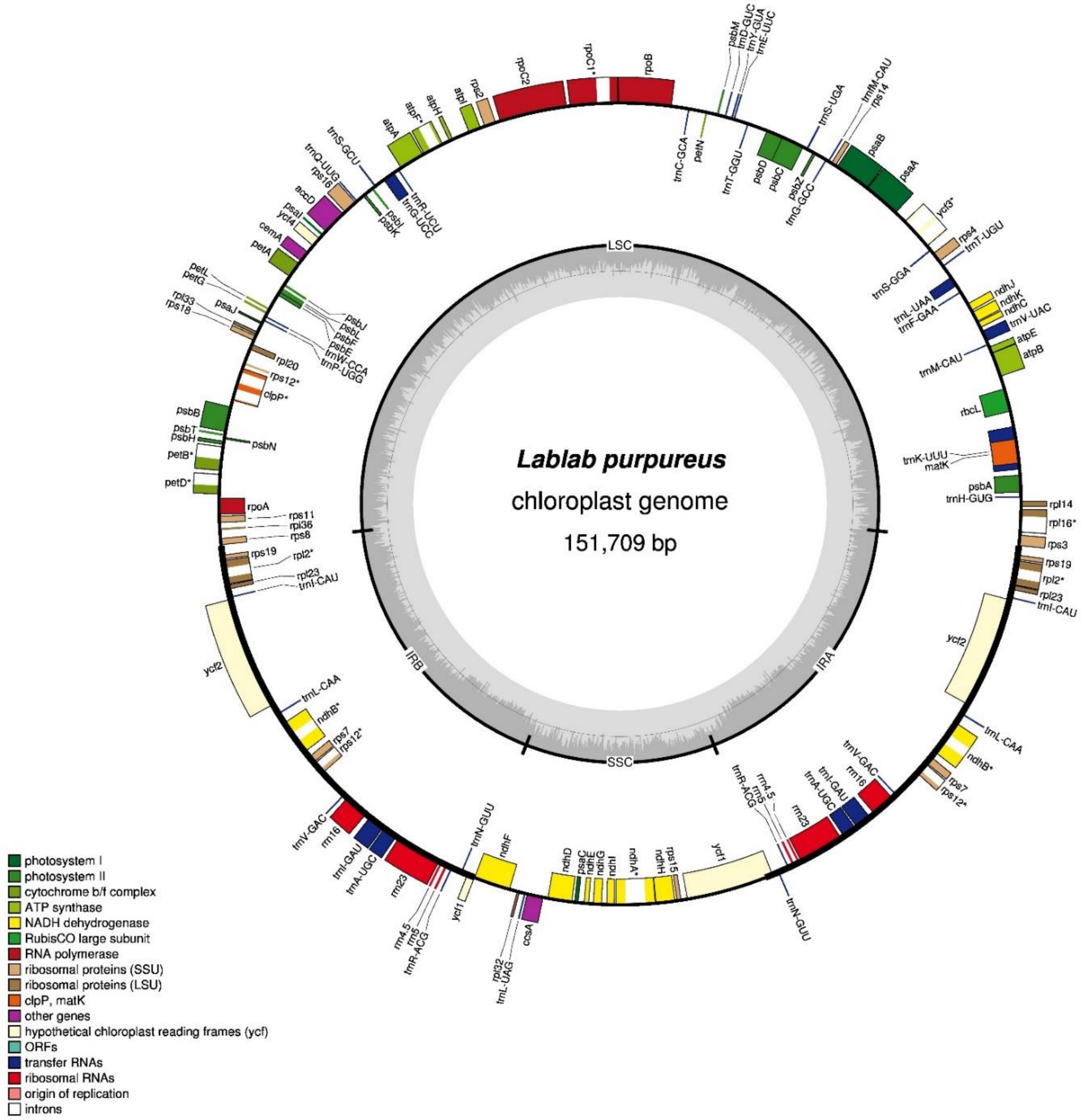

Lablab purpureus
chloroplast genome
151,709 bp
LSC
IRA
SSC
IRB
rpoC2
rpoC1*
rpoB
psbM
trnD-GUC
trnY-GUA
trnE-UUC
trnC-GCA
petN
trnT-GGU
psbD
psbC
psbZ
trnG-GCC
trnS-UGA
trnfM-CAU
rps14
psaB
psaA
ycf3*
rps4
trnT-UGU
trnS-GGA
trnL-UAA
trnF-GAA
ndhJ
ndhK
ndhC
trnV-UAC
atpE
atpB
trnM-CAU
rbcL
trnK-UUU
matK
psbA
trnH-GUG
rpl14
rpl16*
rps3
rps19
rpl2*
rpl23
trnI-CAU
ycf2
trnL-CAA
ndhB*
rps7
rps12*
trnV-GAC
rrn16
trnI-GAU
trnA-UGC
rrn23
rrn4.5
rrn5
trnR-ACG
trnN-GUU
ycf1
rps15
ndhH
ndhA*
ndhI
ndhG
ndhE
psaC
ndhD
ccsA
rpl32
trnL-UAG
ndhF
rpoA
rps11
rpl36
rps8
petD*
petB*
psbH
psbT
psbN
psbB
clpP*
rpl20
rps18
rpl33
psaJ
petG
petL
psbJ
psbL
psbF
psbE
trnW-CCA
trnP-UGG
petA
cemA
ycf4
psaI
accD
rps16
trnQ-UUG
trnS-GCU
psbI
psbK
trnR-UCU
trnG-UCC
atpA
atpF*
atpH
atpI
rps2
photosystem I
photosystem II
cytochrome b/f complex
ATP synthase
NADH dehydrogenase
RubisCO large subunit
RNA polymerase
ribosomal proteins (SSU)
ribosomal proteins (LSU)
clpP, matK
other genes
hypothetical chloroplast reading frames (ycf)
ORFs
transfer RNAs
ribosomal RNAs
origin of replication
introns

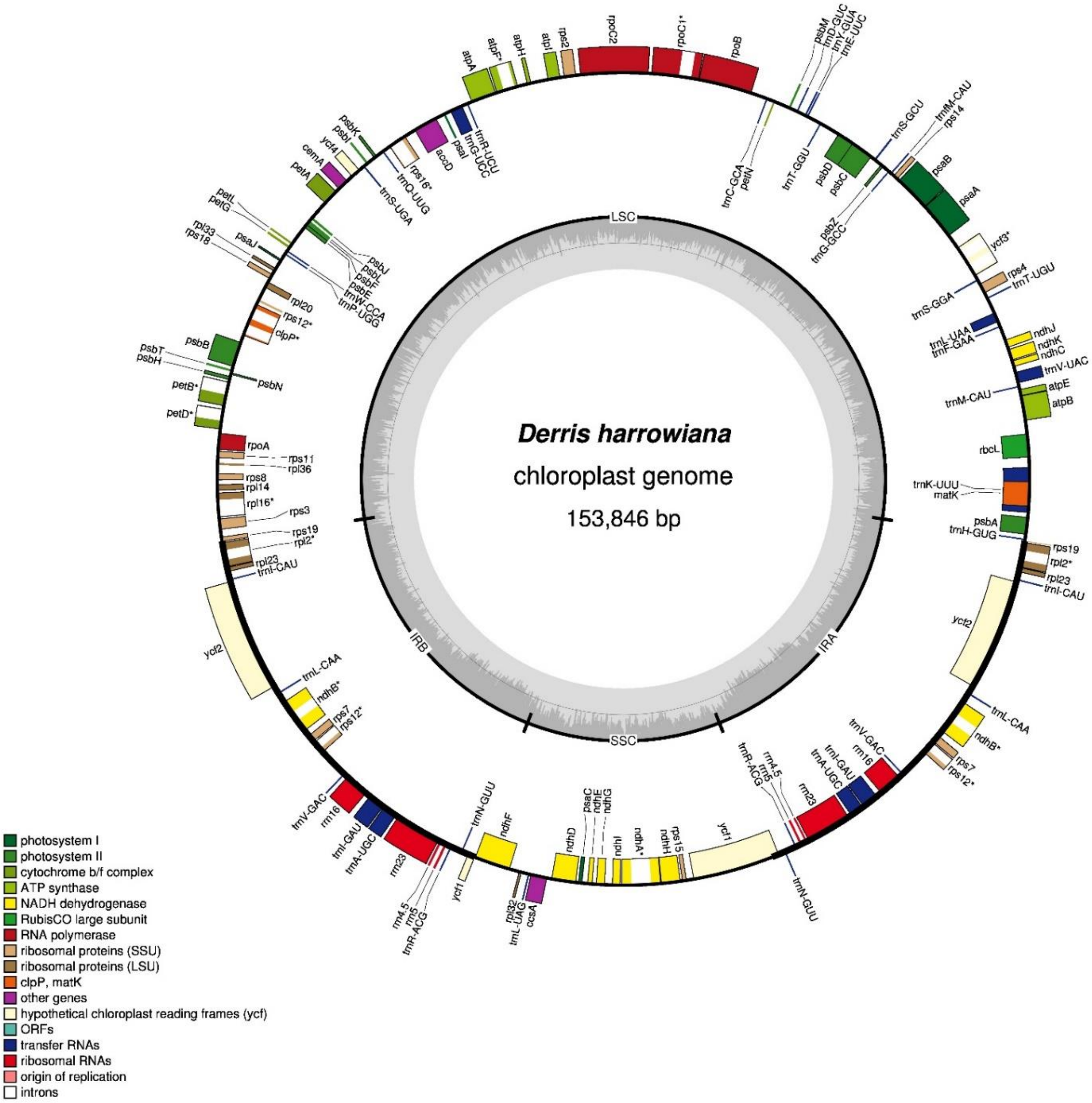

Derris harrowiana
chloroplast genome
153,846 bp
LSC
IRA
SSC
IRB
photosystem I
photosystem II
cytochrome b/f complex
ATP synthase
NADH dehydrogenase
RubisCO large subunit
RNA polymerase
ribosomal proteins (SSU)
ribosomal proteins (LSU)
clpP, matK
other genes
hypothetical chloroplast reading frames (ycf)
ORFs
transfer RNAs
ribosomal RNAs
origin of replication
introns

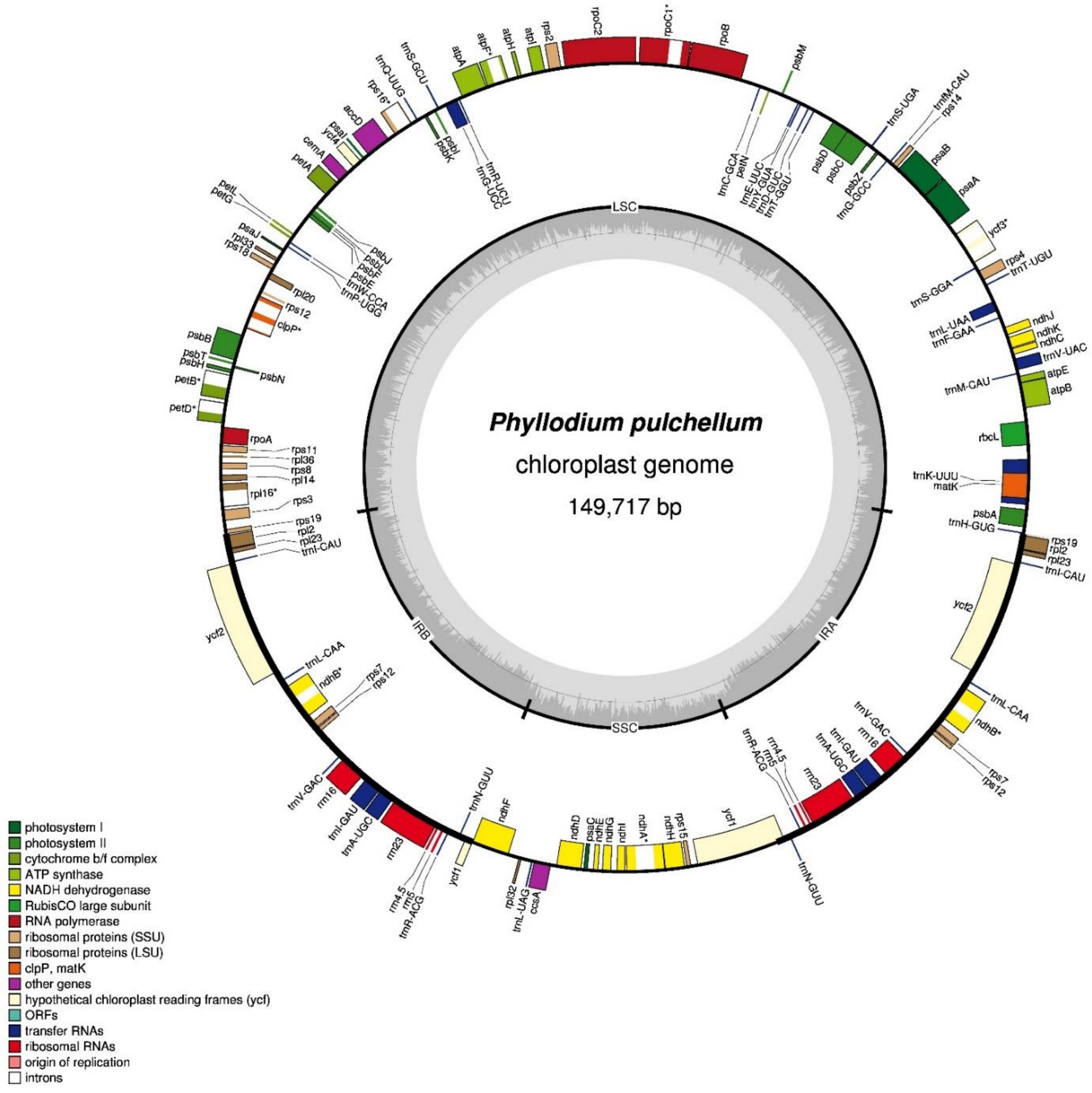

Phyllodium pulchellum
chloroplast genome
149,717 bp
LSC
IRA
SSC
IRB
photosystem I
photosystem II
cytochrome b/f complex
ATP synthase
NADH dehydrogenase
RubisCO large subunit
RNA polymerase
ribosomal proteins (SSU)
ribosomal proteins (LSU)
clpP, matK
other genes
hypothetical chloroplast reading frames (ycf)
ORFs
transfer RNAs
ribosomal RNAs
origin of replication
introns

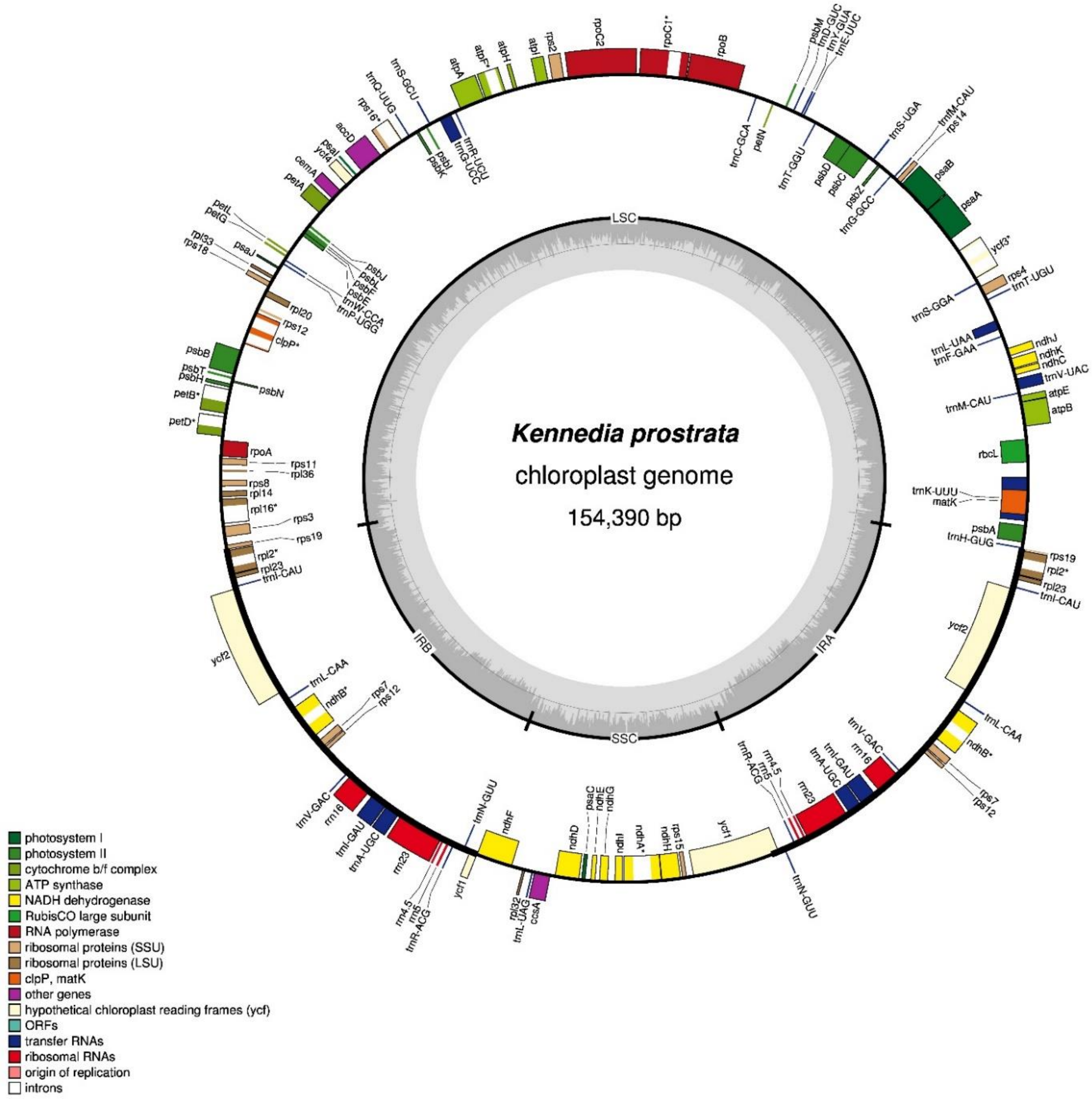

Kennedia prostrata
chloroplast genome
154,390 bp
LSC
IRA
SSC
IRB
photosystem I
photosystem II
cytochrome b/f complex
ATP synthase
NADH dehydrogenase
RubisCO large subunit
RNA polymerase
ribosomal proteins (SSU)
ribosomal proteins (LSU)
clpP, matK
other genes
hypothetical chloroplast reading frames (ycf)
ORFs
transfer RNAs
ribosomal RNAs
origin of replication
introns

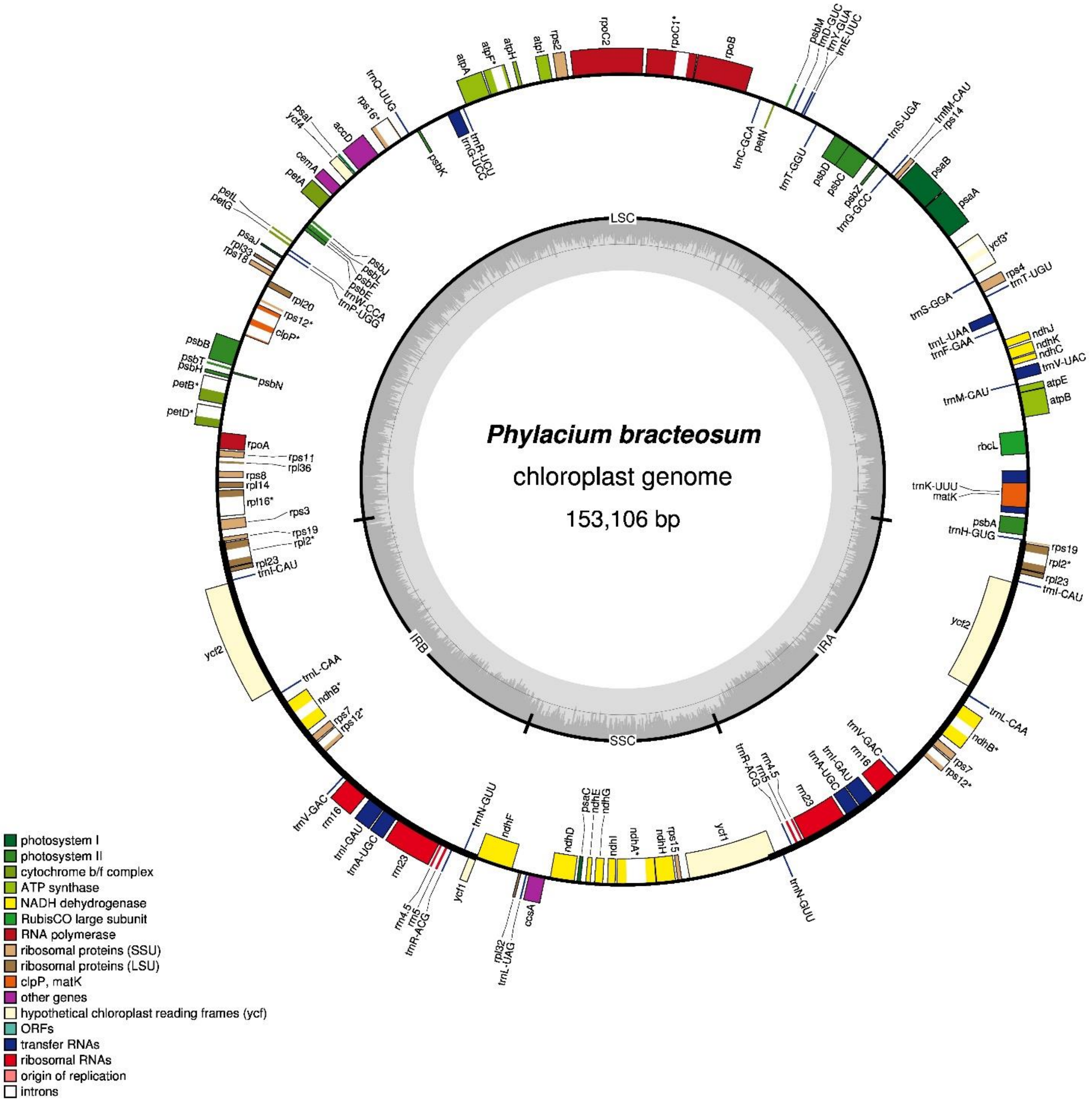
Phylacium bracteosum
chloroplast genome
153,106 bp
LSC
IRA
SSC
IRB
photosystem I
photosystem II
cytochrome b/f complex
ATP synthase
NADH dehydrogenase
RubisCO large subunit
RNA polymerase
ribosomal proteins (SSU)
ribosomal proteins (LSU)
clpP, matK
other genes
hypothetical chloroplast reading frames (ycf)
ORFs
transfer RNAs
ribosomal RNAs
origin of replication
introns
rpoC2
rpoC1*
rpoB
rps2
atpI
atpH
atpF*
atpA
trnQ-UUG
rps16*
accD
psaI
ycf4
cemA
petA
petL
petG
psaJ
rpl33
rps18
rpl20
rps12*
clpP*
psbB
psbT
psbH
psbN
petB*
petD*
rpoA
rps11
rpl36
rps8
rpl14
rpl16*
rps3
rps19
rpl2*
rpl23
trnI-CAU
ycf2
trnL-CAA
ndhB*
rps7
rps12*
trnV-GAC
rrn16
trnI-GAU
trnA-UGC
rrn23
rrn4.5
rrn5
trnR-ACG
ycf1
trnN-GUU
ndhF
rpl32
trnL-UAG
ccsA
ndhD
psaC
ndhE
ndhG
ndhI
ndhA*
ndhH
rps15
psbK
trnR-UCU
trnG-UCC
psbJ
psbL
psbF
psbE
trnW-CCA
trnP-UGG
psbM
trnD-GUC
trnY-GUA
trnE-UUC
trnC-GCA
petN
trnT-GGU
psbD
psbC
psbZ
trnG-GCC
trnS-UGA
trnfM-CAU
rps14
psaB
psaA
ycf3*
rps4
trnT-UGU
trnS-GGA
trnL-UAA
trnF-GAA
ndhJ
ndhK
ndhC
trnV-UAC
atpE
atpB
trnM-CAU
rbcL
trnK-UUU
matK
psbA
trnH-GUG

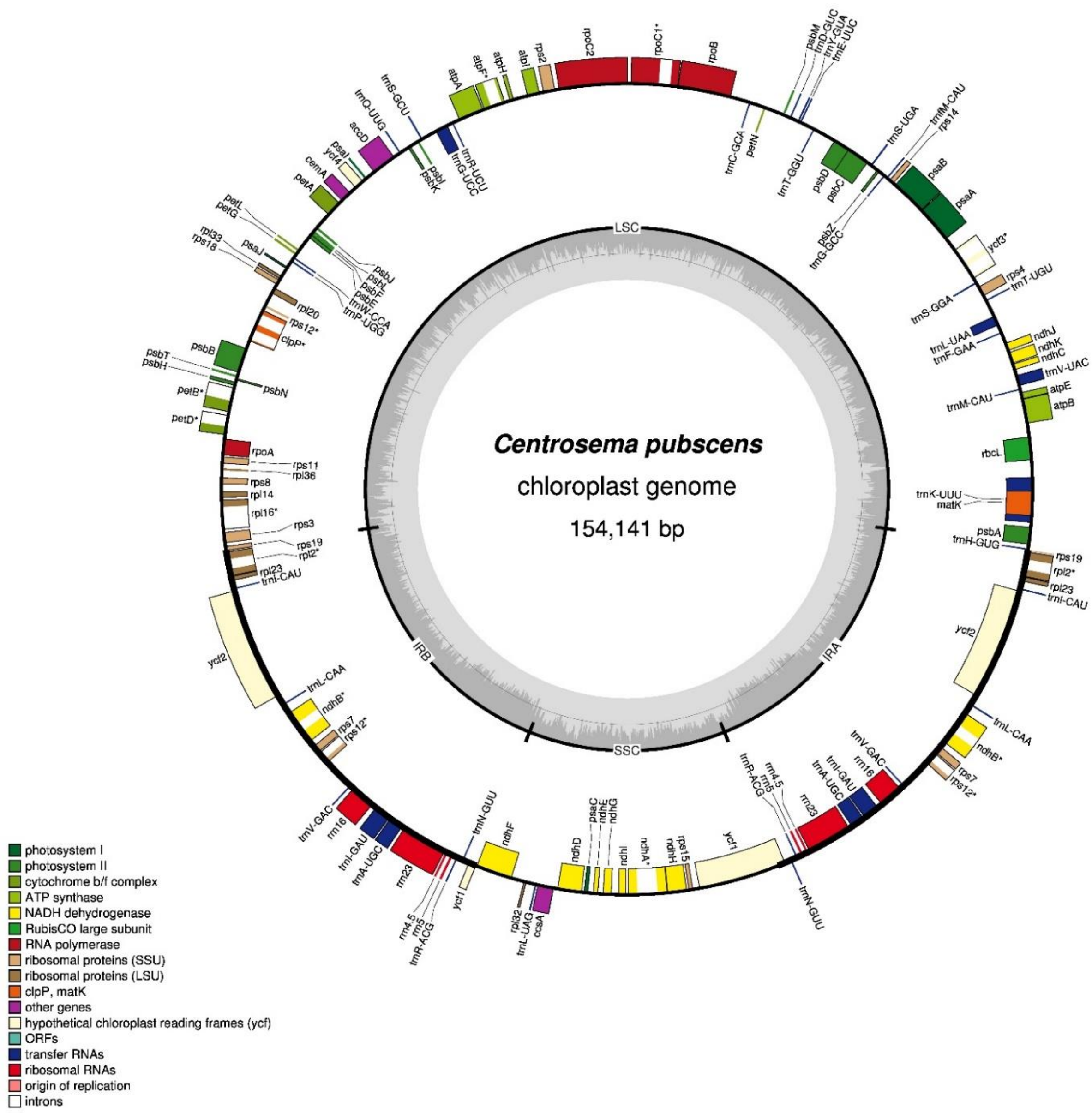

Centrosema pubscens
chloroplast genome
154,141 bp
LSC
IRA
SSC
IRB
rpoC2
rpoC1*
rpoB
rps2
atpI
atpH
atpF*
atpA
trnS-GCU
trnQ-UUG
accD
psaI
ycf4
cemA
petA
petL
petG
rpl33
rps18
psaJ
psbJ
psbL
psbF
psbE
trnW-CCA
trnP-UGG
rpl20
rps12*
clpP*
psbB
psbT
psbH
psbN
petB*
petD*
rpoA
rps11
rpl36
rps8
rpl14
rpl16*
rps3
rps19
rpl2*
rpl23
trnI-CAU
ycf2
trnL-CAA
ndhB*
rps7
rps12*
trnV-GAC
rrn16
trnI-GAU
trnA-UGC
rrn23
rrn4.5
rrn5
trnR-ACG
trnN-GUU
ycf1
ndhF
rpl32
trnL-UAG
ccsA
ndhD
psaC
ndhE
ndhG
ndhI
ndhA*
ndhH
rps15
psbI
psbK
trnR-UCU
trnG-UCC
psbM
trnD-GUC
trnY-GUA
trnE-UUC
trnC-GCA
petN
trnT-GGU
psbD
psbC
psbZ
trnG-GCC
trnS-UGA
trnfM-CAU
rps14
psaB
psaA
ycf3*
rps4
trnT-UGU
trnS-GGA
trnL-UAA
trnF-GAA
ndhJ
ndhK
ndhC
trnV-UAC
trnM-CAU
atpE
atpB
rbcL
trnK-UUU
matK
psbA
trnH-GUG
photosystem I
photosystem II
cytochrome b/f complex
ATP synthase
NADH dehydrogenase
RubisCO large subunit
RNA polymerase
ribosomal proteins (SSU)
ribosomal proteins (LSU)
clpP, matK
other genes
hypothetical chloroplast reading frames (ycf)
ORFs
transfer RNAs
ribosomal RNAs
origin of replication
introns

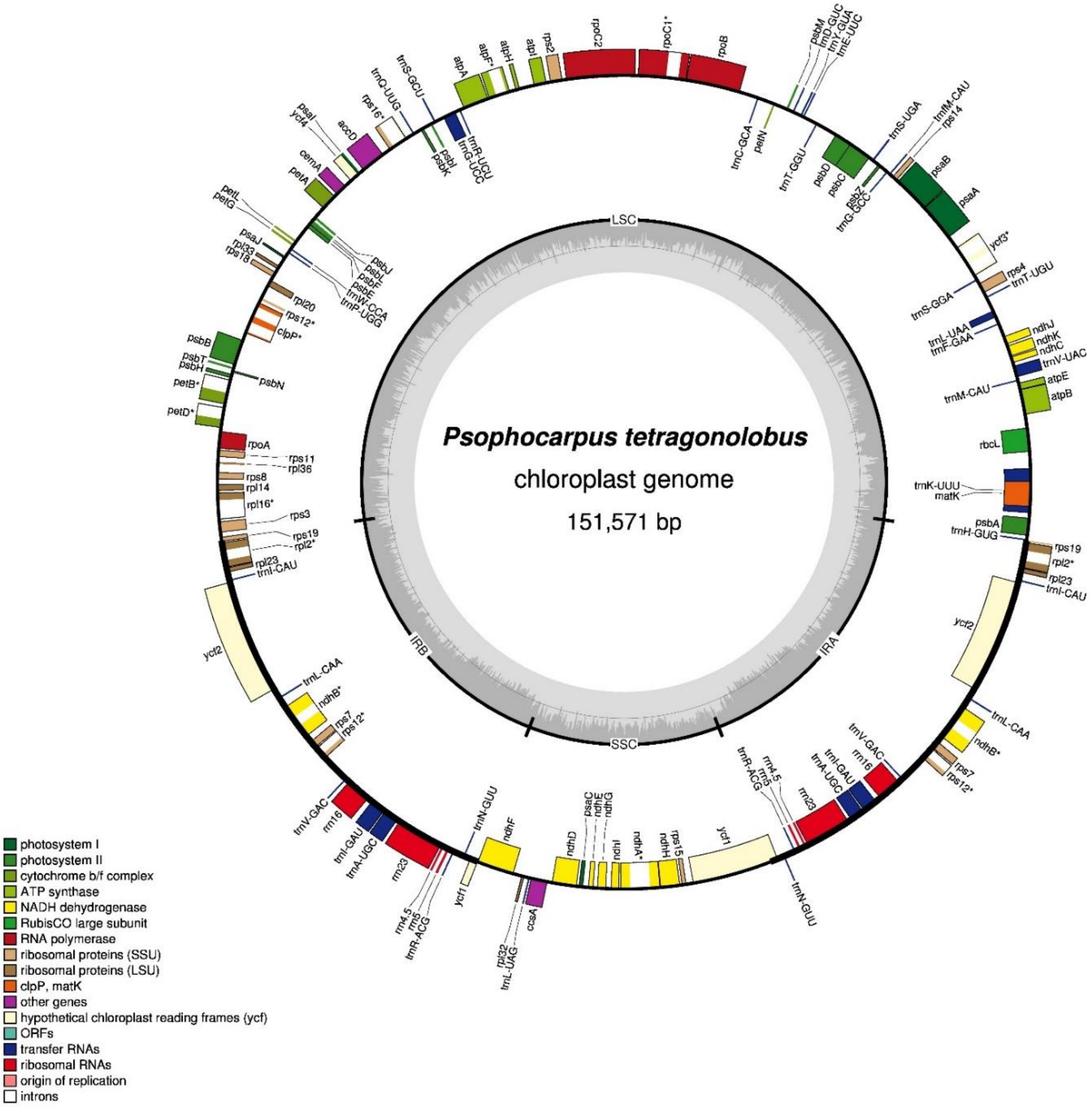
Psophocarpus tetragonolobus
chloroplast genome
151,571 bp
LSC
SSC
IRA
IRB
rpoC2
rpoC1*
rpoB
psbM
trnD-GUC
trnY-GUA
trnE-UUC
trnC-GCA
petN
trnT-GGU
psbD
psbC
psbZ
trnG-GCC
trnS-UGA
trnfM-CAU
rps14
psaB
psaA
ycf3*
rps4
trnT-UGU
trnS-GGA
trnL-UAA
trnF-GAA
ndhJ
ndhK
ndhC
trnV-UAC
trnM-CAU
atpE
atpB
rbcL
trnK-UUU
matK
psbA
trnH-GUG
rps19
rpl2*
rpl23
trnI-CAU
ycf2
trnL-CAA
ndhB*
rps7
rps12*
trnV-GAC
rrn16
trnI-GAU
trnA-UGC
rrn23
rrn4.5
rrn5
trnR-ACG
trnN-GUU
ycf1
rps15
ndhH
ndhA*
ndhI
ndhG
ndhE
psaC
ndhD
ccsA
rpl32
trnL-UAG
ndhF
rpl16*
rpl14
rps8
rpl36
rps11
rpoA
rps3
petD*
petB*
psbH
psbT
psbN
psbB
clpP*
rps12*
rpl20
rps18
rpl33
psaJ
trnP-UGG
trnW-CCA
psbE
psbF
psbL
psbJ
petG
petL
petA
cemA
ycf4
psaI
accD
rps16*
trnQ-UUG
trnS-GCU
psbK
psbI
trnG-UCC
trnR-UCU
atpA
atpF*
atpH
atpI
rps2
photosystem I
photosystem II
cytochrome b/f complex
ATP synthase
NADH dehydrogenase
RubisCO large subunit
RNA polymerase
ribosomal proteins (SSU)
ribosomal proteins (LSU)
clpP, matK
other genes
hypothetical chloroplast reading frames (ycf)
ORFs
transfer RNAs
ribosomal RNAs
origin of replication
introns

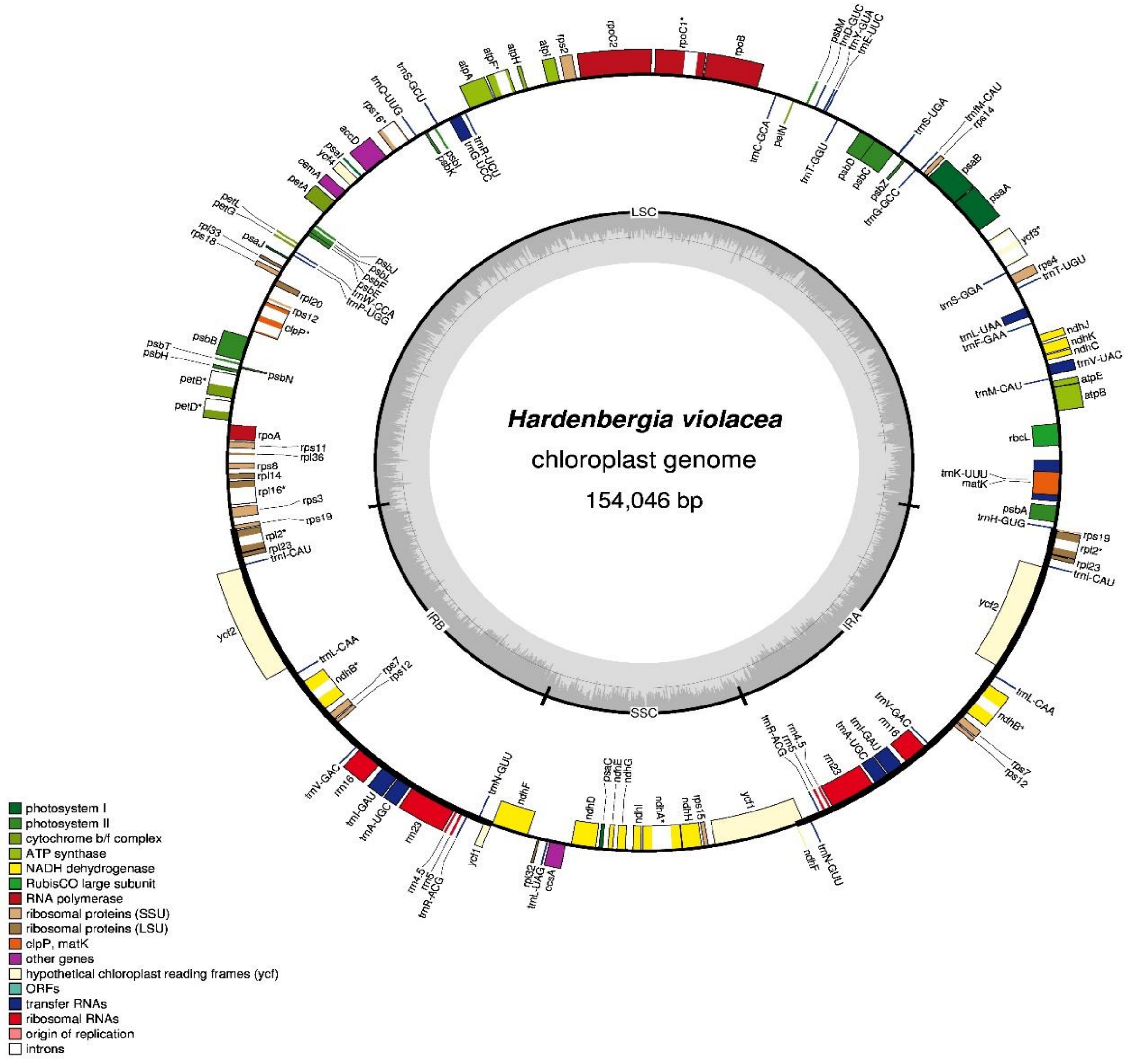

Hardenbergia violacea
chloroplast genome
154,046 bp
LSC
SSC
IRA
IRB
photosystem I
photosystem II
cytochrome b/f complex
ATP synthase
NADH dehydrogenase
RubisCO large subunit
RNA polymerase
ribosomal proteins (SSU)
ribosomal proteins (LSU)
clpP, matK
other genes
hypothetical chloroplast reading frames (ycf)
ORFs
transfer RNAs
ribosomal RNAs
origin of replication
introns

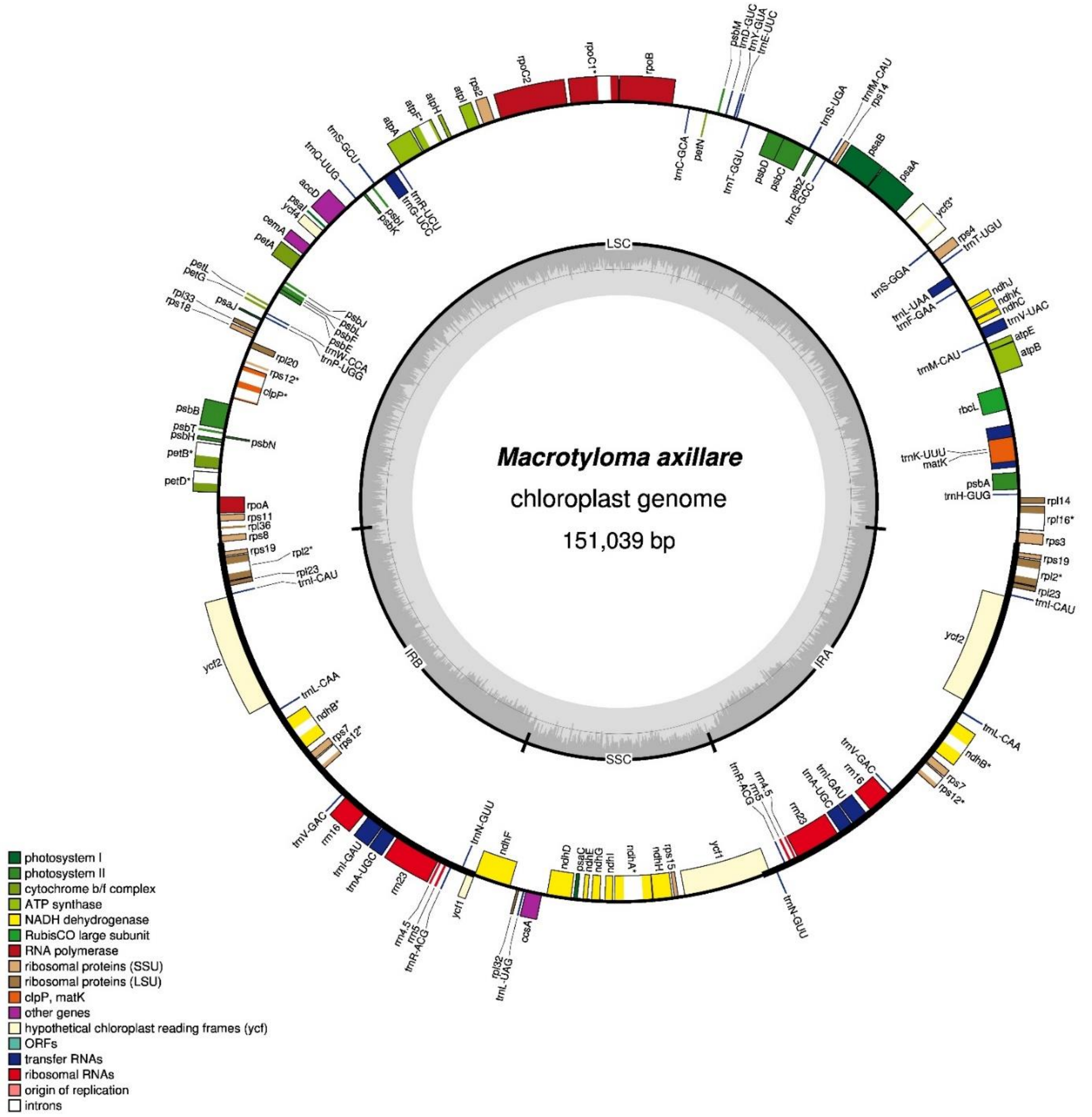
Macrotyloma axillare
chloroplast genome
151,039 bp
LSC
IRA
IRB
SSC
photosystem I
photosystem II
cytochrome b/f complex
ATP synthase
NADH dehydrogenase
RubisCO large subunit
RNA polymerase
ribosomal proteins (SSU)
ribosomal proteins (LSU)
clpP, matK
other genes
hypothetical chloroplast reading frames (ycf)
ORFs
transfer RNAs
ribosomal RNAs
origin of replication
introns

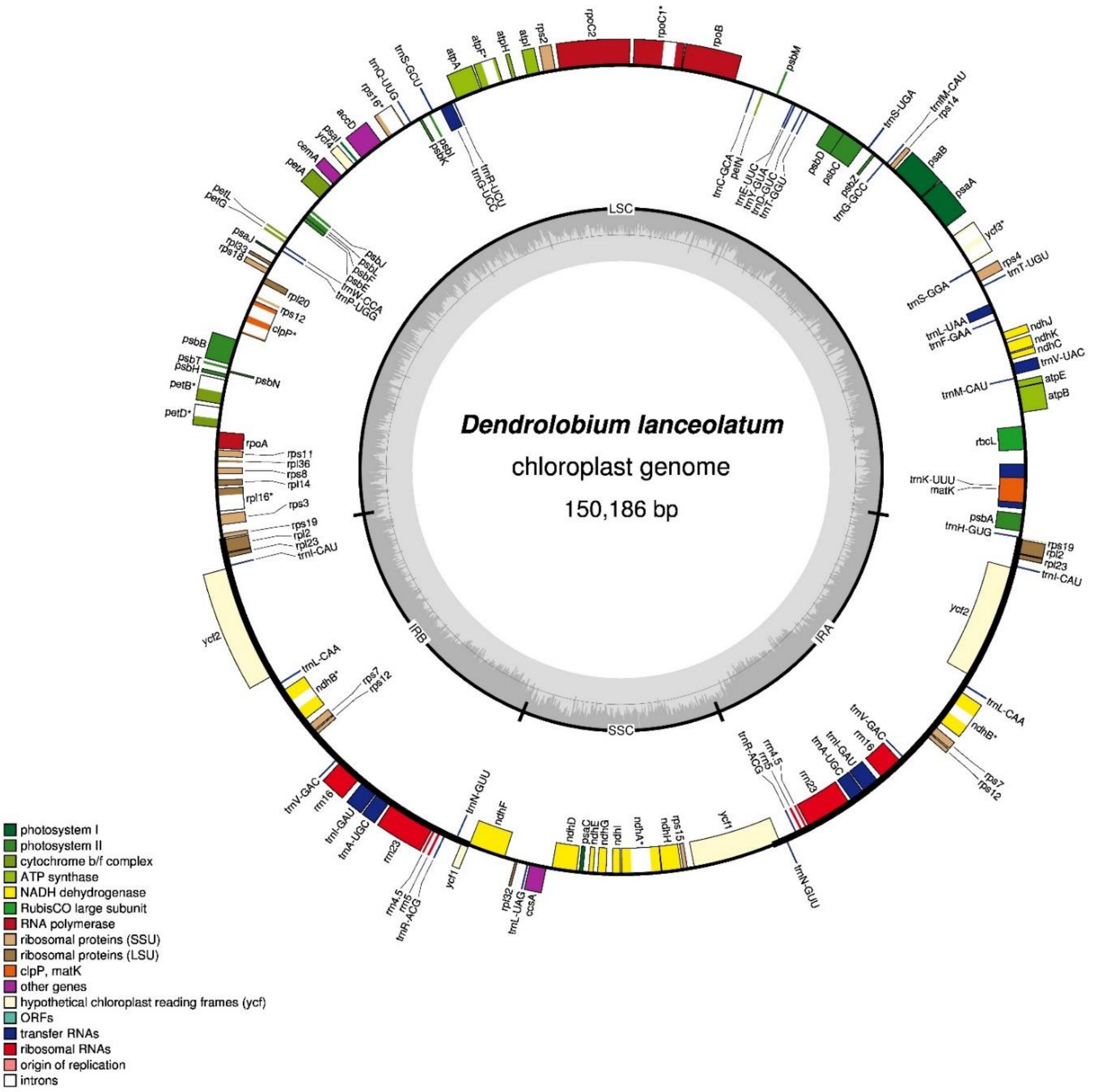

Dendrolobium lanceolatum
chloroplast genome
150,186 bp
LSC
IRA
SSC
IRB
photosystem I
photosystem II
cytochrome b/f complex
ATP synthase
NADH dehydrogenase
RubisCO large subunit
RNA polymerase
ribosomal proteins (SSU)
ribosomal proteins (LSU)
clpP, matK
other genes
hypothetical chloroplast reading frames (ycf)
ORFs
transfer RNAs
ribosomal RNAs
origin of replication
introns

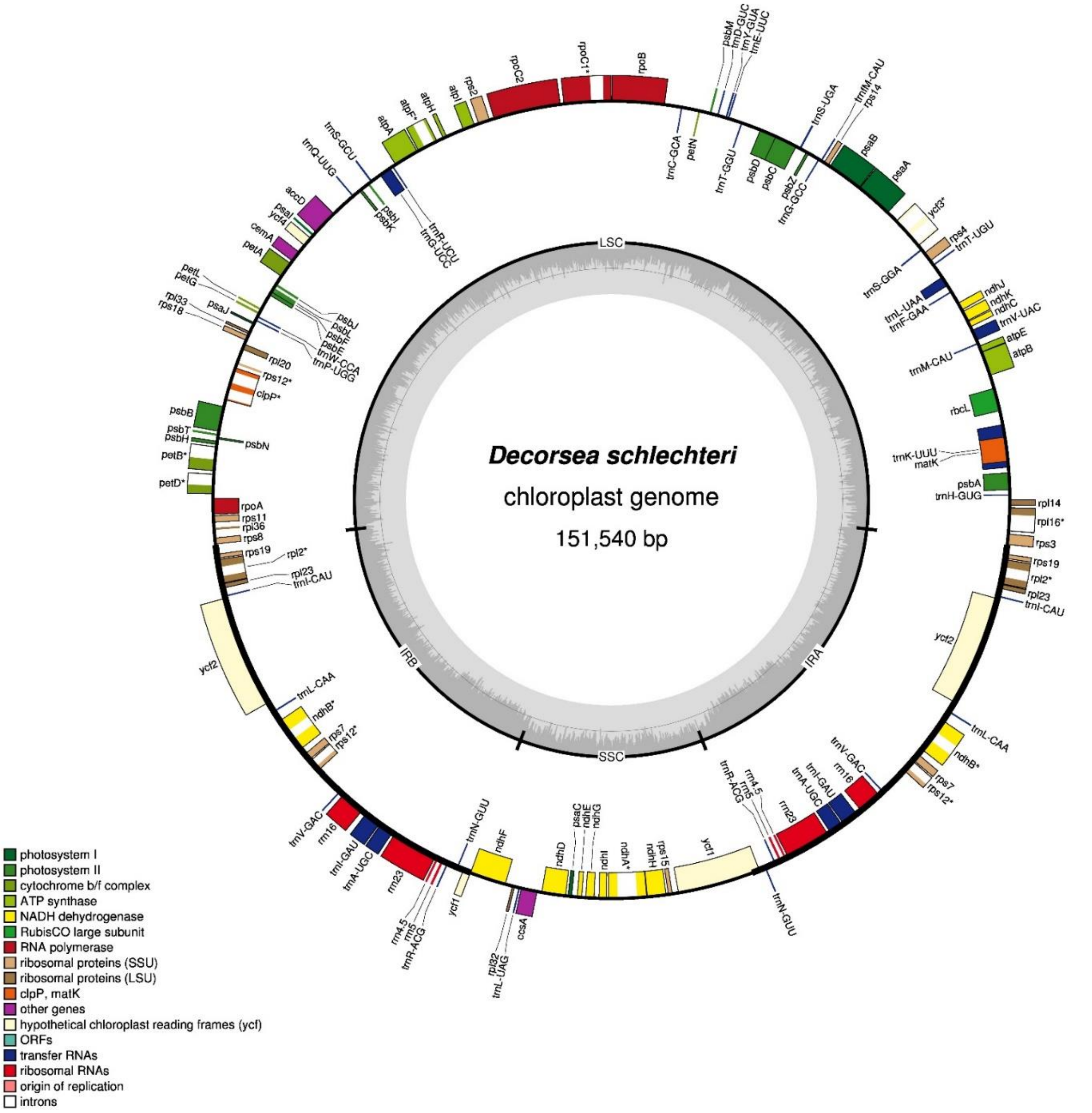

Decorsea schlechteri
chloroplast genome
151,540 bp
LSC
SSC
IRA
IRB
photosystem I
photosystem II
cytochrome b/f complex
ATP synthase
NADH dehydrogenase
RubisCO large subunit
RNA polymerase
ribosomal proteins (SSU)
ribosomal proteins (LSU)
clpP, matK
other genes
hypothetical chloroplast reading frames (ycf)
ORFs
transfer RNAs
ribosomal RNAs
origin of replication
introns

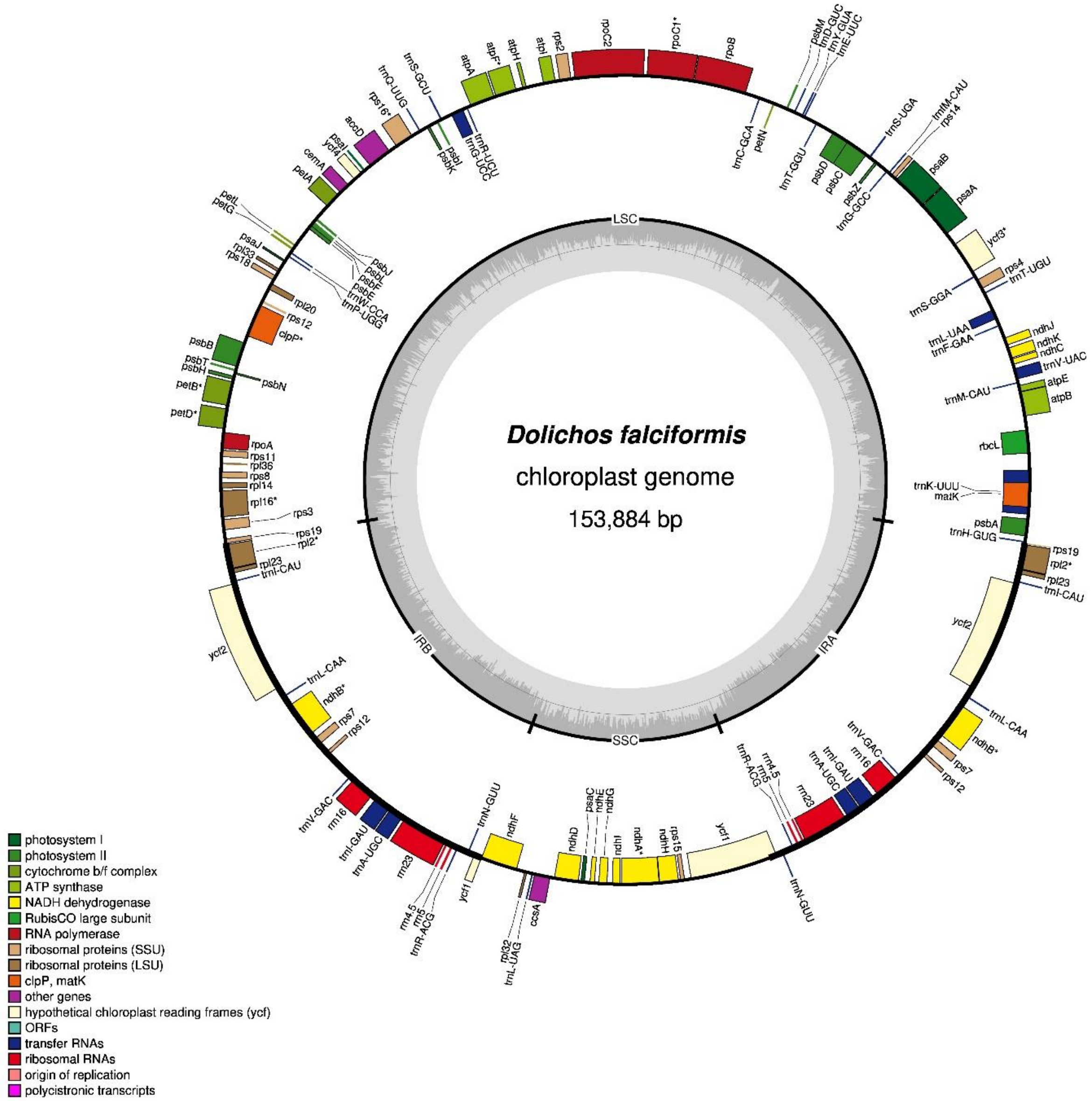
Dolichos falciformis
chloroplast genome
153,884 bp
LSC
SSC
IRA
IRB
rpoC2
rpoC1*
rpoB
rps2
atpI
atpH
atpF*
atpA
trnS-GCU
trnQ-UUG
rps16*
accD
psaI
ycf4
cemA
petA
petL
petG
psaJ
rpl33
rps18
rpl20
rps12
clpP*
psbB
psbT
psbH
petB*
petD*
psbN
rpoA
rps11
rpl36
rps8
rpl14
rpl16*
rps3
rps19
rpl2*
rpl23
trnI-CAU
ycf2
trnL-CAA
ndhB*
rps7
trnV-GAC
rrn16
trnI-GAU
trnA-UGC
rrn23
rrn4.5
rrn5
trnR-ACG
trnN-GUU
ycf1
ndhF
rpl32
trnL-UAG
ccsA
ndhD
psaC
ndhE
ndhG
ndhI
ndhA*
ndhH
rps15
psbM
trnD-GUC
trnY-GUA
trnE-UUC
trnC-GCA
petN
trnT-GGU
psbD
psbC
psbZ
trnG-GCC
trnS-UGA
trnfM-CAU
rps14
psaB
psaA
ycf3*
rps4
trnT-UGU
trnS-GGA
trnL-UAA
trnF-GAA
ndhJ
ndhK
ndhC
trnV-UAC
trnM-CAU
atpE
atpB
rbcL
trnK-UUU
matK
psbA
trnH-GUG
photosystem I
photosystem II
cytochrome b/f complex
ATP synthase
NADH dehydrogenase
RubisCO large subunit
RNA polymerase
ribosomal proteins (SSU)
ribosomal proteins (LSU)
clpP, matK
other genes
hypothetical chloroplast reading frames (ycf)
ORFs
transfer RNAs
ribosomal RNAs
origin of replication
polycistronic transcripts

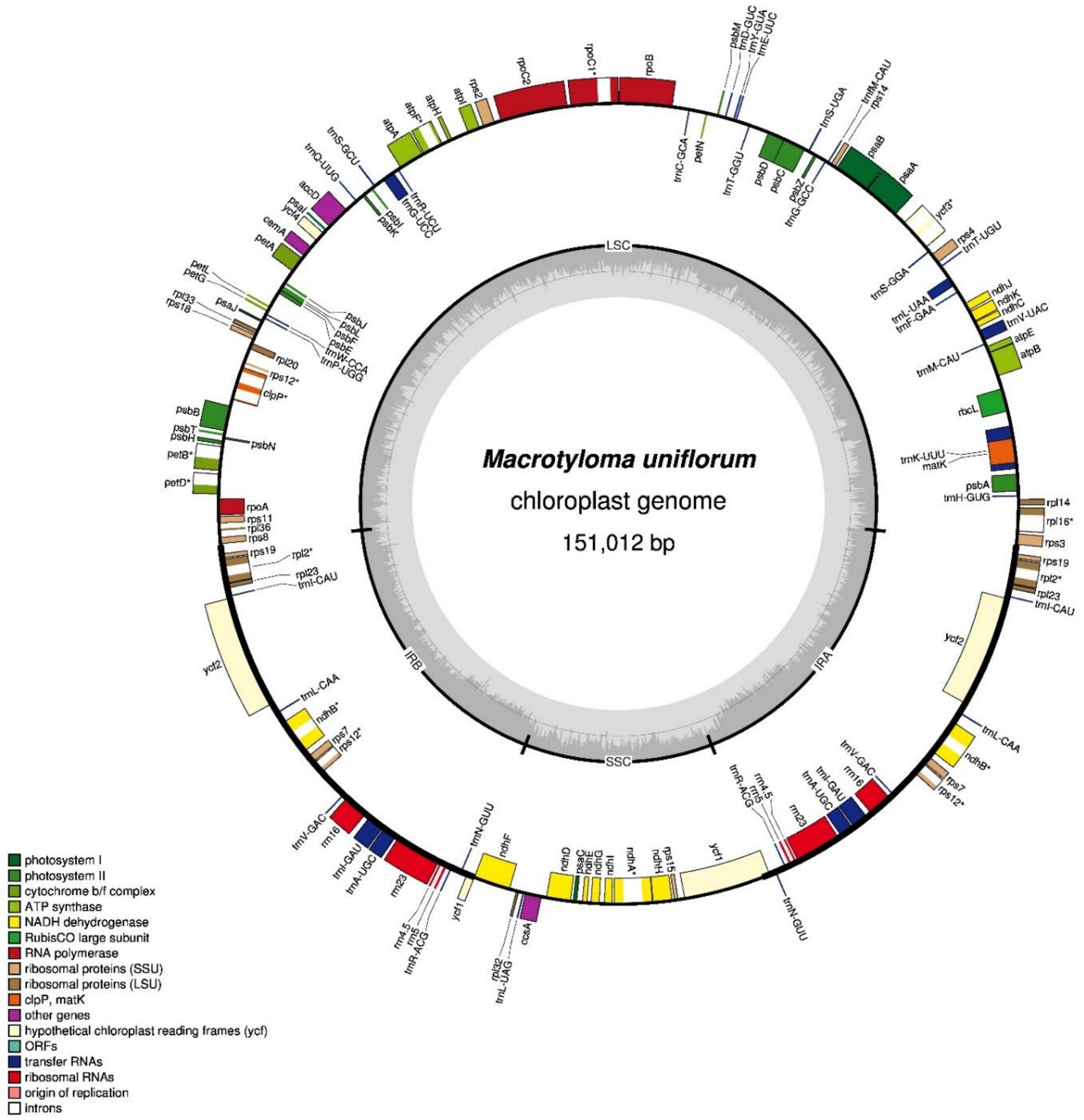
Macrotyloma uniflorum
chloroplast genome
151,012 bp
LSC
IRA
SSC
IRB
photosystem I
photosystem II
cytochrome b/f complex
ATP synthase
NADH dehydrogenase
RubisCO large subunit
RNA polymerase
ribosomal proteins (SSU)
ribosomal proteins (LSU)
clpP, matK
other genes
hypothetical chloroplast reading frames (ycf)
ORFs
transfer RNAs
ribosomal RNAs
origin of replication
introns

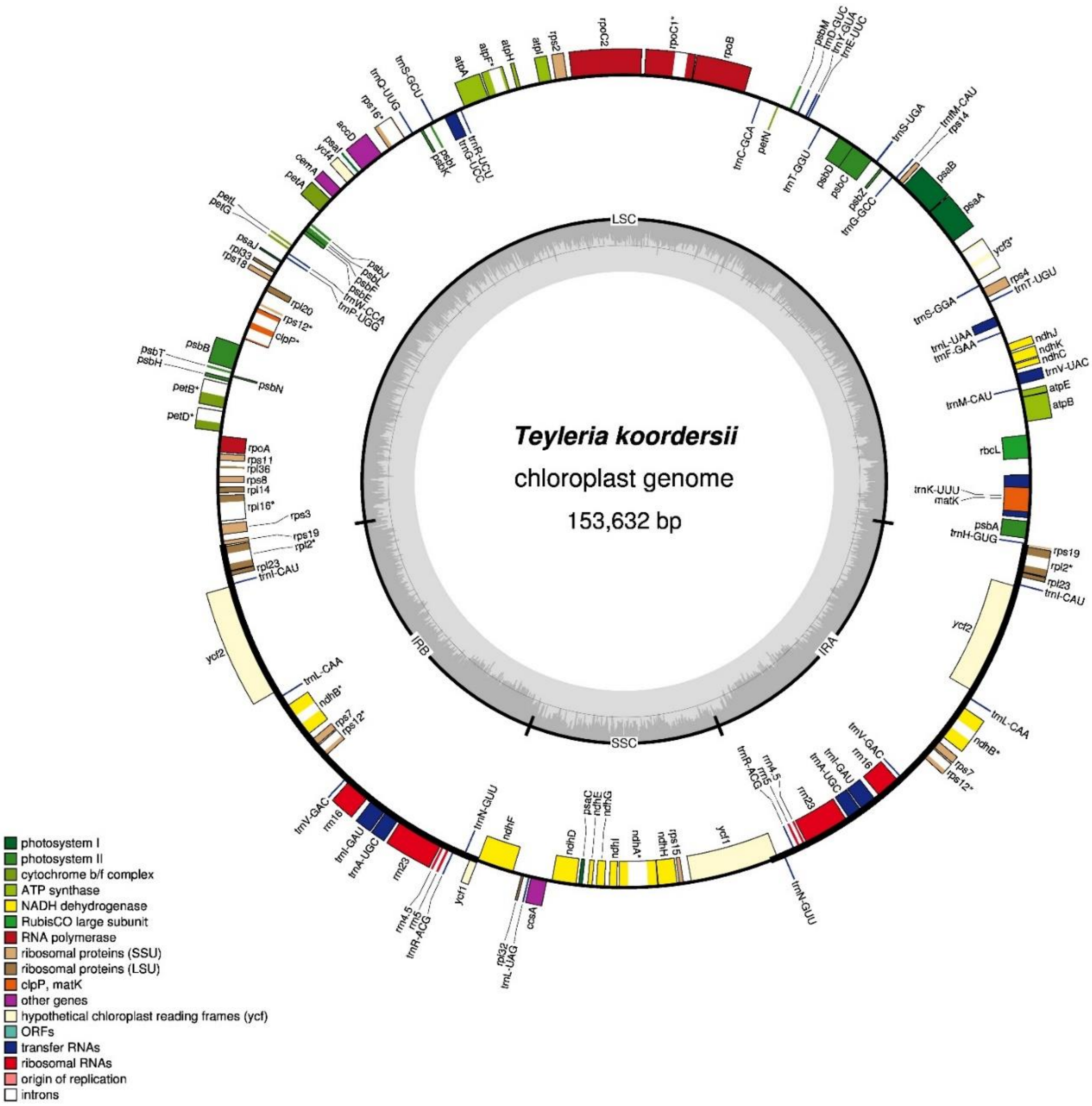
Teyleria koordersii
chloroplast genome
153,632 bp
LSC
SSC
IRA
IRB
photosystem I
photosystem II
cytochrome b/f complex
ATP synthase
NADH dehydrogenase
RubisCO large subunit
RNA polymerase
ribosomal proteins (SSU)
ribosomal proteins (LSU)
clpP, matK
other genes
hypothetical chloroplast reading frames (ycf)
ORFs
transfer RNAs
ribosomal RNAs
origin of replication
introns

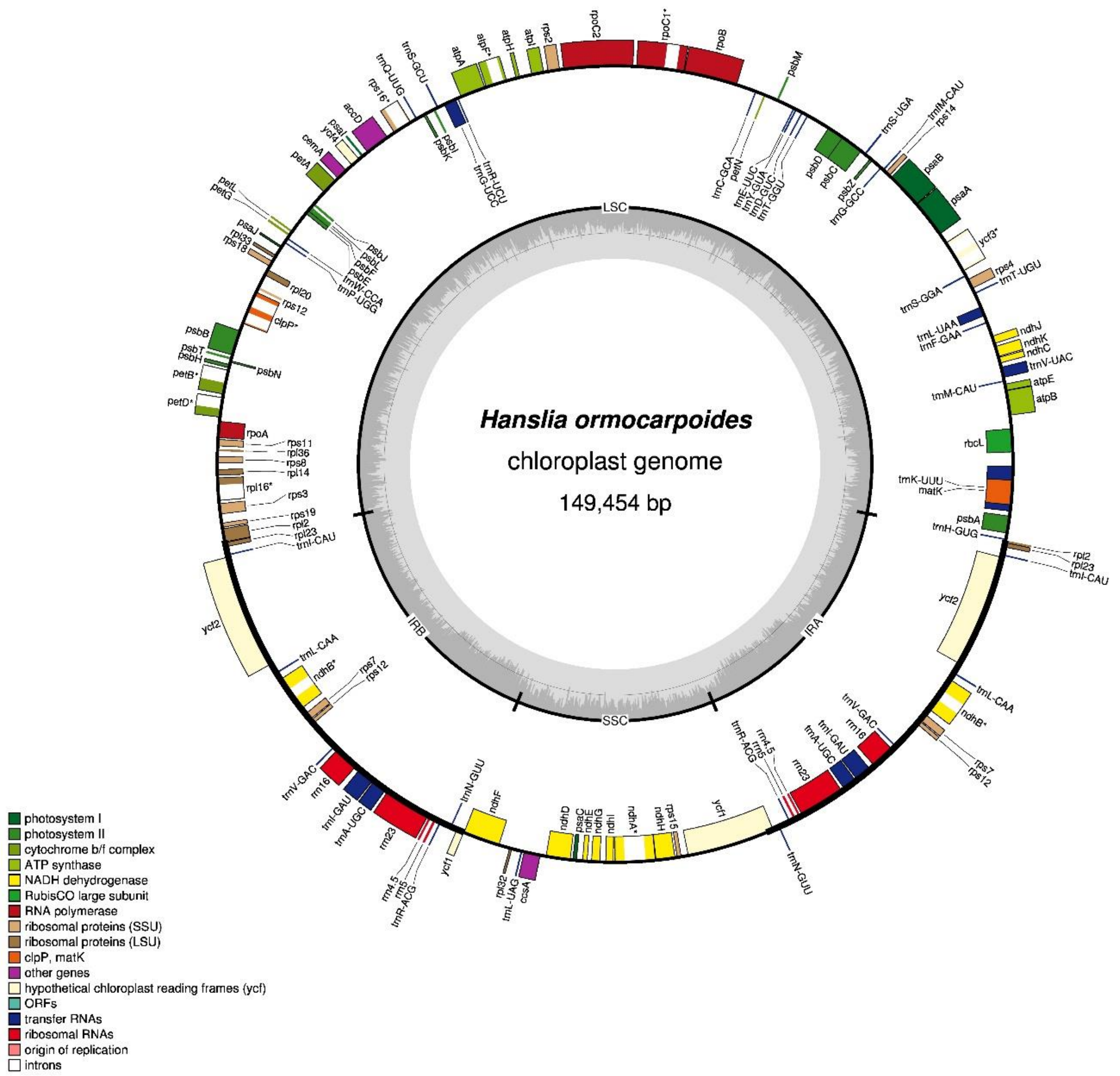
Hanslia ormocarpoides
chloroplast genome
149,454 bp
LSC
IRA
SSC
IRB
rpoC2
rpoC1*
rpoB
rps2
atpI
atpH
atpF*
atpA
trnS-GCU
trnQ-UUG
rps16*
accD
psaI
ycf4
cemA
petA
petL
petG
psaJ
rpl33
rps18
rpl20
rps12
clpP*
psbB
psbT
psbH
psbN
petB*
petD*
rpoA
rps11
rpl36
rps8
rpl14
rpl16*
rps3
rps19
rpl2
rpl23
trnI-CAU
ycf2
trnL-CAA
ndhB*
rps7
rps12
trnV-GAC
rrn16
trnI-GAU
trnA-UGC
rrn23
rrn4.5
rrn5
trnR-ACG
ycf1
trnN-GUU
ndhF
rpl32
trnL-UAG
ccsA
ndhD
psaC
ndhE
ndhG
ndhI
ndhA*
ndhH
rps15
psbM
trnC-GCA
petN
trnE-UUC
trnY-GUA
trnD-GUC
trnT-GGU
psbD
psbC
psbZ
trnG-GCC
trnS-UGA
trnfM-CAU
rps14
psaB
psaA
ycf3*
rps4
trnT-UGU
trnS-GGA
trnL-UAA
trnF-GAA
ndhJ
ndhK
ndhC
trnV-UAC
trnM-CAU
atpE
atpB
rbcL
trnK-UUU
matK
psbA
trnH-GUG
psbI
psbK
trnR-UCU
trnG-UCC
psbJ
psbL
psbF
psbE
trnW-CCA
trnP-UGG
photosystem I
photosystem II
cytochrome b/f complex
ATP synthase
NADH dehydrogenase
RubisCO large subunit
RNA polymerase
ribosomal proteins (SSU)
ribosomal proteins (LSU)
clpP, matK
other genes
hypothetical chloroplast reading frames (ycf)
ORFs
transfer RNAs
ribosomal RNAs
origin of replication
introns

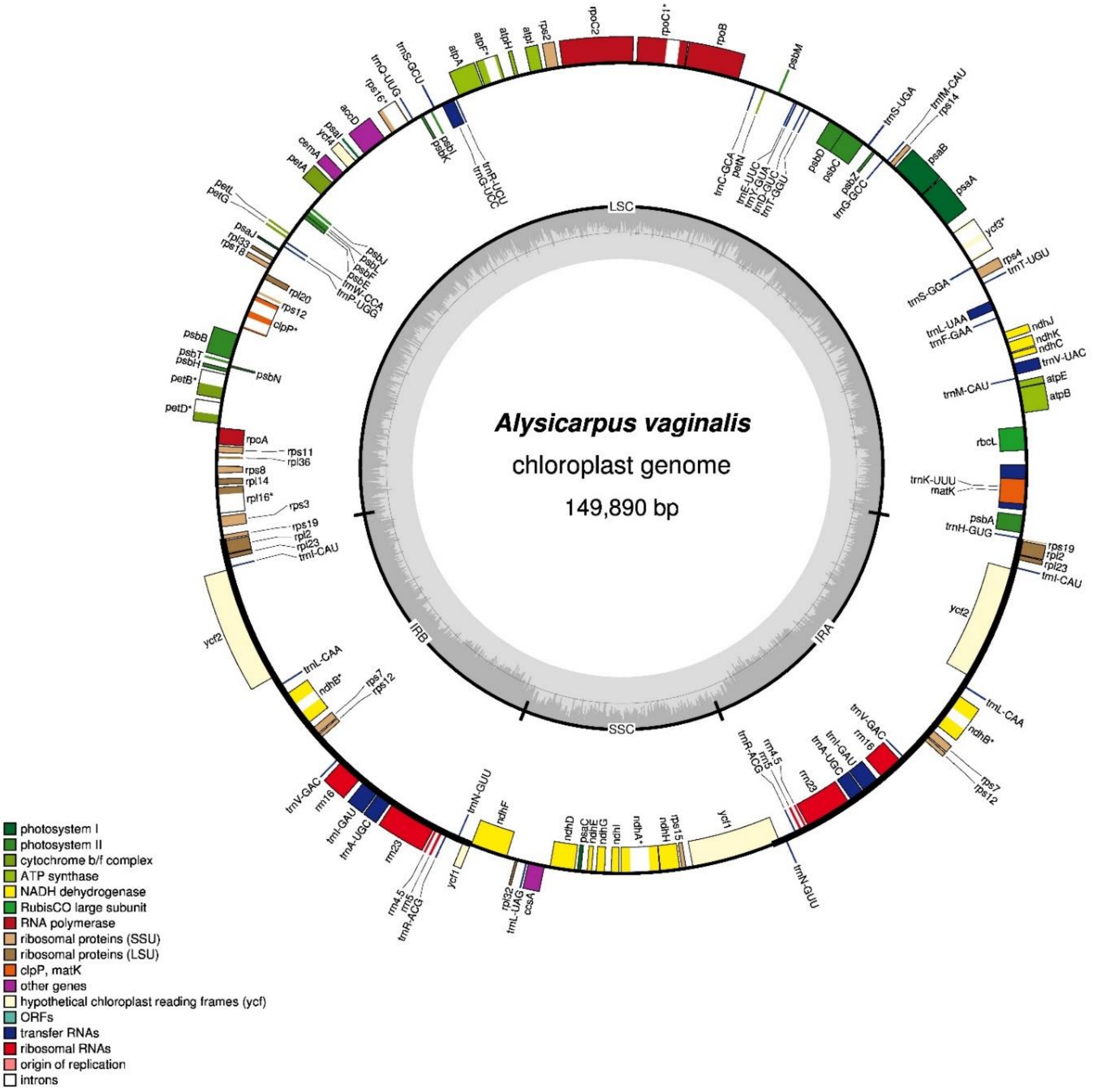

Alysicarpus vaginalis
chloroplast genome
149,890 bp
LSC
IRA
SSC
IRB
photosystem I
photosystem II
cytochrome b/f complex
ATP synthase
NADH dehydrogenase
RubisCO large subunit
RNA polymerase
ribosomal proteins (SSU)
ribosomal proteins (LSU)
clpP, matK
other genes
hypothetical chloroplast reading frames (ycf)
ORFs
transfer RNAs
ribosomal RNAs
origin of replication
introns

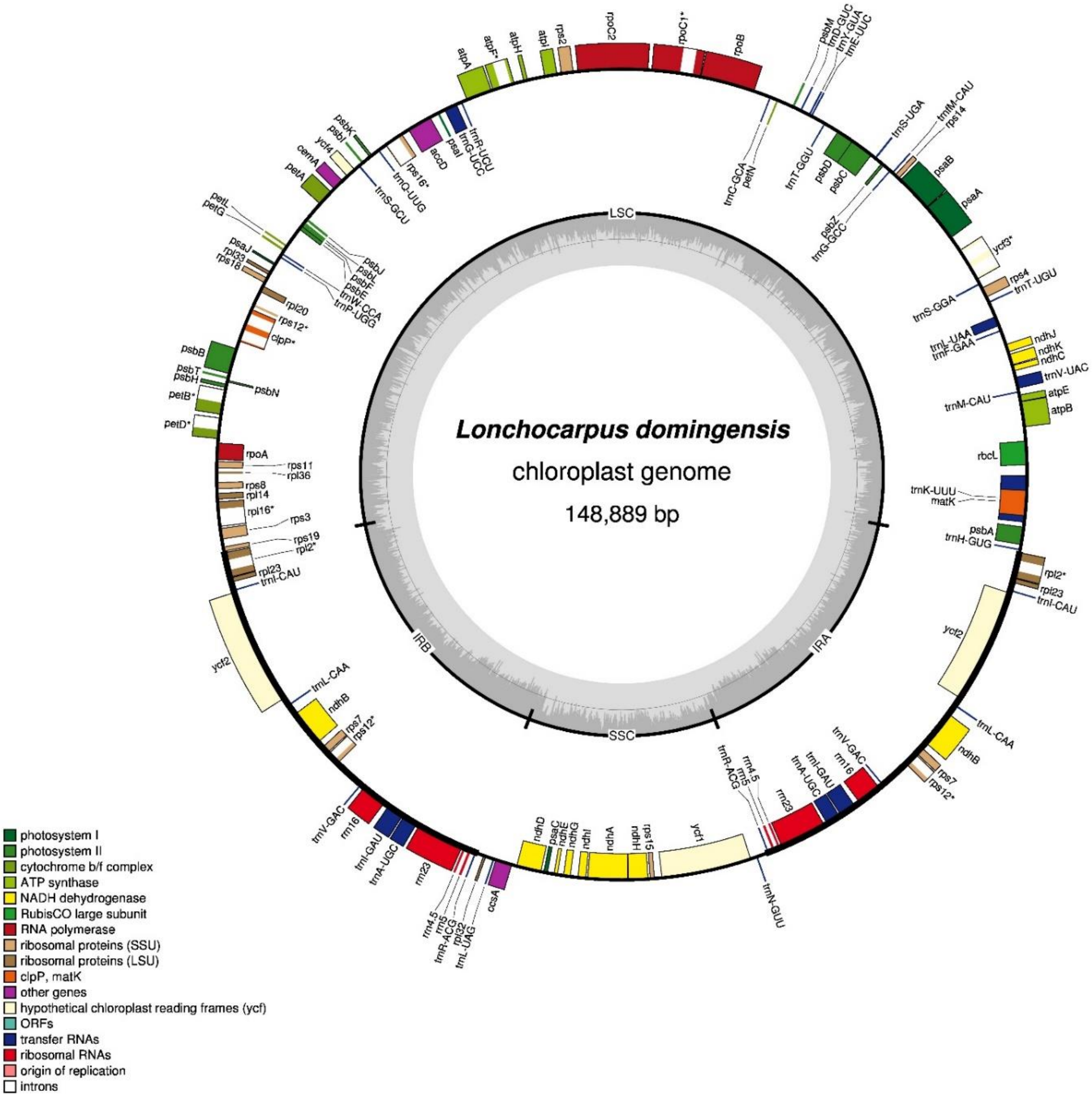
Lonchocarpus domingensis
chloroplast genome
148,889 bp
LSC
IRA
SSC
IRB
photosystem I
photosystem II
cytochrome b/f complex
ATP synthase
NADH dehydrogenase
RubisCO large subunit
RNA polymerase
ribosomal proteins (SSU)
ribosomal proteins (LSU)
clpP, matK
other genes
hypothetical chloroplast reading frames (ycf)
ORFs
transfer RNAs
ribosomal RNAs
origin of replication
introns

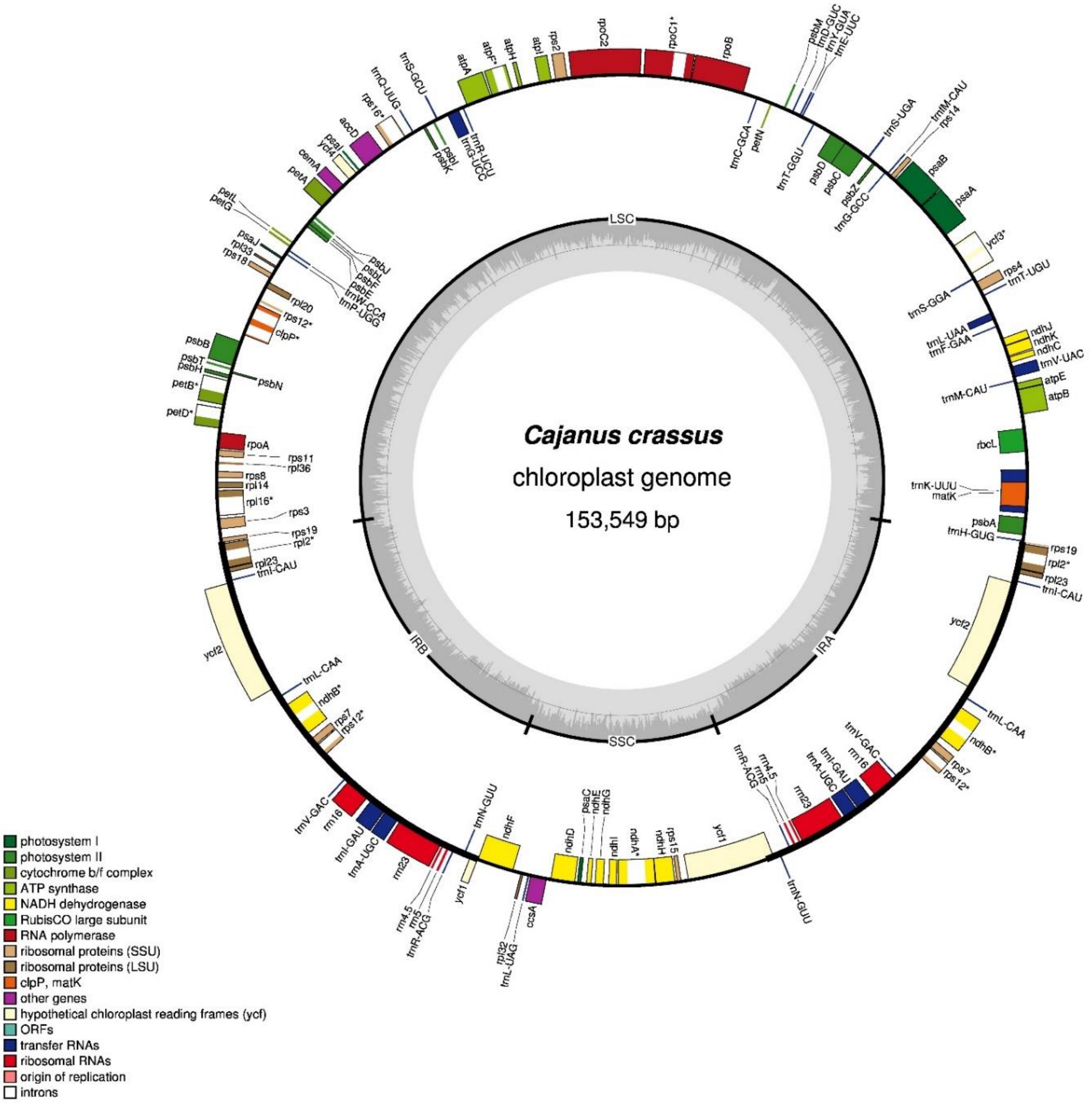

Cajanus crassus
chloroplast genome
153,549 bp
LSC
IRA
SSC
IRB
photosystem I
photosystem II
cytochrome b/f complex
ATP synthase
NADH dehydrogenase
RubisCO large subunit
RNA polymerase
ribosomal proteins (SSU)
ribosomal proteins (LSU)
clpP, matK
other genes
hypothetical chloroplast reading frames (ycf)
ORFs
transfer RNAs
ribosomal RNAs
origin of replication
introns

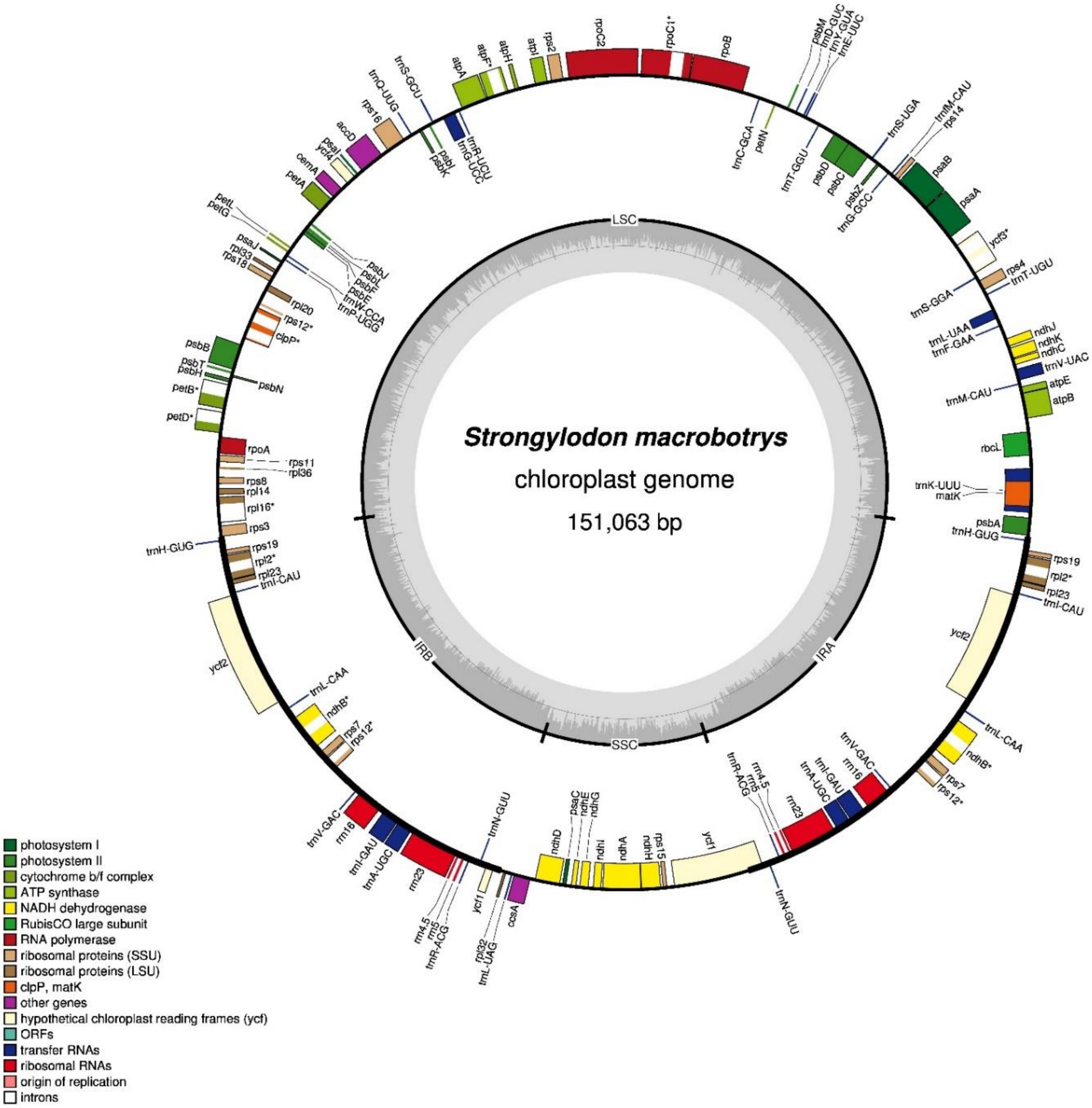

Strongylodon macrobotrys
chloroplast genome
151,063 bp
LSC
IRA
IRB
SSC
photosystem I
photosystem II
cytochrome b/f complex
ATP synthase
NADH dehydrogenase
RubisCO large subunit
RNA polymerase
ribosomal proteins (SSU)
ribosomal proteins (LSU)
clpP, matK
other genes
hypothetical chloroplast reading frames (ycf)
ORFs
transfer RNAs
ribosomal RNAs
origin of replication
introns

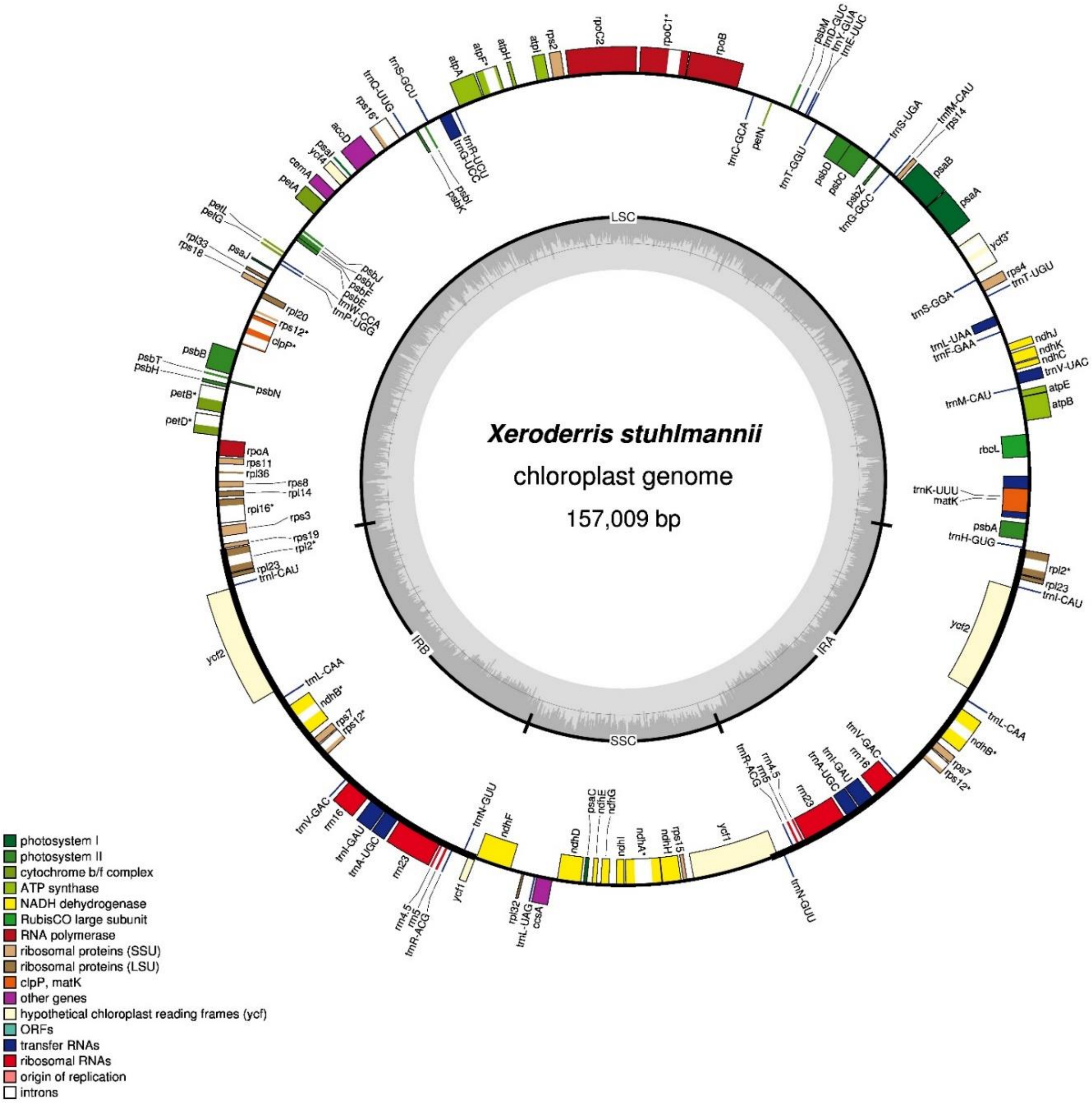

Xeroderris stuhlmannii
chloroplast genome
157,009 bp
LSC
SSC
IRA
IRB
photosystem I
photosystem II
cytochrome b/f complex
ATP synthase
NADH dehydrogenase
RubisCO large subunit
RNA polymerase
ribosomal proteins (SSU)
ribosomal proteins (LSU)
clpP, matK
other genes
hypothetical chloroplast reading frames (ycf)
ORFs
transfer RNAs
ribosomal RNAs
origin of replication
introns

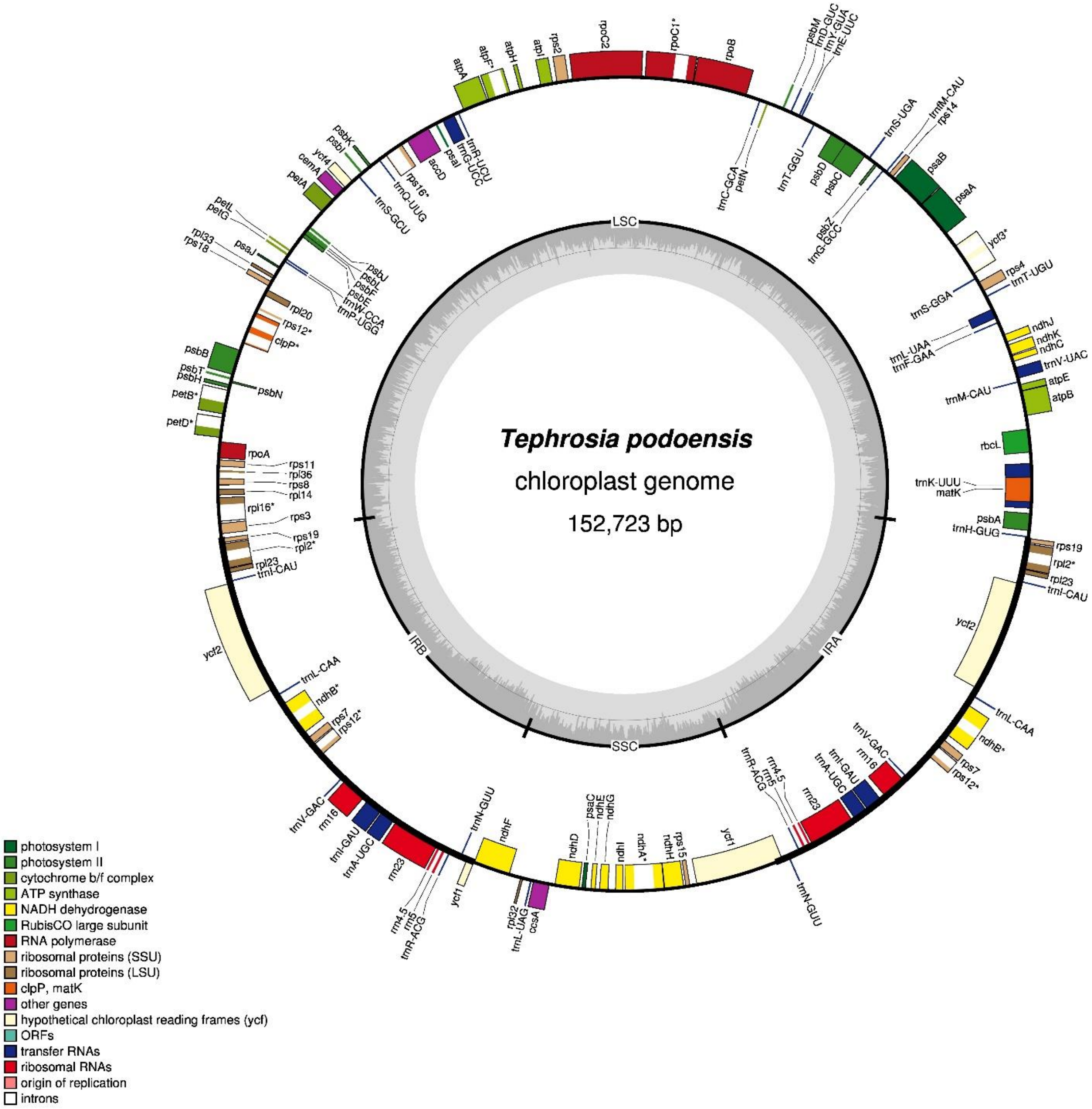

Tephrosia podoensis
chloroplast genome
152,723 bp
LSC
IRA
SSC
IRB
photosystem I
photosystem II
cytochrome b/f complex
ATP synthase
NADH dehydrogenase
RubisCO large subunit
RNA polymerase
ribosomal proteins (SSU)
ribosomal proteins (LSU)
clpP, matK
other genes
hypothetical chloroplast reading frames (ycf)
ORFs
transfer RNAs
ribosomal RNAs
origin of replication
introns

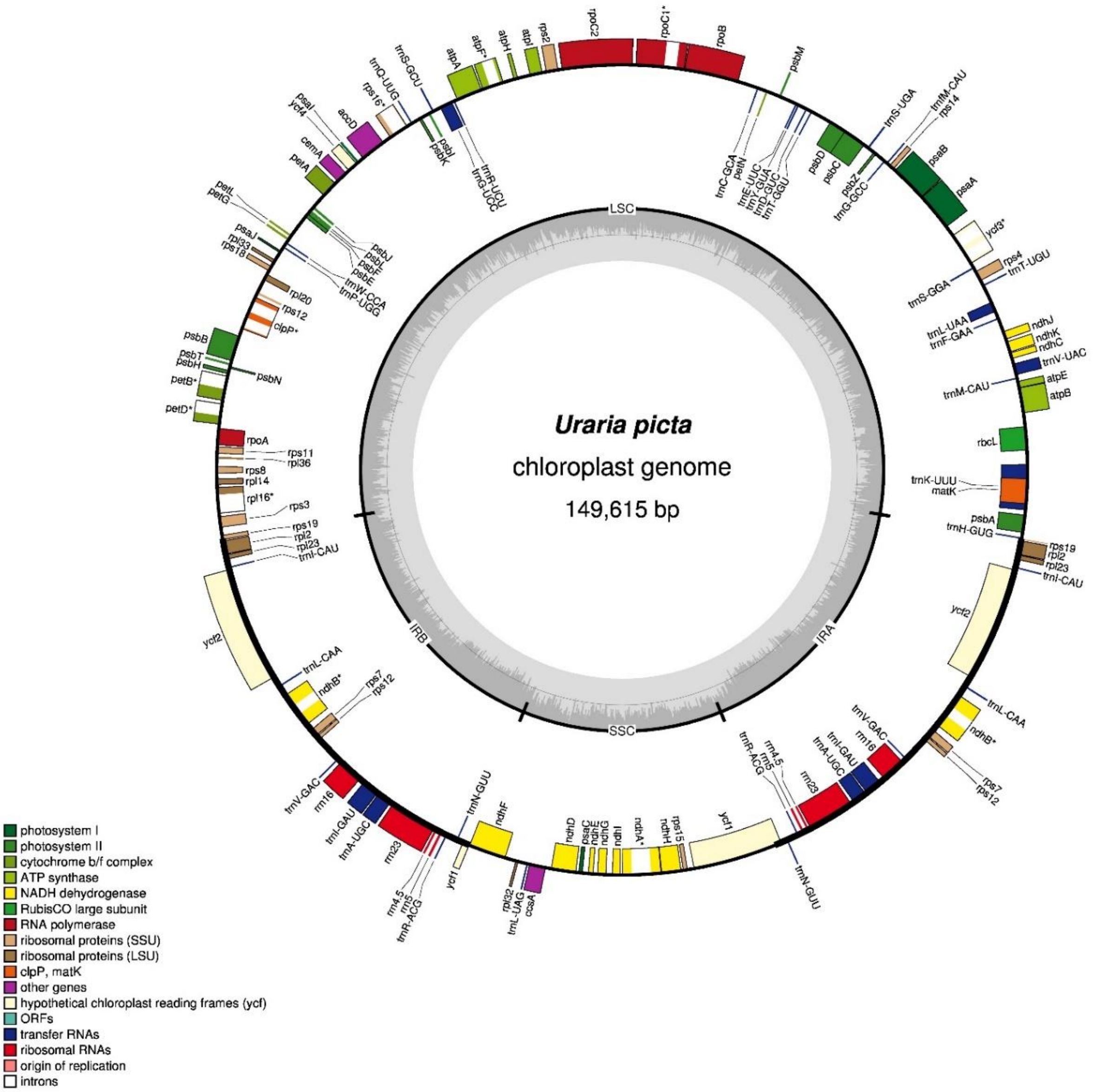

Uraria picta
chloroplast genome
149,615 bp
LSC
IRA
SSC
IRB
rpoC2
rpoC1*
rpoB
rps2
atpI
atpH
atpF*
atpA
trnS-GCU
trnQ-UUG
rps16*
accD
psaI
ycf4
cemA
petA
psbJ
psbL
psbF
psbE
trnW-CCA
trnP-UGG
petL
petG
psaJ
rpl33
rps18
rpl20
rps12
clpP*
psbB
psbT
psbH
psbN
petB*
petD*
rpoA
rps11
rpl36
rps8
rpl14
rpl16*
rps3
rps19
rpl2
rpl23
trnI-CAU
ycf2
trnL-CAA
ndhB*
rps7
trnV-GAC
rrn16
trnI-GAU
trnA-UGC
rrn23
rrn4.5
rrn5
trnR-ACG
ycf1
trnN-GUU
ndhF
rpl32
trnL-UAG
ccsA
ndhD
psaC
ndhE
ndhG
ndhI
ndhA*
ndhH
rps15
psbM
trnC-GCA
petN
trnE-UUC
trnY-GUA
trnD-GUC
trnT-GGU
psbD
psbC
psbZ
trnG-GCC
trnS-UGA
trnfM-CAU
rps14
psaB
psaA
ycf3*
rps4
trnT-UGU
trnS-GGA
trnL-UAA
trnF-GAA
ndhJ
ndhK
ndhC
trnV-UAC
trnM-CAU
atpE
atpB
rbcL
trnK-UUU
matK
psbA
trnH-GUG
psbI
psbK
trnR-UCU
trnG-UCC
photosystem I
photosystem II
cytochrome b/f complex
ATP synthase
NADH dehydrogenase
RubisCO large subunit
RNA polymerase
ribosomal proteins (SSU)
ribosomal proteins (LSU)
clpP, matK
other genes
hypothetical chloroplast reading frames (ycf)
ORFs
transfer RNAs
ribosomal RNAs
origin of replication
introns

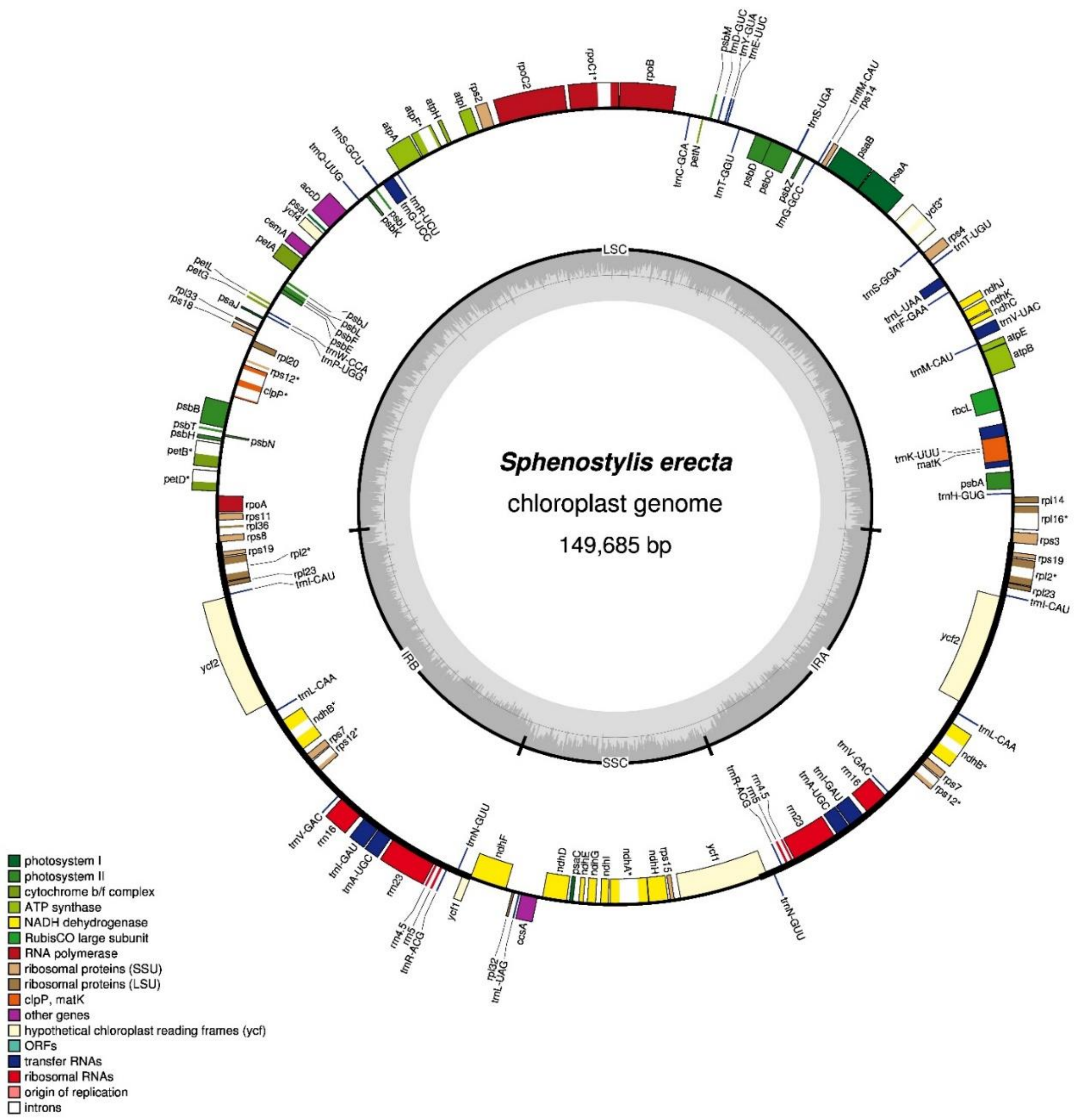
Sphenostylis erecta
chloroplast genome
149,685 bp
LSC
SSC
IRA
IRB
photosystem I
photosystem II
cytochrome b/f complex
ATP synthase
NADH dehydrogenase
RubisCO large subunit
RNA polymerase
ribosomal proteins (SSU)
ribosomal proteins (LSU)
clpP, matK
other genes
hypothetical chloroplast reading frames (ycf)
ORFs
transfer RNAs
ribosomal RNAs
origin of replication
introns

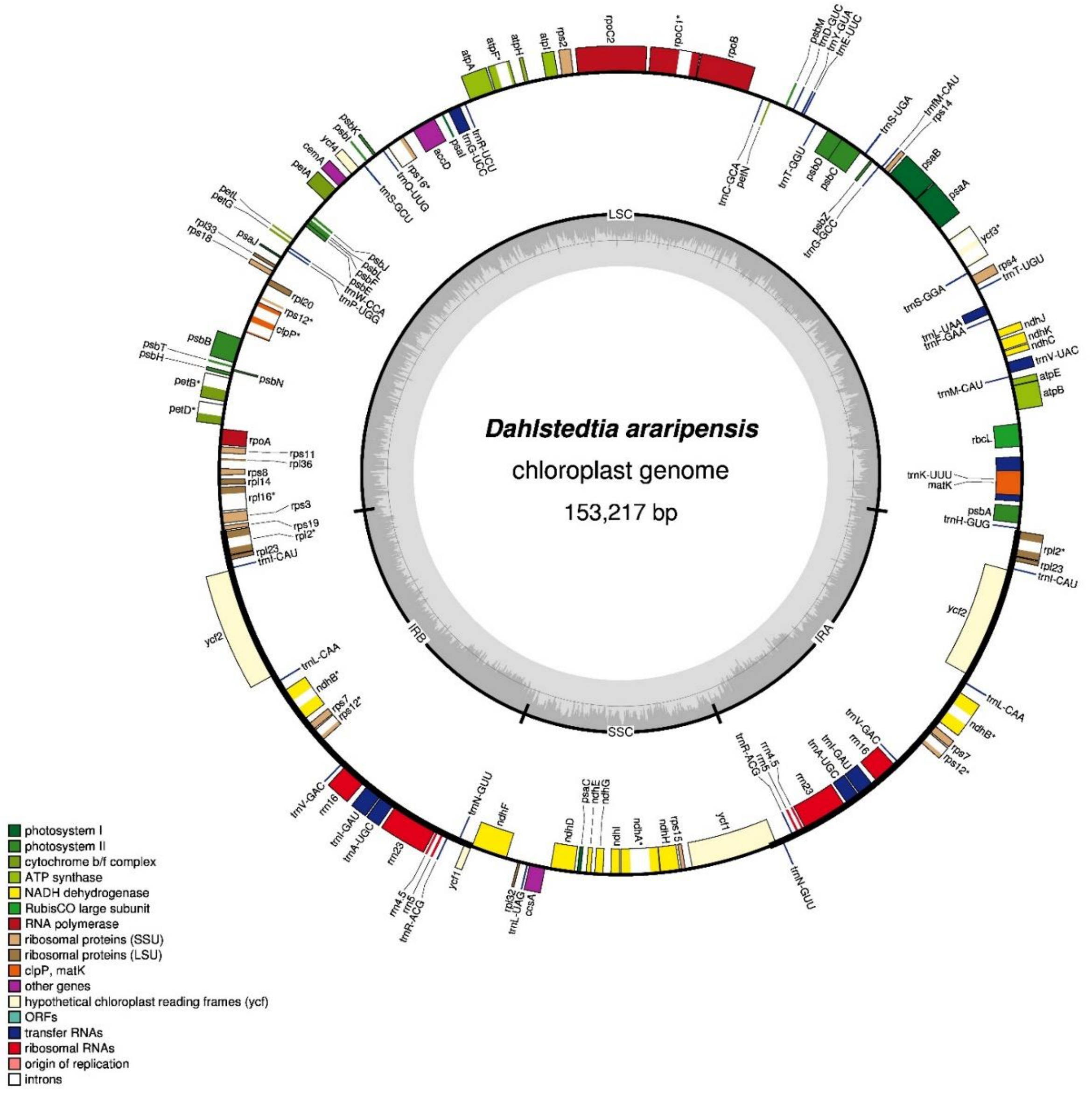

Dahlstedtia araripensis
chloroplast genome
153,217 bp
LSC
IRA
IRB
SSC
photosystem I
photosystem II
cytochrome b/f complex
ATP synthase
NADH dehydrogenase
RubisCO large subunit
RNA polymerase
ribosomal proteins (SSU)
ribosomal proteins (LSU)
clpP, matK
other genes
hypothetical chloroplast reading frames (ycf)
ORFs
transfer RNAs
ribosomal RNAs
origin of replication
introns

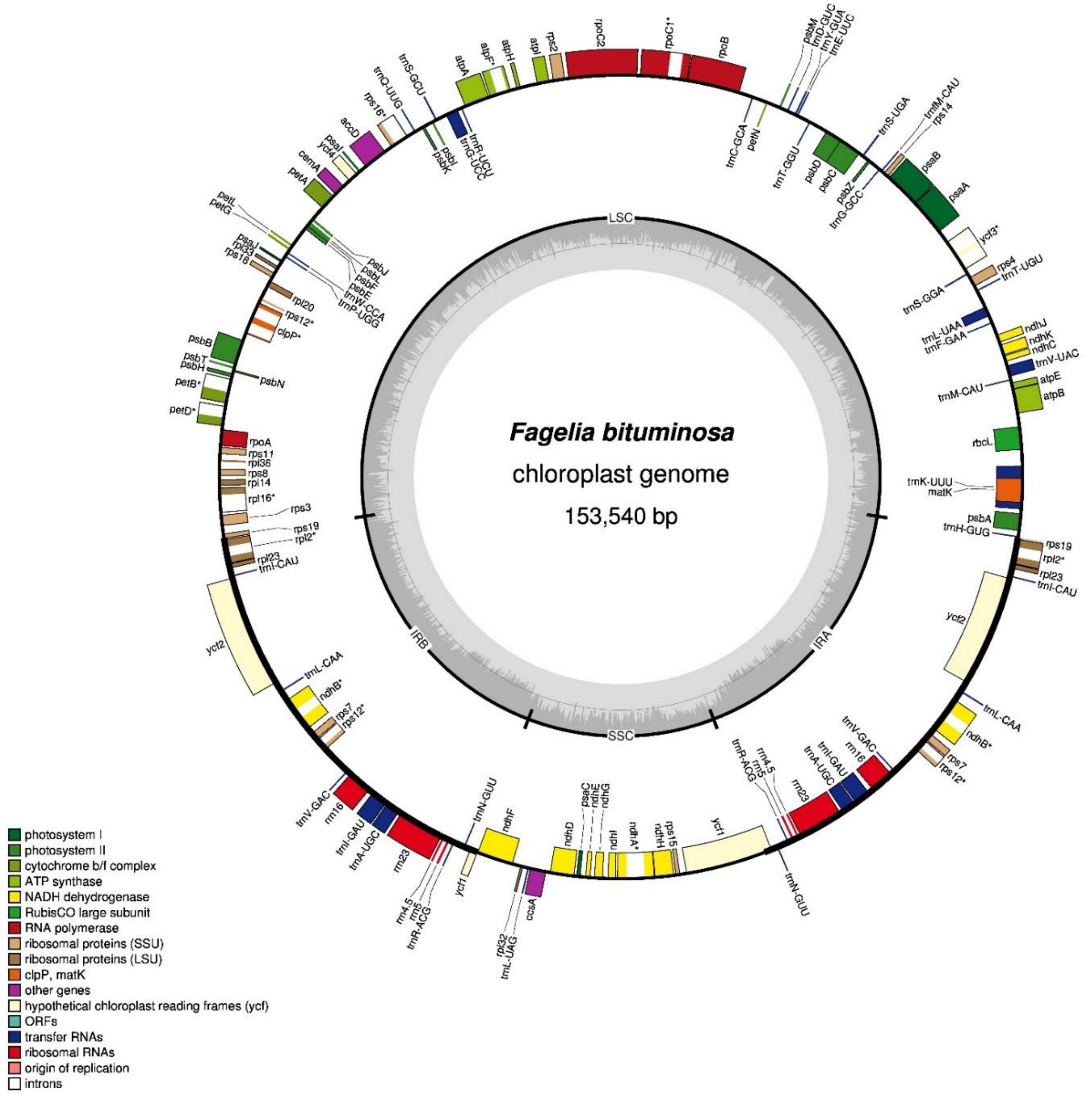

Fagelia bituminosa
chloroplast genome
153,540 bp
LSC
IRA
SSC
IRB
photosystem I
photosystem II
cytochrome b/f complex
ATP synthase
NADH dehydrogenase
RubisCO large subunit
RNA polymerase
ribosomal proteins (SSU)
ribosomal proteins (LSU)
clpP, matK
other genes
hypothetical chloroplast reading frames (ycf)
ORFs
transfer RNAs
ribosomal RNAs
origin of replication
introns

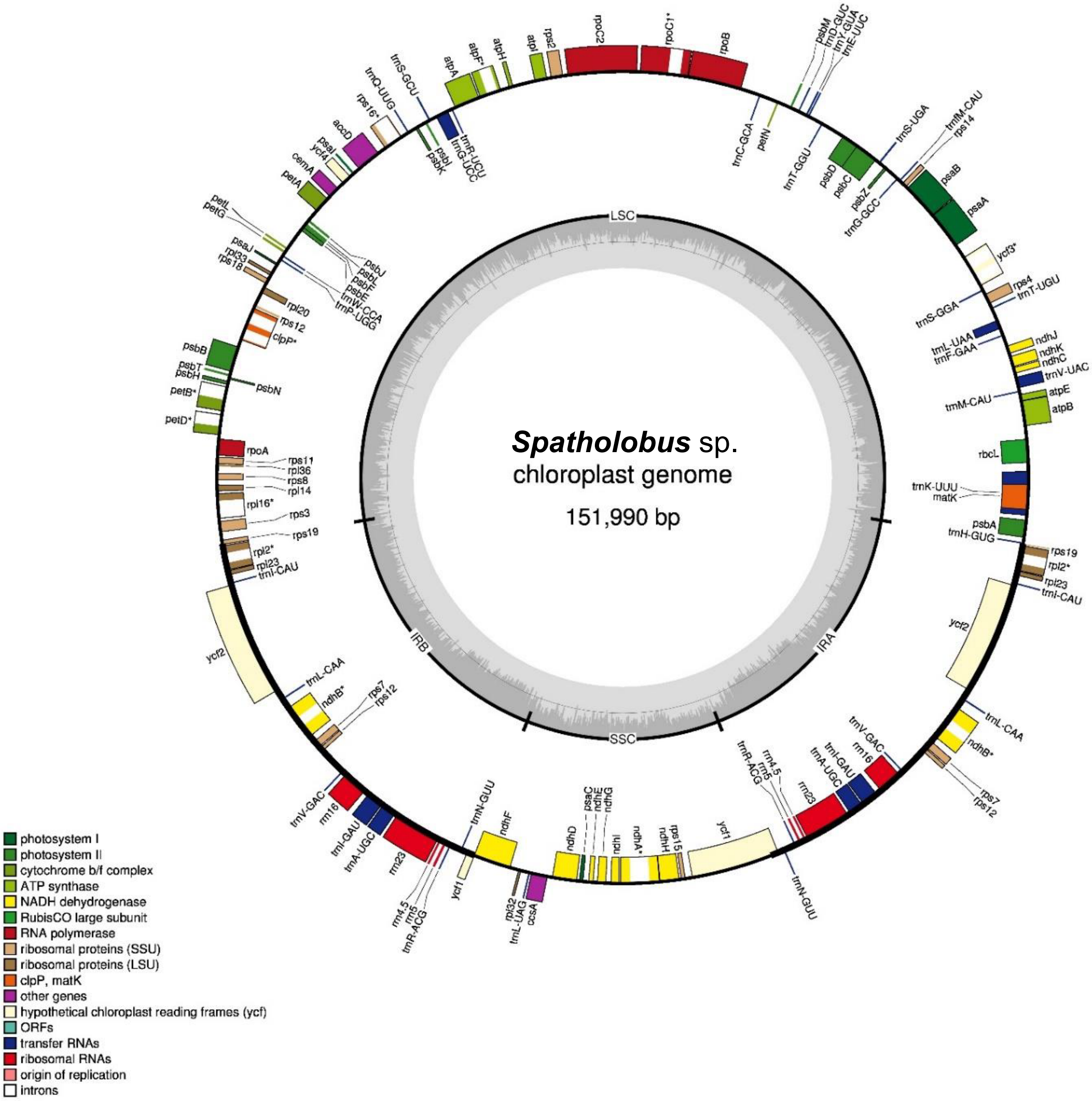
Spatholobus sp.
chloroplast genome
151,990 bp
LSC
IRA
SSC
IRB
photosystem I
photosystem II
cytochrome b/f complex
ATP synthase
NADH dehydrogenase
RubisCO large subunit
RNA polymerase
ribosomal proteins (SSU)
ribosomal proteins (LSU)
clpP, matK
other genes
hypothetical chloroplast reading frames (ycf)
ORFs
transfer RNAs
ribosomal RNAs
origin of replication
introns

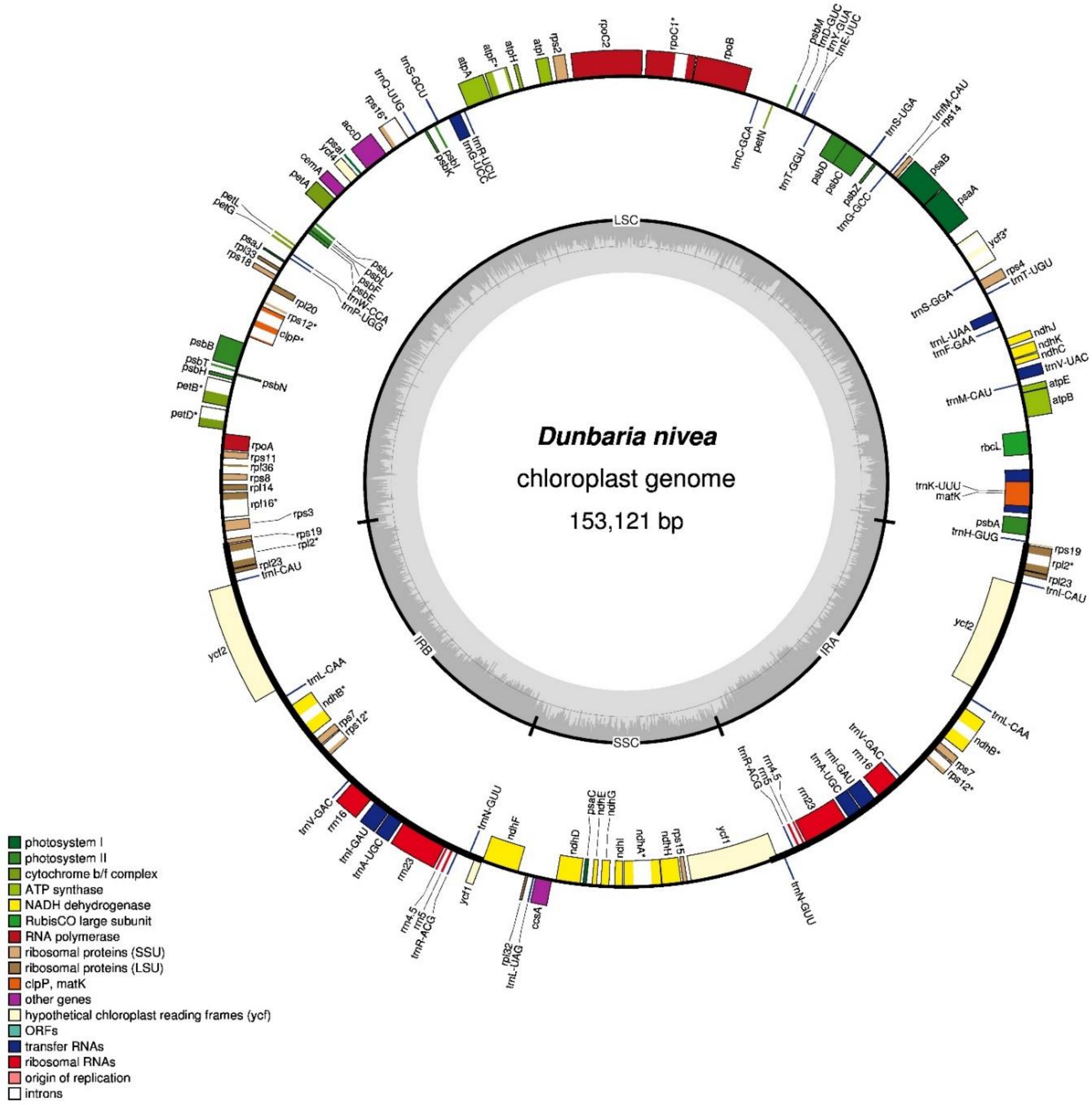

Dunbaria nivea
chloroplast genome
153,121 bp
LSC
IRA
SSC
IRB
photosystem I
photosystem II
cytochrome b/f complex
ATP synthase
NADH dehydrogenase
RubisCO large subunit
RNA polymerase
ribosomal proteins (SSU)
ribosomal proteins (LSU)
clpP, matK
other genes
hypothetical chloroplast reading frames (ycf)
ORFs
transfer RNAs
ribosomal RNAs
origin of replication
introns

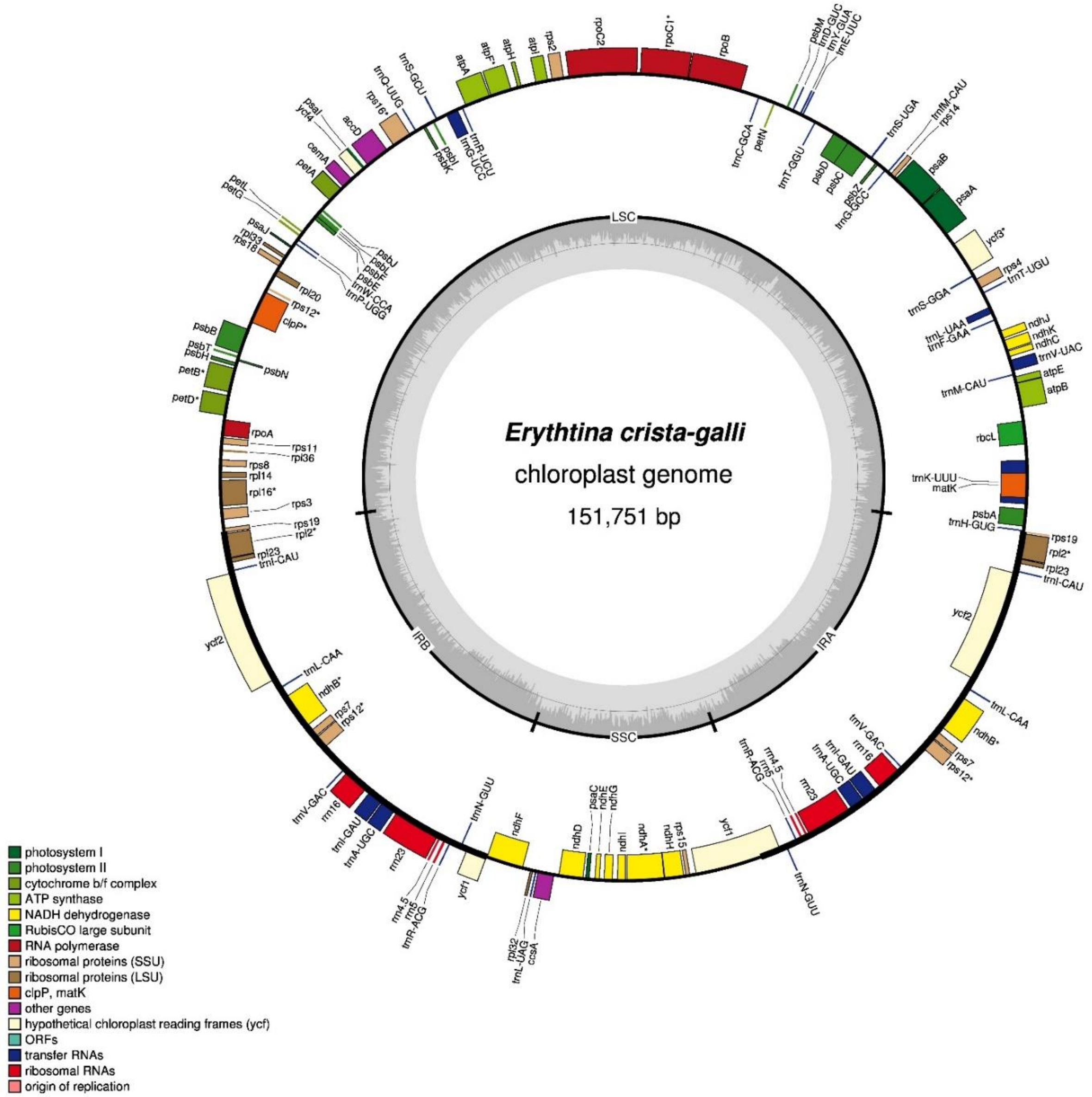

Erythtina crista-galli
chloroplast genome
151,751 bp
LSC
IRA
SSC
IRB
rpoC2
rpoC1*
rpoB
rps2
atpI
atpH
atpF*
atpA
trnS-GCU
trnQ-UUG
rps16*
psbI
psbK
trnR-UCU
trnG-UCC
accD
ycf4
psaI
cemA
petA
petL
petG
psaJ
rpl33
rps18
psbJ
psbL
psbF
psbE
trnW-CCA
trnP-UGG
rpl20
rps12*
clpP*
psbB
psbT
psbH
psbN
petB*
petD*
rpoA
rps11
rpl36
rps8
rpl14
rpl16*
rps3
rps19
rpl2*
rpl23
trnI-CAU
ycf2
trnL-CAA
ndhB*
rps7
rps12*
trnV-GAC
rrn16
trnI-GAU
trnA-UGC
rrn23
rrn4.5
rrn5
trnR-ACG
ycf1
trnN-GUU
ndhF
rpl32
trnL-UAG
ccsA
ndhD
psaC
ndhE
ndhG
ndhI
ndhA*
ndhH
rps15
psbM
trnD-GUC
trnY-GUA
trnE-UUC
trnC-GCA
petN
trnT-GGU
psbD
psbC
psbZ
trnG-GCC
trnS-UGA
trnfM-CAU
rps14
psaB
psaA
ycf3*
rps4
trnT-UGU
trnS-GGA
trnL-UAA
trnF-GAA
ndhJ
ndhK
ndhC
trnV-UAC
atpE
atpB
trnM-CAU
rbcL
trnK-UUU
matK
psbA
trnH-GUG
photosystem I
photosystem II
cytochrome b/f complex
ATP synthase
NADH dehydrogenase
RubisCO large subunit
RNA polymerase
ribosomal proteins (SSU)
ribosomal proteins (LSU)
clpP, matK
other genes
hypothetical chloroplast reading frames (ycf)
ORFs
transfer RNAs
ribosomal RNAs
origin of replication

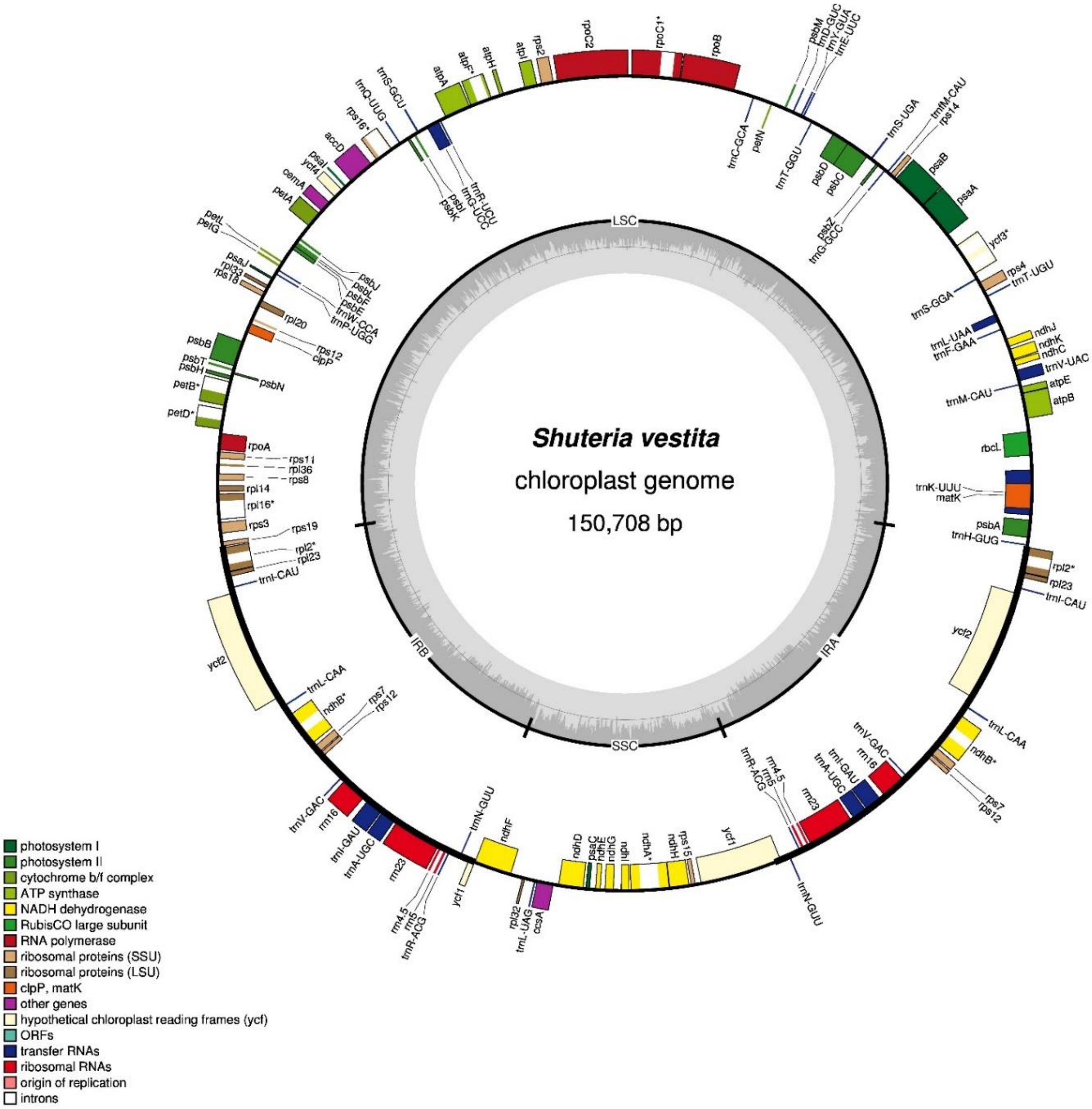

Shuteria vestita
chloroplast genome
150,708 bp
LSC
IRA
SSC
IRB
photosystem I
photosystem II
cytochrome b/f complex
ATP synthase
NADH dehydrogenase
RubisCO large subunit
RNA polymerase
ribosomal proteins (SSU)
ribosomal proteins (LSU)
clpP, matK
other genes
hypothetical chloroplast reading frames (ycf)
ORFs
transfer RNAs
ribosomal RNAs
origin of replication
introns

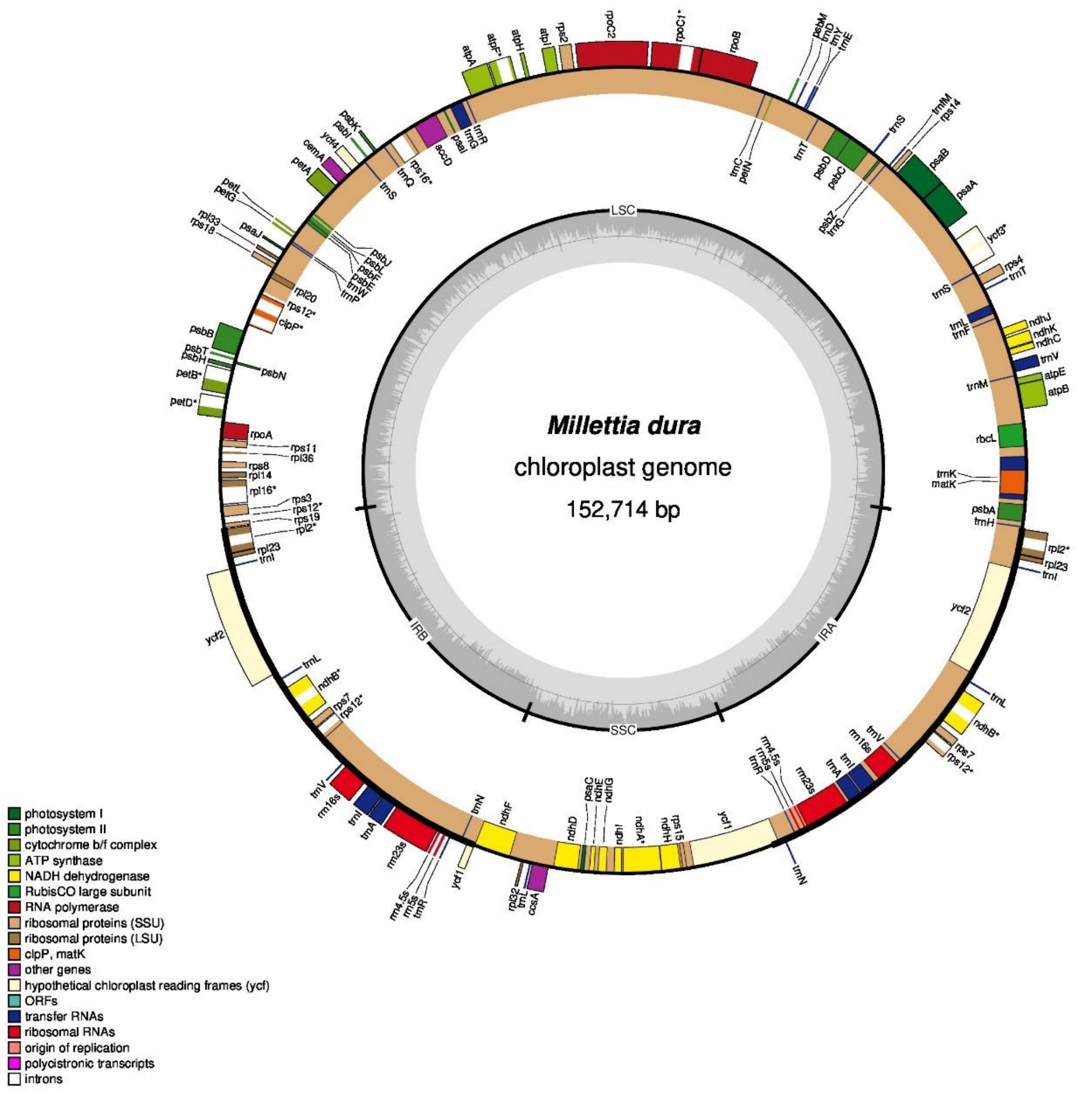

Supplementary Figure S1 Gene maps of the 35 sequenced Millettioid/Phaseoloid clade plastomes. Thick lines in the red area indicate the extent of the inverted repeat regions which separate the genome into small and large single-copy regions. Genes belonging to different functional groups are colour-coded. The dark grey in the inner circle represents the GC content, and the light grey corresponds to the AT content.
