# Supplementary material for: New Insights Into the Plastome Evolution of the Millettioid/Phaseoloid Clade (Papilionoideae, Leguminosae)
Source: Front Plant Sci. 2020 Mar 10;11:151. doi: 10.3389/fpls.2020.00151 (PMC7076112; doi:10.3389/fpls.2020.00151)
Supplement: Supplementary file 1 [file Presentation_1.zip › Supplementary/Figure S3 Mauve alignment showing the inversions.PDF]

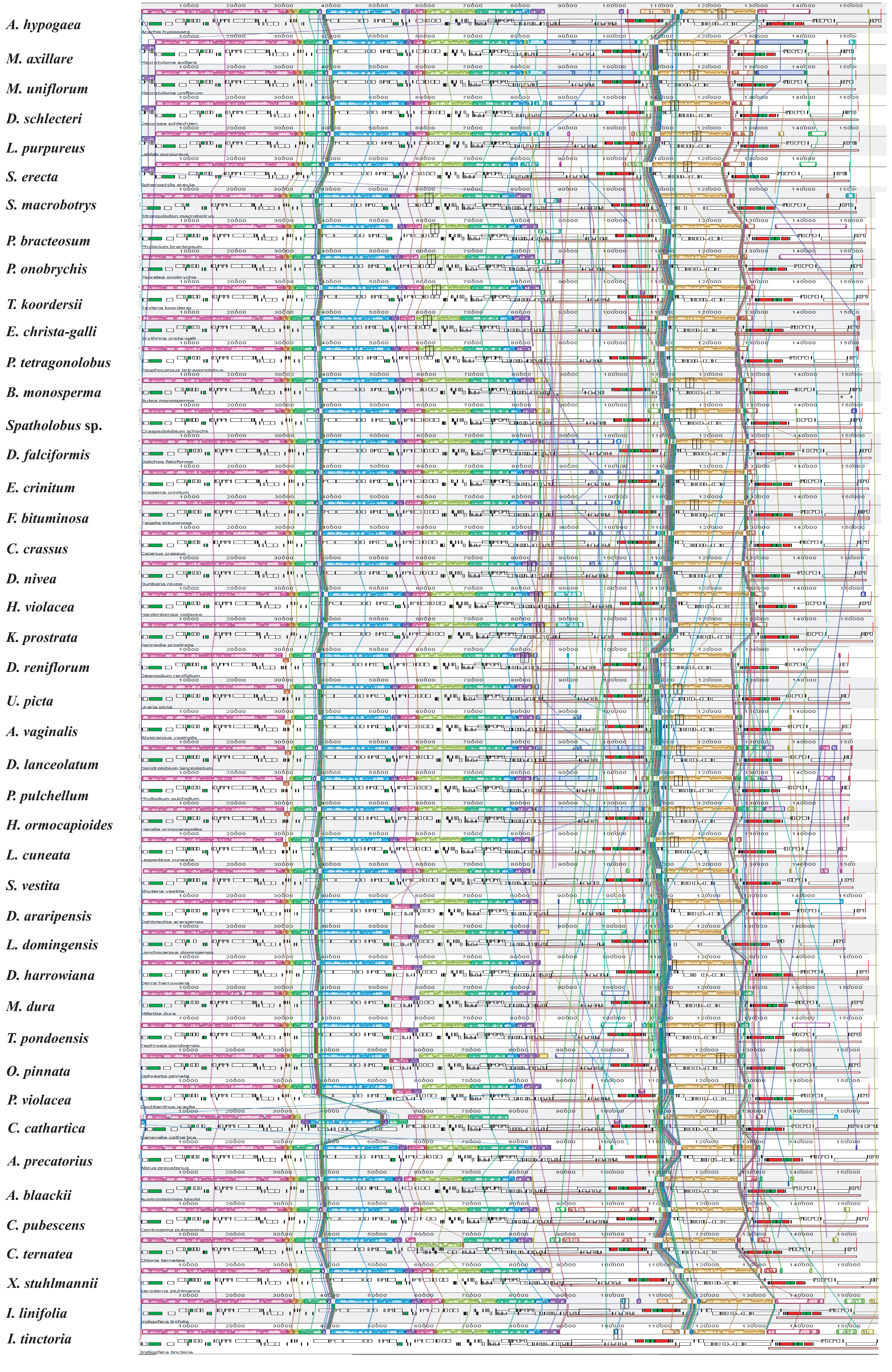
A. hypogaea
M. axillare
M. uniflorum
D. schlecteri
L. purpureus
S. erecta
S. macrobotrys
P. bracteosum
P. onobrychis
T. koordersii
E. christa-galli
P. tetragonolobus
B. monosperma
Spatholobus sp.
D. falciformis
E. crinitum
F. bituminosa
C. crassus
D. nivea
H. violacea
K. prostrata
D. reniflorum
U. picta
A. vaginalis
D. lanceolatum
P. pulchellum
H. ormocapioides
L. cuneata
S. vestita
D. araripensis
L. domingensis
D. harrowiana
M. dura
T. pondoensis
O. pinnata
P. violacea
C. cathartica
A. precatorius
A. blaackii
C. pubescens
C. ternatea
X. stuhlmannii
I. linifolia
I. tinctoria
