# Supplementary material for: New Insights Into the Plastome Evolution of the Millettioid/Phaseoloid Clade (Papilionoideae, Leguminosae)
Source: Front Plant Sci. 2020 Mar 10;11:151. doi: 10.3389/fpls.2020.00151 (PMC7076112; doi:10.3389/fpls.2020.00151)
Supplement: Supplementary file 1 [file Presentation_1.zip › Supplementary/Table S1.DOCX]

**Table 1.** **GenBank accession numbers of the species from the MP clade studied**

| SN | Species | Accession NO. |
| --- | --- | --- |
|  | *Abrus precatorius** | MN709888 |
|  | *Alysicarpus vaginalis* | MN734440 |
|  | *Austrosteenisia blackii* | MN936097 |
|  | *Cajanus crassus* | MN936098 |
|  | *Butea monosperma** | MN709868 |
|  | *Canavalia cathartica** | MN709790 |
|  | *Centrosema pubescens* | MN936099 |
|  | *Clitoria ternatea** | MN709849 |
|  | *Philenoptera violacea* | MN936100 |
|  | *Spatholobus* sp*.* | MN936101 |
|  | *Dahlstedtia araripensis* | MN966622 |
|  | *Decorsea schlechteri* | MN966623 |
|  | *Dendrolobium lanceolatum* | MN966624 |
|  | *Derris harrowiana* | MN966625 |
|  | *Desmodium renifolium** | MN709854 |
|  | *Dolichos falciformis* | MN966626 |
|  | *Dunbaria nivea* | MN966627 |
|  | *Eriosema crinitum* | MN966628 |
|  | *Erythrina crista-galli* | MN966629 |
|  | *Fagelia bituminosa* | MN966630 |
|  | *Hanslia ormocarpoides* | MN966631 |
|  | *Hardenbergia violacea* | MN966632 |
|  | *Indigofera linifolia** | MN709836 |
|  | *Indigofera tinctoria** | NC026680 |
|  | *Kennedia prostrata* | MN966633 |
|  | *Lablab purpureus* | MN966634 |
|  | *Lespedeza cuneata* | MN966635 |
|  | *Lonchocarpus domingensis* | MN966636 |
|  | *Macrotyloma axillare* | MN966637 |
|  | *Macrotyloma uniflorum* | MN966638 |
|  | *Millettia dura* | MN966639 |
|  | *Ophrestia pinnata* | MN966640 |
|  | *Phylacium bracteosum* | MN966641 |
|  | *Phyllodium pulchellum* | MN966642 |
|  | *Psoralea onobrychis** | MN709873 |
|  | *Psophocarpus tetragonolobus* | MN966643 |
|  | *Shuteria vestita* | MN966644 |
|  | *Sphenostylis erecta* | MN966645 |
|  | *Strongylodon macrobotrys* | MN966646 |
|  | *Tephrosia pondoensis* | MN966647 |
|  | *Uraria picta* | MN966649 |
|  | *Teyleria koordersii* | MN966648 |
|  | *Xeroderris stuhlmannii* | MN966650 |
|  | **Outgroups** |  |
| O | *Podalyria calyptrata* | MN966651 |
|  | *Pterocarpus violaceus* | MN966652 |
|  | *Kotschya aeschynomenoides* | MN966653 |
|  | *Parochetus communis* | MN966654 |
|  | *Ceratonia siliqua** | NC026678 |
|  | *Tamarindus indica** | NC026685 |

** Sequences previously published*
