# Supplementary material for: New Insights Into the Plastome Evolution of the Millettioid/Phaseoloid Clade (Papilionoideae, Leguminosae)
Source: Front Plant Sci. 2020 Mar 10;11:151. doi: 10.3389/fpls.2020.00151 (PMC7076112; doi:10.3389/fpls.2020.00151)
Supplement: Supplementary file 1 [file Presentation_1.zip › Supplementary/Table S2.DOCX]

**Table S2.** Summary of plastome for 43 species of the MP clade

| Tribe | Species | Length  (bp) | LSC (bp) | IR (bp) | SSC (bp) | GC (%) | Coverage (x) | Total  genes | PCGs/ CDS | tRNA gene | rRNA gene |
| --- | --- | --- | --- | --- | --- | --- | --- | --- | --- | --- | --- |
| Abreae | *Abrus precatorius* | 156,500 | 86,540 | 25,773 | 18,414 | 35.4 | 748.8 | 130(111) | 83 (77) | 37(30) | 8(4) |
| Desmodieae | *Alysicarpus vaginalis* | 149,890 | 83,314 | 24,187 | 18,202 | 35.1 | 952.99 | 130(111) | 83(77) | 37(30) | 8(4) |
| Desmodieae | *Dendrolobium lanceolatum* | 150,186 | 83,222 | 24,292 | 18,380 | 35 | 733.46 | 130(111) | 83(77) | 37(30) | 8(4) |
| Desmodieae | *Desmodium renifolium* | 149,204 | 82,925 | 24,111 | 18,387 | 35.3 | 1,590.3 | 130(111) | 83(77) | 37(30) | 8(4) |
| Desmodieae | *Hanslia ormocarpoides* | 149,454 | 83,930 | 23,489 | 18,546 | 35.1 | 418.85 | 129(111) | 82(75) | 37(30) | 8(4) |
| Desmodieae | *Hardenbergia violacea* | 154,056 | 85,429 | 25,137 | 18,343 | 35.1 | 1406.52 | 131(111) | 83(77) | 37(30) | 8(4) |
| Desmodieae | *Kennedia prostrata* | 154,390 | 85,802 | 25,120 | 18,348 | 35.1 | 831.99 | 130(111) | 83(77) | 37(30) | 8(4) |
| Desmodieae | *Lespedeza cuneata* | 149,029 | 82,433 | 23,832 | 18,932 | 35 | 648.34 | 129(111) | 83(77) | 37(30) | 8(4) |
| Desmodieae | *Phyllodium pulchellum* | 149,717 | 82,725 | 24,294 | 18,404 | 35.1 | 1,284.18 | 130(111) | 83(77) | 37(30) | 8(4) |
|  |  |  |  |  |  |  |  |  |  |  |  |
| Desmodieae | *Uraria picta* | 149,615 | 82,956 | 24,127 | 18,405 | 35.2 | 958.92 | 130(111) | 83(77) | 37(30) | 8(4) |
| Indigfereae | *Indigofera linifolia* | 160,040 | 90,459 | 25,323 | 18,935 | 35.8 | 1,168.2 | 130(111) | 83(77) | 37(30) | 8(4) |
| Indigoferae | *Indigofera tinctoria* | 158,367 | 88,852 | 25,358 | 18,799 | 35.8 | NA | 129(111) | 83(77) | 37(30) | 8(4) |
| Millettieae | *Austrosteenisia blackii* | 152,551 | 83,176 | 25,447 | 18,481 | 35 | 680.01 | 129(110) | 82(76) | 37(30) | 8(4) |
| Millettieae | *Dahlstedtia araripensis* | 153,217 | 83,571 | 25,546 | 18,554 | 34.8 | 430.13 | 129(111) | 83(77) | 37(30) | 8(4) |
| Millettieae | *Derris harrowiana* | 153,846 | 84,436 | 25,531 | 18,348 | 34.7 | 787.9 | 130(111) | 82(75) | 37(30) | 8(4) |
| Millettieae | *Lonchocarpus domingensis* | 148,889 | 83,509 | 24,084 | 17,202 | 35 | 477.23 | 126(110) | 73(68) | 37(30) | 8(4) |
| Millettieae | *Millettia dura* | 152,714 | 83,363 | 25,487 | 18,377 | 34.9 | 373.98 | 129(110) | 83(77) | 37(30) | 8(4) |
| Millettieae | *Tephrosia pondoensis* | 152,753 | 82,757 | 25,756 | 18,454 | 34.5 | 576.27 | 130(111) | 84(77) | 37(30) | 8(4) |
| Millettieae | *Xeroderris stuhlmannii* | 157,009 | 87,141 | 25,642 | 18,584 | 35.4 | 367.2 | 129(111) | 83(77) | 37(30) | 8(4) |
| Millettieae | *Philenoptera violacea* | 154,445 | 85,022 | 25,592 | 18,239 | 35.2 | 162 | 129(111) | 83(77) | 37(30) | 8(4) |
| Phaseoleae | *Butea monosperma* | 151,874 | 83,541 | 25,084 | 18,165 | 35.3 | 643.1 | 130(111) | 83(77) | 37(30) | 8(4) |
| Phaseoleae | *Cajanus crassus* | 153,549 | 84,224 | 25,764 | 17,797 | 35 | 1,536.1 | 130(111) | 83(77) | 37(30) | 8(4) |
| Phaseoleae | *Canavalia cathartica* | 158,223 | 77,970 | 30,644 | 18,965 | 34.6 | 248.5 | 136(111) | 90(77) | 37(30) | 8(4) |
| Phaseoleae | *Centrosema pubescens* | 154,141 | 84,469 | 25,479 | 18,714 | 34.8 | 514.76 | 129(111) | 81(75) | 37(30) | 8(4) |
| Phaseoleae | *Clitoria ternatea* | 151,673 | 83,316 | 24,977 | 18,403 | 34.6 | 824.8 | 129(110) | 81(75) | 37(30) | 8(4) |
| Phaseoleae | *Decorsea schlechteri* | 151,540 | 81,258 | 26,426 | 17,430 | 35.3 | 874.58 | 129(110) | 82(75) | 37(30) | 8(4) |
| Phaseoleae | *Dolichos falciformis* | 153,884 | 84,625 | 25,722 | 17,815 | 34.2 | 452.34 | 130(111) | 83(77) | 37(30) | 8(4) |
| Phaseoleae | *Dunbaria nivea* | 153,121 | 83,977 | 25,722 | 17,700 | 35.1 | 180.65 | 130(111) | 83(77) | 37(30) | 8(4) |
| Phaseoleae | *Eriosema crinitum* | 153,403 | 84,234 | 25,687 | 17,795 | 35.2 | 285.64 | 130(111) | 82(75) | 37(30) | 8(4) |
| Phaseoleae | *Erythrina crista-galli* | 151,751 | 82,389 | 26,303 | 16,756 | 35.3 | 993.42 | 130(111) | 83(77) | 37(30) | 8(4) |
| Phaseoleae | *Fagelia bituminosa* | 153,540 | 84,209 | 25,737 | 17,853 | 35.2 | 660.33 | 130(111) | 83(77) | 37(30) | 8(4) |
| Phaseoleae | *Lablab purpureus* | 151,709 | 80,914 | 26,437 | 18,031 | 35.4 | 1,579.9 | 130(111) | 82(75) | 37(30) | 8(4) |
| Phaseoleae | *Macrotyloma axillare* | 151,039 | 80,487 | 26,428 | 17,696 | 35.3 | 1,083.96 | 129(110) | 82(75) | 37(30) | 8(4) |
| Phaseoleae | *Macrotyloma uniflorum* | 151,012 | 80,436 | 26,433 | 17,710 | 35.3 | 1,078.28 | 129(110) | 82(75) | 37(30) | 8(4) |
| Phaseoleae | *Ophrestia pinnata* | 152,532 | 83,661 | 25,455 | 17,961 | 34.5 | 313.99 | 129(110) | 83(77) | 37(30) | 8(4) |
| Phaseoleae | *Phylacium bracteosum* | 153,106 | 83,969 | 25590 | 17,957 | 35.5 | 341.6 | 128(109) | 82(75) | 36(29) | 8(4) |
| Phaseoleae | *Psophocarpus tetragonolobus* | 151,571 | 82,735 | 25,530 | 17,776 | 35.3 | 671.17 | 130(111) | 83(77) | 37(30) | 8(4) |
| Phaseoleae | *Spatholobus* sp. | 151,990 | 83,942 | 24,984 | 18,080 | 35.4 | 555.07 | 130(111) | 83(77) | 37(30) | 8(4) |
| Phaseoleae | *Sphenostylis erecta* | 149,685 | 79,202 | 26,393 | 17,697 | 35.2 | 902 | 129(111) | 82(75) | 37(30) | 8(4) |
| Phaseoleae | *Strongylodon macrobotrys* | 151,063 | 81,990 | 27,102 | 14,869 | 34.9 | 1,069.54 | 130(111) | 75(68) | 37(30) | 8(4) |
| Phaseoleae | *Shuteria vestita* | 150,708 | 82,821 | 24,890 | 18,107 | 35.7 | 702.74 | 129(111) | 83(77) | 37(30) | 8(4) |
| Phaseoleae | *Teyleria koordersii* | 153,632 | 84,451 | 25,579 | 18,023 | 35.4 | 336.51 | 130(111) | 83(77) | 37(30) | 8(4) |
| Psoraleeae | *Psoralea onobrychis* | 152,378 | 83,349 | 25,569 | 17,891 | 35.5 | 1,207.5 | 130(111) | 83(77) | 37(30) | 8(4) |

Note: Figures in brackets are the number of unique genes
