# Supplementary material for: New Insights Into the Plastome Evolution of the Millettioid/Phaseoloid Clade (Papilionoideae, Leguminosae)
Source: Front Plant Sci. 2020 Mar 10;11:151. doi: 10.3389/fpls.2020.00151 (PMC7076112; doi:10.3389/fpls.2020.00151)
Supplement: Supplementary file 1 [file Presentation_1.zip › Supplementary/Table S3.DOCX]

**Table S3.** Summary of partitioning scheme for the IQ-tree and BI analysis

**Table S3A: Details of CDs data matrix**

Scheme lnL: -463625.02044677734

Scheme AICc: 928333.678933

Number of params: 538

Number of sites: 76470

Number of subsets: 41

| Subset | Partition names | Best Model | Best-fit Model | LogL | AICc | BIC | Unique | Infor | Invar | rates |
| --- | --- | --- | --- | --- | --- | --- | --- | --- | --- | --- |
| 1 | *accD, rpl20* | GTR+I+G | TVM+F+I+G4 | -18432.4036 | 36884.8897 | c + 0.0000 | 1123 | 667 | 1620 | invgamma |
| 2 | *atpA, atpB* | GTR+I+G | TVM+F+I+G4 | -16022.9293 | 32065.9307 | 32126.1104 | 721 | 519 | 2222 | invgamma |
| 3 | *atpE, rps16, rps4, rpl14, rps2, rpoC1* | GTR+I+G | GTR+F+I+G | -25733.7660 | 51489.5900 | 51560.2005 | 1262 | 929 | 3008 | invgamma |
| 4 | *atpF* | GTR+I+G | K3Pu+F+I+G4 | -3809.1713 | 7634.5723 | 7669.9842 | 232 | 145 | 409 | invgamma |
| 5 | *atpH, petB* | GTR+I+G | TPM3u+F+I+G4 | -4332.9555 | 8682.0736 | 8720.2766 | 188 | 153 | 701 | invgamma |
| 6 | *atpI, ndhJ, ndhC, ndhE, psbT* | GTR+I+G | TVM+F+I+G4 | -10549.5796 | 21119.2700 | 21175.1583 | 557 | 370 | 1415 | invgamma |
| 7 | *ccsA* | GTR+I+G | TVM+F+I+G4 | -8526.5795 | 17073.3694 | 17122.7813 | 504 | 313 | 575 | invgamma |
| 8 | *cemA* | GTR+G | TVM+F+G4 | -5186.7341 | 10391.7239 | 10432.6061 | 296 | 190 | 409 | gamma |
| 9 | *clpP* | GTR+G | TPM3u+F+G4 | -5188.1392 | 10390.4685 | 10421.0218 | 287 | 182 | 268 | gamma |
| 10 | *matK* | GTR+I+G | TVM+F+G4 | -14721.0267 | 29460.1659 | 29508.5149 | 870 | 598 | 715 | invgamma |
| 11 | *ndhA, petL, ndhD* | GTR+I+G | GTR+F+I+G4 | -17970.3098 | 35962.7180 | 36027.5103 | 853 | 636 | 1723 | invgamma |
| 12 | *ndhB* | GTR+I+G | TVM+F+G4 | -4232.4022 | 8482.9269 | 8530.4965 | 185 | 103 | 1260 | invgamma |
| 13 | *ndhF* | GTR+I+G | TVM+F+I+G4 | -20836.2983 | 41692.6901 | 41750.2649 | 1000 | 748 | 1271 | invgamma |
| 14 | *ndhG, psaI* | GTR+I+G | TVM+F+G4 | -4137.2211 | 8292.7253 | 8332.6794 | 262 | 163 | 389 | invgamma |
| 15 | *ndhH* | GTR+I+G | TPM3u+F+I+G4 | -6927.6300 | 13871.3821 | 13911.9002 | 346 | 240 | 819 | invgamma |
| 16 | *ndhI, ndhK* | GTR+I+G | TPM2u+F+I+G4 | -7814.9005 | 15645.9157 | 15686.9437 | 458 | 273 | 827 | invgamma |
| 17 | *rps14, psbH, rpoB, petA* | GTR+I+G | TVM+F+I+G4 | -27350.9529 | 54721.9522 | 54786.5817 | 1232 | 979 | 3172 | invgamma |
| 18 | *psbB, psbI, petD* | GTR+I+G | GTR+F+I+G4 | -10420.1503 | 20862.4252 | 20924.6032 | 467 | 341 | 1606 | invgamma |
| 19 | *psbC, psaC, petG* | GTR+I+G | K3Pu+F+I+G4 | -7764.3040 | 15544.6893 | 15588.4920 | 357 | 240 | 1379 | invgamma |
| 20 | *petN, psbM, psbZ* | GTR+G | TPM3u+F+G4 | -1678.2431 | 3370.7816 | 3398.1951 | 54 | 285 | 285 | gamma |
| 21 | *psaB, psaA* | GTR+I+G | TVM+F+I+G4 | -20918.1967 | 41856.4429 | 41920.4180 | 854 | 685 | 3399 | invgamma |
| 22 | *psaJ, psbK* | GTR+I+G | K3Pu+F+I+G4 | -1881.0814 | 3778.6244 | 3808.3344 | 106 | 62 | 218 | invgamma |
| 23 | *psbL, psbD, psbA* | GTR+I+G | K3Pu+F+I+G4 | -8694.2040 | 17404.4725 | 17450.1254 | 362 | 253 | 1835 | invgamma |
| 24 | *psbJ, psbF, psbE* | GTR+I+G | TVM+F+I+G4 | -1716.9154 | 3454.2854 | 3495.8764 | 89 | 48 | 406 | invgamma |
| 25 | *rps12, ycf3, psbN* | GTR+I+G | K3Pu+F+I+G4 | -4103.0139 | 8222.1699 | 8261.4718 | 210 | 127 | 804 | invgamma |
| 26 | *rbcL* | GTR+I+G | TIM+F+I+G4 | -7708.6693 | 15435.4648 | 15482.7714 | 336 | 238 | 1094 | invgamma |
| 27 | *rpl16, rpl36, rps3* | GTR+I+G | TVM+F+I+G4 | -8182.7925 | 16385.7705 | 16436.4608 | 428 | 271 | 743 | invgamma |
| 28 | *rpl23* | GTR+G | TPM2u+F+G4 | -959.6794 | 1933.7423 | 1959.2852 | 63 | 23 | 247 | gamma |
| 29 | *rpl2, rrn4.5, rps7* | GTR+G | K3Pu+F+G4 | -4492.7203 | 8999.5208 | 9036.1651 | 224 | 116 | 1152 | gamma |
| 30 | *rpl32* | GTR+I+G | GTR+F+I+G4 | -1586.3709 | 3196.3322 | 3229.7415 | 97 | 58 | 94 | invgamma |
| 31 | *rps8, rps11, rpl33* | GTR+G | TVM+F+G4 | -7776.7398 | 15571.6451 | 15616.4909 | 461 | 281 | 610 | gamma |
| 32 | *rpoA* | GTR+I+G | TVM+F+I+G4 | -7070.6287 | 14161.4728 | 14210.6498 | 403 | 254 | 601 | invgamma |
| 33 | *rpoC2* | GTR+I+G | GTR+F+I+G4 | -33307.1416 | 66636.3434 | 66706.5486 | 1749 | 1295 | 2355 | invgamma |
| 34 | *rps15* | GTR+G | TVM+F+G4 | -2363.8176 | 4746.2826 | 4778.6018 | 155 | 84 | 143 | gamma |
| 35 | *rps18* | GTR+I+G | HKY+F+I+G4 | -2781.1790 | 5576.6395 | 5604.4025 | 190 | 87 | 231 | invgamma |
| 36 | *rps19* | GTR+I+G | TIM2+F+G4 | -1593.5886 | 3203.6990 | 3232.3972 | 93 | 54 | 196 | invgamma |
| 37 | *rrn5, rrn16* | GTR+I+G | HKY+F+I | -3150.6297 | 6313.3116 | 6345.5856 | 83 | 34 | 1528 | invgamma |
| 38 | *rrn23* | GTR+I+G | TIM+F+I+G4 | -6711.0367 | 13440.1366 | 13493.6880 | 232 | 124 | 2597 | invgamma |
| 39 | *ycf1* | GTR+I+G | TVM+F+I+G4 | -75916.3811 | 151852.7941 | 151921.1607 | 4000 | 2709 | 2763 | invgamma |
| 40 | *ycf2* | GTR+I+G | TVM+F+G4 | -31852.0607 | 63722.1459 | 63784.2582 | 1656 | 1057 | 5162 | invgamma |
| 41 | *ycf4* | GTR+I+G | TVM+F+I+G4 | -9395.1757 | 18810.6763 | 18855.6893 | 510 | 365 | 200 | invgamma |

AICc: Corrected AIC scores, BIC: Bayesian information criterion scores, Unique: Number of unique site patterns, Infor: Number of parsimony-informative sites, Invar: Number of invariant sites

**Table S3B: Details of NCDs data matrix**

Scheme lnL: -709963.3389892578

Scheme AICc: 1421525.46674

Number of params: 793

Number of sites: 99262

Number of subsets: 67

| Subset | Partition names | Best Model | Best-fit Model | LogL | AICc | BIC | Unique | Infor | Invar | rates |
| --- | --- | --- | --- | --- | --- | --- | --- | --- | --- | --- |
| 1 | trnG-UCC exon1 - trnS-GCU, psbI-trnS-GCU, accD -rps16 exon2 | GTR+G | K3Pu+F+G4 | -20590.1638 | 41194.3659 | 41236.2189 | 1985 | 869 | 1356 | gamma |
| 2 | *accD -psaI, rpl33-rps18* | GTR+G | TIM+F+G4 | -12449.3925 | 24914.8639 | 24958.8902 | 1082 | 532 | 975 | gamma |
| 3 | *atpA - atpF exon2* | GTR+G | K3Pu+F+G4 | -1330.0242 | 2674.8428 | 2695.0761 | 120 | 51 | 57 | gamma |
| 4 | *atpA - trnR-UCU* | GTR+G | K3Pu+F+G4 | -5928.1937 | 11870.5221 | 11903.5128 | 584 | 231 | 383 | gamma |
| 5 | *atpB - rbcL, psbM - trnD-GUC, clpP exon2 - clpP exon3* | GTR+I+G | TVM+F+G4 | -24770.9262 | 49559.9015 | 49615.7386 | 1926 | 950 | 2124 | invgamma |
| 6 | *atpE - trnM-CAU* | GTR+I+G | K3Pu+F+G4 | -2757.836 | 5530.0231 | 5556.2017 | 201 | 99 | 162 | invgamma |
| 7 | *atpF exon1- atpH* | GTR+I+G | K3Pu+F+G4 | -9228.9071 | 18471.921 | 18506.5566 | 759 | 373 | 454 | invgamma |
| 8 | *rpoC1 exon1- rpoC1 exon2, atpF exon1 - atpF exon2, petB exon2 -petD exon1, psbH -psbN* | GTR+I+G | K3Pu+F+I+G4 | -20340.2795 | 40696.6112 | 40743.9573 | 1522 | 744 | 1494 | invgamma |
| 9 | *atpH - atpI, petA - psbJ, trnF-GAA - trnL-UAA exon2, rpl16 exon1- rps3* | GTR+I+G | TVM+F+G4 | -44765.9402 | 89549.9128 | 89609.5359 | 3828 | 1837 | 2553 | invgamma |
| 10 | *psbB - psbT, atpI - rps2, rpl14 -rpl16 exon2* | GTR+I+G | TVM+F+G4 | -7577.7892 | 15173.7829 | 15216.6994 | 597 | 295 | 428 | invgamma |
| 11 | *ccsA - trnL-UAG, ndhE - ndhG_* | GTR+G | TVM+F+G4 | -5343.3942 | 10705.0751 | 10744.9135 | 447 | 200 | 303 | gamma |
| 12 | *ccsA - ndhD, rps16 exon1 -trnQ-UUG* | GTR+I+G | TVM+F+G4 | -13140.7743 | 26299.6699 | 26347.3376 | 1050 | 535 | 638 | invgamma |
| 13 | *psbC - trnS-UGA, cemA - ycf4, psaJ - rpl33, trnD-GUC - trnY-GUA* | GTR+G | TVM+F+G4 | -25013.9991 | 50046.06 | 50099.8184 | 1951 | 952 | 1384 | gamma |
| 14 | *cemA - petA, rpoC2 - rps2* | GTR+G | HKY+F+G4 | -7953.9986 | 15920.1063 | 15947.9299 | 601 | 314 | 282 | gamma |
| 15 | *clpP exon1 -psbB* | GTR+G | K3Pu+F+G4 | -6573.7474 | 13161.6422 | 13194.0014 | 463 | 260 | 372 | gamma |
| 16 | *clpP exon1 -clpP exon2* | GTR+G | K3Pu+F+G4 | -8880.995 | 17776.0895 | 17811.2121 | 705 | 335 | 496 | gamma |
| 17 | *psbA - trnK-UUU exon2, clpP exon3 - rps12 exon1* | GTR+I+G | TIM+F+I+G4 | -8195.6443 | 16409.4333 | 16455.4955 | 645 | 315 | 736 | invgamma |
| 18 | *matK - trnK-UUU exon1* | GTR+I+G | TIM+F+G4 | -8695.3442 | 17406.8147 | 17447.0616 | 673 | 330 | 618 | invgamma |
| 19 | *matK - trnK-UUU exon2, trnS-GGA -ycf3 exon1* | GTR+G | TIM+F+G4 | -9177.7402 | 18371.5965 | 18412.5211 | 744 | 330 | 678 | gamma |
| 20 | *ndhA exon1 -ndhA exon2* | GTR+I+G | K3Pu+F+I+G4 | -18779.6473 | 37575.3571 | 37621.265 | 1394 | 699 | 1139 | invgamma |
| 21 | *ndhA exon2 - ndhI, petB exon1 - psbH* | GTR+G | GTR+F+G4 | -2680.6851 | 5382.1367 | 5418.3411 | 205 | 99 | 142 | gamma |
| 22 | *ndhB exon1 - rps7,* *rrn23 - trnA-UGC exon2, trnL-CAA -ycf2* | GTR+G | TIM+F+G4 | -6250.7582 | 12517.6007 | 12561.0937 | 633 | 214 | 1268 | gamma |
| 23 | *ndhB exon1 -ndhB exon2, rpl2 exon1 -rpl2 exon2* | GTR+I+G | TVM+F+G4 | -5267.733 | 10553.5854 | 10601.3923 | 383 | 149 | 1199 | invgamma |
| 24 | *rpl23 - trnI-CAU, ndhB exon2 - trnL-CAA* | GTR+G | K3Pu+F+G4 | -6611.7807 | 13237.6316 | 13275.2232 | 551 | 233 | 1142 | gamma |
| 25 | *ndhC - trnV-UAC exon2, rpl14 - rps8* | GTR+G | TVM+F+G4 | -12792.5114 | 25603.1233 | 25652.4876 | 1283 | 521 | 748 | gamma |
| 26 | *ndhD - psaC* | GTR+G | HKY+F+G4 | -1617.1687 | 3246.8403 | 3265.2917 | 120 | 70 | 81 | gamma |
| 27 | *psbI - psbK, ndhE - psaC, trnP-UGG - trnW-CCA* | GTR+I+G | TVM+F+I+G4 | -13565.5075 | 27151.1393 | 27205.8643 | 1142 | 525 | 924 | invgamma |
| 28 | *ndhF - rpl32* | GTR+I+G | TVM+F+G4 | -11934.1926 | 23886.4984 | 23934.7907 | 1022 | 475 | 805 | invgamma |
| 29 | *ndhF - trnN-GUU, trnG-UCC exon1 - trnG-UCC exon2* | GTR+I+G | TIM+F+G4 | -14051.1551 | 28118.3616 | 28165.8234 | 1038 | 504 | 1891 | invgamma |
| 30 | *psaJ - trnP-UGG, ndhG - ndhI* | GTR+I+G | K3Pu+F+G4 | -16461.3919 | 32936.8319 | 32977.0651 | 1534 | 692 | 1044 | invgamma |
| 31 | *trnN-GUU - ycf1, ndhH - rps15* | GTR+G | TN+F+G4 | -4485.4279 | 8984.9973 | 9017.6481 | 346 | 157 | 480 | gamma |
| 32 | *ndhJ - ndhK* | GTR+I+G | TPM2u+F+G4 | -2212.6606 | 4439.6983 | 4465.3633 | 221 | 77 | 151 | invgamma |
| 33 | *ndhJ - trnF-GAA* | GTR+G | TVM+F+G4 | -12516.5681 | 25051.2525 | 25099.2966 | 1042 | 492 | 774 | gamma |
| 34 | *trnL-UAA exon1 -trnL-UAA exon2, petB exon1 -petB exon2, petD exon1 – petD exon2* | GTR+I+G | TIM+F+I+G4 | -22513.372 | 45044.8036 | 45098.8789 | 1633 | 855 | 1638 | invgamma |
| 35 | *petD exon2 - rpoA* | GTR+G | K3Pu+F+G4 | -3723.0376 | 7460.3763 | 7487.6565 | 269 | 141 | 175 | gamma |
| 36 | *psbZ - trnS-UGA, petG - petL, trnM-CAU - trnV-UAC exon1_* | GTR+I+G | TVM+F+G4 | -9199.7771 | 18417.7173 | 18462.6875 | 764 | 376 | 522 | invgamma |
| 37 | *petG - trnW-CCA, rps4 - trnS-GGA* | GTR+G | TVM+F+G4 | -5489.689 | 10997.644 | 11038.1691 | 467 | 223 | 345 | gamma |
| 38 | *petL - psbE, rpoB -trnC-GCA* | GTR+I+G | TVM+F+G4 | -34072.4015 | 68162.8456 | 68219.9739 | 2880 | 1383 | 1938 | invgamma |
| 39 | *petN - trnC-GCA* | GTR+G | K3Pu+F+G4 | -14434.5998 | 28883.2597 | 28921.9542 | 1360 | 633 | 861 | gamma |
| 40 | *rpl20 - rps18, petN - psbM, trnE-UUC - trnT-GGU* | GTR+G | TVM+F+G4 | -28970.4872 | 57959.0211 | 58015.3072 | 2443 | 1148 | 1913 | gamma |
| 41 | *psaA - ycf3 exon3* | GTR+I+G | K3Pu+F+G4 | -13577.4958 | 27169.0617 | 27206.6665 | 1049 | 522 | 771 | invgamma |
| 42 | *psaB - rps14, rpl20 - rps12 exon1* | GTR+I+G | K3Pu+F+G4 | -12006.8691 | 24027.8207 | 24064.2757 | 917 | 466 | 617 | invgamma |
| 43 | *psaI - ycf4* | GTR+G | TPM3+F+G4 | -4120.4908 | 8255.2477 | 8283.4118 | 317 | 160 | 167 | gamma |
| 44 | *psbA - trnH-GUG* | GTR+G | TVM+F+G4 | -7123.3671 | 14264.9676 | 14306.6793 | 569 | 263 | 313 | gamma |
| 45 | *psbD - trnT-GGU* | GTR+G | TIM+F+G4 | -21054.9911 | 42126.0399 | 42172.5906 | 1781 | 819 | 1095 | gamma |
| 46 | *psbJ - psbL* | GTR+I+G | HKY+F+G4 | -1500.2086 | 3012.7839 | 3033.2001 | 136 | 58 | 136 | invgamma |
| 47 | *psbK - trnQ-UUG* | GTR+G | K3Pu+F+G4 | -9032.6488 | 18079.3641 | 18117.3249 | 724 | 330 | 1082 | gamma |
| 48 | *trnL-CAA - ycf2, psbZ_- trnG-GCC* | GTR+G | TVM+F+G4 | -32533.4982 | 65085.04 | 65141.9634 | 2911 | 1355 | 1833 | gamma |
| 49 | *rbcL - trnK-UUU exon1* | GTR+I+G | K3Pu+F+G4 | -17582.8646 | 35179.7818 | 35219.4026 | 1569 | 770 | 888 | invgamma |
| 50 | *rpl32 - trnL-UAG* | GTR+G | K3Pu+F+G4 | -12787.75 | 25589.5512 | 25629.3735 | 1157 | 490 | 1122 | gamma |
| 51 | *rpl36 - rps8* | GTR+G | TIM+F+G4 | -8457.1762 | 16930.5184 | 16968.5552 | 657 | 348 | 378 | gamma |
| 52 | *rpoA - rps11* | GTR+G | TIM3+F+G4 | -1094.545 | 2206.5158 | 2226.6939 | 82 | 37 | 51 | gamma |
| 53 | *rpoC1 exon2 -rpoC2, rps14 -trnfM-CAU* | GTR+I+G | TPM3u+F+I+G4 | -4278.6613 | 8573.5844 | 8607.9318 | 346 | 154 | 290 | invgamma |
| 54 | *rps12 exon2 -trnV-GAC* | GTR+G | K3Pu+F+G4 | -10139.1843 | 20292.4173 | 20332.5775 | 949 | 361 | 1578 | gamma |
| 55 | *rps12 exon2 -rps12 exon3* | GTR+G | TPM3u+F+G4 | -1578.6798 | 3171.5369 | 3202.5899 | 117 | 37 | 560 | gamma |
| 56 | *rps15 - ycf1* | GTR+I+G | TVM+F+G4 | -7276.5491 | 14571.2967 | 14614.4882 | 626 | 272 | 464 | invgamma |
| 57 | *rps16 exon1 -rps16 exon2_* | GTR+G | K3Pu+F+G4 | -8416.7321 | 16847.5528 | 16883.5137 | 683 | 316 | 727 | gamma |
| 58 | *rps4 - trnT-UGU* | GTR+G | K3Pu+F+G4 | -4735.7972 | 9485.7557 | 9517.4719 | 365 | 171 | 446 | gamma |
| 59 | *rrn4.5 - rrn5, rrn16 - trnV-GAC* | GTR+G | K3Pu+F+G4 | -2310.4559 | 4635.1125 | 4665.2819 | 239 | 80 | 414 | gamma |
| 60 | *rrn16 - trnI-GAU exon1* | GTR+I+G | K3P+I+G4 | -1881.0594 | 3772.2995 | 3791.2341 | 150 | 57 | 218 | invgamma |
| 61 | *rrn23 - rrn4.5, trnA-UGC exon1 - trnA-UGC exon2, trnI-GAU exon1 - trnI-GAU exon2* | GTR+I+G | K3P+G4 | -6484.4278 | 12976.8755 | 12999.2911 | 460 | 173 | 1638 | invgamma |
| 62 | *rrn5 - trnR-ACG* | GTR+G | K3Pu+F+G4 | -1505.8569 | 3026.0376 | 3052.7989 | 175 | 52 | 256 | gamma |
| 63 | *trnG-UCC exon2 - trnR-UCU* | GTR+G | TVM+F+G4 | -5359.725 | 10737.8085 | 10775.5949 | 433 | 222 | 165 | gamma |
| 64 | *trnN-GUU - trnR-ACG* | GTR+G | GTR+F+G4 | -4838.128 | 9696.5275 | 9743.3611 | 437 | 198 | 492 | gamma |
| 65 | *trnV-UAC exon1 - trnV-UAC exon2* | GTR+I+G | TVM+F+G4 | -5256.1655 | 10530.5685 | 10572.1251 | 428 | 189 | 441 | invgamma |
| 66 | *ycf3 exon1 -ycf3 exon2* | GTR+I+G | HKY+F+I+G4 | -6894.1007 | 13802.3195 | 13836.2481 | 531 | 251 | 540 | invgamma |
| 67 | *ycf3 exon2 -ycf3 exon3* | GTR+I+G | K3Pu+F+G4 | -8124.3349 | 16262.7626 | 16298.3931 | 651 | 297 | 709 | invgamma |

AICc: Corrected AIC scores, BIC: Bayesian information criterion scores, Unique: Number of unique site patterns, Infor: Number of parsimony-informative sites, Invar: Number of invariant sites

**Table S3C: Details of CP data matrix**

Scheme lnL: -1173990.4572753906

Scheme AICc: 2350393.37506

Number of params: 1198

Number of sites: 175726

Number of subsets: 104

| **Subset** | **Partition names** | **Best Model** | **Best-fit Model** | **LogL** | **AICc** | **BIC** | **Unique** | **Infor** | **Invar** | **rates** |
| --- | --- | --- | --- | --- | --- | --- | --- | --- | --- | --- |
| 1 | *trnG-UCC exon1 - trnS-GCU,*  *psbI -trnS-GCU,*  *accD - rps16 exon2* | GTR+G | K3Pu+F+G4 | -20602.9753 | 41219.9888 | 41261.8418 | 1985 | 869 | 1356 | gamma |
| 2 | *rpl33 - rps18, rpoC1 exon1 -rpoC1 exon2* | GTR+G | TIM+F+G4 | -12465.5349 | 24947.1488 | 24991.1751 | 1082 | 532 | 975 | gamma |
| 3 | *rps15, rpl20, accD,*  *trnV-UAC exon1 - trnV-UAC exon2* | GTR+I+G | TVM+F+I+G4 | -26108.3117 | 52236.6825 | 52298.8731 | 1636 | 940 | 2198 | invgamma |
| 4 | *atpA - atpF exon2* | GTR+G | K3Pu+F+G4 | -1329.7565 | 2674.3073 | 2694.5406 | 120 | 51 | 57 | gamma |
| 5 | *atpA - trnR-UCU* | GTR+G | K3Pu+F+G4 | -5931.0134 | 11876.1617 | 11909.1524 | 584 | 231 | 383 | gamma |
| 6 | *atpB, atpA, psbT* | GTR+I+G | TVM+F+I+G4 | -16602.9597 | 33225.9892 | 33286.5185 | 747 | 538 | 2305 | invgamma |
| 7 | *atpB - rbcL,*  *clpP exon2 -clpP exon3,*  *psbM - trnD-GUC* | GTR+I+G | TVM+F+G4 | -24765.7416 | 49549.5323 | 49605.3694 | 1926 | 950 | 2124 | invgamma |
| 8 | *atpE - trnM-CAU* | GTR+I+G | K3Pu+F+G4 | -2755.1135 | 5524.5781 | 5550.7567 | 201 | 99 | 162 | invgamma |
| 9 | *rps16, rps4, atpE* | GTR+G | TVM+F+G4 | -7565.8645 | 15149.8631 | 15196.6198 | 477 | 268 | 871 | gamma |
| 10 | *atpF exon1 - atpH* | GTR+G | K3Pu+F+G4 | -9233.9549 | 18482.0165 | 18516.6521 | 759 | 373 | 454 | gamma |
| 11 | *rpoC1 exon1 -rpoC1 exon2,*  *atpF exon1 - atpF exon2,*  *petB exon2 - petD exon1* | GTR+I+G | K3Pu+F+I+G4 | -19272.4889 | 38561.0331 | 38607.93 | 1454 | 711 | 1403 | invgamma |
| 12 | *atpF, rps11* | GTR+I+G | K3Pu+F+I+G4 | -6850.3839 | 13716.9012 | 13756.712 | 385 | 248 | 671 | invgamma |
| 13 | *atpH - atpI,*  *petA - psbJ,*  *trnF-GAA - trnL-UAA exon2,*  *rpl16 exon1 - rps3* | GTR+I+G | TVM+F+G4 | -44788.833 | 89595.6982 | 89655.3213 | 3828 | 1837 | 2553 | invgamma |
| 14 | *rbcL, atpH, petB* | GTR+I+G | TVM+F+I+G4 | -12058.3124 | 24136.7196 | 24194.1653 | 501 | 391 | 1795 | invgamma |
| 15 | *psbB - psbT,*  *rpl14 -rpl16 exon2,*  *atpI - rps2* | GTR+I+G | TVM+F+G4 | -7576.5549 | 15171.3144 | 15214.2308 | 597 | 295 | 428 | invgamma |
| 16 | *ndhC, ndhE, atpI* | GTR+I+G | TVM+F+I+G4 | -7460.6836 | 14941.5242 | 14993.9019 | 401 | 268 | 1006 | invgamma |
| 17 | *ndhE - ndhG,*  *rps4 - trnT-UGU,*  *ccsA -trnL-UAG* | GTR+I+G | TVM+F+G4 | -10081.1988 | 20180.5329 | 20227.2014 | 791 | 371 | 749 | invgamma |
| 18 | *ccsA - ndhD,*  *rps16 exon1 -trnQ-UUG* | GTR+I+G | TVM+F+G4 | -13158.2607 | 26334.6426 | 26382.3103 | 1050 | 535 | 638 | invgamma |
| 19 | *ccsA* | GTR+I+G | TVM+F+I+G4 | -8534.0252 | 17088.2609 | 17137.6728 | 504 | 313 | 575 | invgamma |
| 20 | *clpP exon1 - psbB,*  *psbC - trnS-UGA,*  *trnD-GUC -trnY-GUA, cemA - ycf4,*  *psaJ - rpl33* | GTR+G | TVM+F+G4 | -31605.3249 | 63228.6987 | 63284.5702 | 2374 | 1212 | 1756 | gamma |
| 21 | *cemA - petA,*  *rpoC2 - rps2* | GTR+G | HKY+F+G4 | -7955.5843 | 15923.2777 | 15951.1013 | 601 | 314 | 282 | gamma |
| 22 | *cemA* | GTR+G | TVM+F+G4 | -5190.1031 | 10398.4619 | 10439.3442 | 296 | 190 | 409 | gamma |
| 23 | *clpP exon1 - clpP exon2* | GTR+G | K3Pu+F+G4 | -8868.3444 | 17750.7885 | 17785.911 | 705 | 335 | 496 | gamma |
| 24 | *clpP exon3 -rps12 exon1, psbA - trnK-UUU exon2* | GTR+I+G | TIM+F+I+G4 | -8195.9427 | 16410.03 | 16456.0921 | 645 | 315 | 736 | invgamma |
| 25 | *clpP* | GTR+I+G | TPM3u+F+G4 | -5224.9873 | 10464.1648 | 10494.7181 | 287 | 182 | 268 | invgamma |
| 26 | *matK 3 - trnK-UUU exon1* | GTR+I+G | TIM+F+G4 | -8697.8303 | 17411.787 | 17452.0338 | 673 | 330 | 618 | invgamma |
| 27 | *matK - trnK-UUU exon2* | GTR+I+G | GTR+F+G4 | -5435.0427 | 10890.3536 | 10937.3116 | 455 | 190 | 492 | invgamma |
| 28 | *matK* | GTR+I+G | TVM+F+G4 | -14701.972 | 29422.0564 | 29470.4054 | 870 | 598 | 715 | invgamma |
| 29 | *ndhA exon1 -ndhA exon2* | GTR+I+G | K3Pu+F+I+G4 | -18783.372 | 37582.8065 | 37628.7144 | 1394 | 699 | 1139 | invgamma |
| 30 | *petB exon1 -psbH,*  *ndhA exon2 - ndhI* | GTR+G | GTR+F+G4 | -2681.6761 | 5384.1187 | 5420.3231 | 205 | 99 | 142 | gamma |
| 31 | *ndhA, psbK, ndhD* | GTR+I+G | GTR+F+I+G4 | -19032.511 | 38087.1141 | 38152.6469 | 910 | 669 | 1852 | invgamma |
| 32 | *ndhB exon1 -rps7,*  *rrn23 - trnA-UGC exon2, trnL-CAA - ycf2* | GTR+G | TIM+F+G4 | -6247.2915 | 12510.6673 | 12554.1603 | 633 | 214 | 1268 | gamma |
| 33 | *ndhB exon1 -ndhB exon2, rpl23,*  *rpl2 exon1 - rpl2 exon2* | GTR+I+G | TVM+F+G4 | -6233.8438 | 12485.7872 | 12535.2371 | 446 | 172 | 1446 | invgamma |
| 34 | *rpl23 - trnI-CAU,*  *ndhB exon2 - trnL-CAA* | GTR+G | K3Pu+F+G4 | -6603.0403 | 13220.1507 | 13257.7423 | 551 | 233 | 1142 | gamma |
| 35 | *ndhB* | GTR+I+G | TVM+F+G4 | -4236.521 | 8491.1645 | 8538.734 | 185 | 103 | 1260 | invgamma |
| 36 | *ndhC - trnV-UAC exon2, rpl14 - rps8* | GTR+G | TVM+F+G4 | -12803.7287 | 25625.5579 | 25674.9223 | 1283 | 521 | 748 | gamma |
| 37 | *ndhD - psaC* | GTR+G | HKY+F+G4 | -1617.6192 | 3247.7414 | 3266.1927 | 120 | 70 | 81 | gamma |
| 38 | *trnP-UGG -trnW-CCA, ndhE - psaC* | GTR+I+G | TVM+F+G4 | -7197.8464 | 14413.8748 | 14457.8536 | 660 | 269 | 531 | invgamma |
| 39 | *ndhF - rpl32* | GTR+I+G | TVM+F+G4 | -11940.6358 | 23899.3847 | 23947.677 | 1022 | 475 | 805 | invgamma |
| 40 | *trnG-UCC exon1 - trnG-UCC exon2,*  *ndhF - trnN-GUU* | GTR+I+G | TIM+F+G4 | -14045.9349 | 28107.9213 | 28155.3831 | 1038 | 504 | 1891 | invgamma |
| 41 | *ndhF* | GTR+I+G | TVM+F+I+G4 | -20816.0295 | 41652.1527 | 41709.7275 | 999 | 748 | 1271 | invgamma |
| 42 | *ndhG - ndhI,*  *psaJ - trnP-UGG* | GTR+I+G | K3Pu+F+G4 | -16469.8472 | 32953.7426 | 32993.9758 | 1534 | 692 | 1044 | invgamma |
| 43 | *ndhG, psaI* | GTR+I+G | TVM+F+G4 | -4137.1995 | 8292.682 | 8332.6362 | 262 | 163 | 389 | invgamma |
| 44 | *ndhH - rps15,*  *trnN-GUU - ycf1* | GTR+G | TN+F+G4 | -4479.9747 | 8974.0907 | 9006.7416 | 346 | 157 | 480 | gamma |
| 45 | *ndhH* | GTR+I+G | TPM3u+F+I+G4 | -6933.2435 | 13882.6091 | 13923.1271 | 346 | 240 | 819 | invgamma |
| 46 | *ndhI, ndhK* | GTR+I+G | TPM2u+F+I+G4 | -7811.5935 | 15639.3017 | 15680.3297 | 458 | 273 | 827 | invgamma |
| 47 | *ndhJ - ndhK* | GTR+I+G | TPM2u+F+G4 | -2215.1008 | 4444.5788 | 4470.2439 | 221 | 77 | 151 | invgamma |
| 48 | *ndhJ - trnF-GAA* | GTR+G | TVM+F+G4 | -12516.9987 | 25052.1136 | 25100.1578 | 1042 | 492 | 774 | gamma |
| 49 | *rpl14, ndhJ, rpoC1, rps2* | GTR+I+G | TVM+F+I+G4 | -20681.8844 | 41383.8287 | 41445.8781 | 1018 | 744 | 2463 | invgamma |
| 50 | *rps14, psbH, petA, rpoB* | GTR+I+G | TVM+F+I+G4 | -27335.8836 | 54691.8136 | 54756.443 | 1232 | 979 | 3172 | invgamma |
| 51 | *trnL-UAA exon1 - trnL-UAA exon2,*  *petB exon1 -petB exon2, petD exon1 -petD exon2* | GTR+I+G | TIM+F+I+G4 | -22516.1086 | 45050.2769 | 45104.3522 | 1633 | 855 | 1638 | invgamma |
| 52 | *petD exon2 - rpoA_* | GTR+G | K3Pu+F+G4 | -3725.428 | 7465.1571 | 7492.4372 | 269 | 141 | 175 | gamma |
| 53 | *psbB, petD* | GTR+I+G | GTR+F+I+G4 | -9824.7219 | 19671.5754 | 19733.1578 | 430 | 318 | 1528 | invgamma |
| 54 | *psbZ - trnS-UGA,*  *trnM-CAU -trnV-UAC exon1,*  *petG - petL* | GTR+I+G | TVM+F+G4 | -9207.9211 | 18434.0053 | 18478.9754 | 764 | 376 | 522 | invgamma |
| 55 | *rps4 - trnS-GGA,*  *petG - trnW-CCA* | GTR+G | TVM+F+G4 | -5492.1963 | 11002.6585 | 11043.1837 | 467 | 223 | 345 | gamma |
| 56 | *psbE, psbF, petN, psaC, petG, psbJ* | GTR+I+G | TVM+F+I+G4 | -3655.7903 | 7331.8161 | 7380.0924 | 178 | 112 | 756 | invgamma |
| 57 | *petL - psbE* | GTR+I+G | TVM+F+G4 | -17205.0231 | 34428.1284 | 34479.3162 | 1516 | 705 | 999 | invgamma |
| 58 | *petN - trnC-GCA* | GTR+G | K3Pu+F+G4 | -14436.2092 | 28886.4784 | 28925.173 | 1360 | 633 | 861 | gamma |
| 59 | *rpoB - trnC-GCA,*  *petN - psbM,*  *trnE-UUC - trnT-GGU* | GTR+G | GTR+F+G4 | -42430.7797 | 84881.5993 | 84947.7261 | 3544 | 1675 | 2691 | gamma |
| 60 | *psaA -ycf3 exon3* | GTR+I+G | K3Pu+F+G4 | -13578.7126 | 27171.4952 | 27209.1 | 1049 | 522 | 771 | invgamma |
| 61 | *psaA, psaB* | GTR+I+G | TVM+F+I+G4 | -20917.7295 | 41855.5084 | 41919.4835 | 854 | 685 | 3399 | invgamma |
| 62 | *psaB - rps14,*  *rpl20 - rps12 exon1* | GTR+I+G | K3Pu+F+G4 | -12012.9161 | 24039.9148 | 24076.3698 | 917 | 466 | 617 | invgamma |
| 63 | *psaI - ycf4* | GTR+G | TPM3+F+G4 | -4114.0104 | 8242.2868 | 8270.451 | 317 | 160 | 167 | gamma |
| 64 | *psaJ, psbI* | GTR+G | TPM2u+F+G4 | -1389.5434 | 2793.5573 | 2817.624 | 88 | 52 | 167 | gamma |
| 65 | *psbA - trnH-GUG* | GTR+G | TVM+F+G4 | -7109.7395 | 14237.7124 | 14279.4241 | 569 | 263 | 313 | gamma |
| 66 | *psbL, psbA* | GTR+I+G | HKY+F+I+G4 | -4633.0233 | 9280.1422 | 9315.5535 | 203 | 130 | 957 | invgamma |
| 67 | *psbC, psbD* | GTR+I+G | K3Pu+F+I+G4 | -10278.8191 | 20573.6964 | 20620.1792 | 437 | 313 | 1975 | invgamma |
| 68 | *psbD - trnT-GGU* | GTR+G | TIM+F+G4 | -21064.6768 | 42145.4114 | 42191.962 | 1781 | 819 | 1095 | gamma |
| 69 | *ycf3 exon2 - ycf3 exon3, psbH - psbN* | GTR+I+G | K3Pu+F+G4 | -9195.15 | 18404.3824 | 18440.8375 | 728 | 330 | 800 | invgamma |
| 70 | *psbI - psbK, rpl20 - rps18,*  *trnS-GGA - ycf3 exon1* | GTR+I+G | TVM+F+G4 | -13501.8253 | 27021.7654 | 27069.9316 | 1094 | 547 | 740 | invgamma |
| 71 | *psbJ – psbL* | GTR+I+G | HKY+F+G4 | -1500.5627 | 3013.4922 | 3033.9084 | 136 | 58 | 136 | invgamma |
| 72 | *psbK - trnQ-UUG* | GTR+G | K3Pu+F+G4 | -9037.7264 | 18089.5194 | 18127.4802 | 724 | 330 | 1082 | gamma |
| 73 | *psbZ, psbM* | GTR+G | K3Pu+F+G4 | -1295.2231 | 2604.8338 | 2630.3024 | 84 | 40 | 217 | gamma |
| 74 | *rps12, psbN, ycf3* | GTR+I+G | K3Pu+F+I+G4 | -4104.3952 | 8224.9324 | 8264.2343 | 210 | 127 | 804 | invgamma |
| 75 | *trnL-UAA exon1 - trnT-UGU,*  *psbZ - trnG-GCC* | GTR+G | TVM+F+G4 | -32548.6123 | 65115.2682 | 65172.1916 | 2911 | 1355 | 1833 | gamma |
| 76 | *rbcL - trnK-UUU exon1* | GTR+I+G | K3Pu+F+G4 | -17593.4273 | 35200.9071 | 35240.5279 | 1569 | 770 | 888 | invgamma |
| 77 | *rpl16, rpl36, rps3* | GTR+I+G | TVM+F+I+G4 | -8178.5589 | 16377.3032 | 16427.9935 | 428 | 271 | 743 | invgamma |
| 78 | *rpl2, rps7, rrn4.5* | GTR+G | K3Pu+F+G4 | -4501.1575 | 9016.3952 | 9053.0395 | 224 | 116 | 1152 | gamma |
| 79 | *rpl32 - trnL-UAG* | GTR+G | K3Pu+F+G4 | -12792.577 | 25599.2051 | 25639.0275 | 1157 | 490 | 1122 | gamma |
| 80 | *rpl32* | GTR+I+G | GTR+F+I+G4 | -1586.2167 | 3196.0238 | 3229.4331 | 97 | 58 | 94 | invgamma |
| 81 | *rpoA, rps8, rpl33* | GTR+I+G | TVM+F+G4 | -11825.2384 | 23668.5848 | 23717.2997 | 682 | 432 | 948 | invgamma |
| 82 | *rpl36 - rps8* | GTR+G | TIM+F+G4 | -8458.2919 | 16932.7499 | 16970.7867 | 657 | 348 | 378 | gamma |
| 83 | *rpoA - rps11* | GTR+G | TIM3+F+G4 | -1094.1917 | 2205.8092 | 2225.9873 | 82 | 37 | 51 | gamma |
| 84 | *rpoC1 exon2 - rpoC2* | GTR+I+G | TPM3u+F+I+G4 | -2225.9146 | 4468.3788 | 4496.6462 | 190 | 82 | 131 | invgamma |
| 85 | *rpoC2* | GTR+I+G | GTR+F+I+G4 | -33274.8087 | 66571.6777 | 66641.8828 | 1750 | 1294 | 2353 | invgamma |
| 86 | *rps12 exon2 - trnV-GAC* | GTR+G | K3Pu+F+G4 | -10141.1894 | 20296.4276 | 20336.5879 | 949 | 361 | 1578 | gamma |
| 87 | *rps12 exon2 -rps12 exon3* | GTR+G | TPM3u+F+G4 | -1577.8761 | 3169.9294 | 3200.9825 | 117 | 37 | 560 | gamma |
| 88 | *rps14 - trnfM-CAU* | GTR+I+G | TIM3+F+G4 | -2043.3033 | 4103.1227 | 4131.9103 | 165 | 72 | 159 | invgamma |
| 89 | *rps15 - ycf1* | GTR+I+G | TVM+F+G4 | -7271.5 | 14561.1985 | 14604.39 | 626 | 272 | 464 | invgamma |
| 90 | *rps16 exon1 - rps16 exon2* | GTR+G | K3Pu+F+G4 | -8417.6732 | 16849.4348 | 16885.3958 | 683 | 316 | 727 | gamma |
| 91 | *rps18* | GTR+I+G | HKY+F+I+G4 | -2789.6007 | 5593.4806 | 5621.2973 | 193 | 87 | 232 | invgamma |
| 92 | *rps19* | GTR+I+G | TIM2+F+G4 | -1589.4595 | 3195.4407 | 3224.1389 | 93 | 54 | 196 | invgamma |
| 93 | *rrn16 - trnV-GAC* | GTR+G | K3Pu+F+G4 | -2308.8118 | 4631.8243 | 4661.9938 | 239 | 80 | 414 |  |
| 94 | *rrn4.5 - rrn5* | GTR+I+G | K3P+I+G4 | -1879.3501 | 3768.8809 | 3787.8154 | 150 | 57 | 218 | invgamma |
| 95 | *rrn16, rrn5* | GTR+I+G | HKY+F+I | -3154.3014 | 6320.655 | 6352.929 | 83 | 34 | 1528 | invgamma |
| 96 | *rrn23 - rrn4.5* | GTR+I+G | K3P+G4 | -6481.0012 | 12970.0224 | 12992.438 | 460 | 173 | 1638 | invgamma |
| 97 | *rrn23* | GTR+I+G | TIM+F+I+G4 | -6761.2827 | 13540.6287 | 13594.1832 | 233 | 124 | 2597 | invgamma |
| 98 | *rrn5 - trnR-ACG* | GTR+G | K3Pu+F+G4 | -1505.2508 | 3024.8253 | 3051.5866 | 175 | 52 | 256 | gamma |
| 99 | *trnG-UCC exon2 - trnR-UCU* | GTR+G | TVM+F+G4 | -5362.0816 | 10742.5218 | 10780.3081 | 433 | 222 | 165 | gamma |
| 100 | *trnN-GUU - trnR-ACG* | GTR+G | GTR+F+G4 | -4839.4557 | 9699.183 | 9746.0166 | 437 | 198 | 492 | gamma |
| 101 | *ycf1* | GTR+I+G | TVM+F+I+G4 | -75983.8268 | 151987.6855 | 152056.0826 | 3993 | 2704 | 2792 | invgamma |
| 102 | *ycf2* | GTR+I+G | TVM+F+G4 | -31932.7252 | 63883.475 | 63945.5505 | 1660 | 1058 | 5128 | invgamma |
| 103 | *ycf3 exon1- ycf3 exon2* | GTR+I+G | HKY+F+I+G4 | -6895.2866 | 13804.6912 | 13838.6199 | 531 | 251 | 540 | invgamma |
| 104 | *ycf4* | GTR+I+G | TVM+F+I+G4 | -9452.8078 | 18925.9405 | 18970.9534 | 510 | 365 | 200 | invgamma |

AICc: Corrected AIC scores, BIC: Bayesian information criterion scores, Unique: Number of unique site patterns, Infor: Number of parsimony-informative sites, Invar: Number of invariant sites
