# Supplementary material for: New Insights Into the Plastome Evolution of the Millettioid/Phaseoloid Clade (Papilionoideae, Leguminosae)
Source: Front Plant Sci. 2020 Mar 10;11:151. doi: 10.3389/fpls.2020.00151 (PMC7076112; doi:10.3389/fpls.2020.00151)
Supplement: Supplementary file 1 [file Figure S2a IR expansion and contraction.PDF › Supplementary/Figure S2a IR expansion and contraction.PDF]

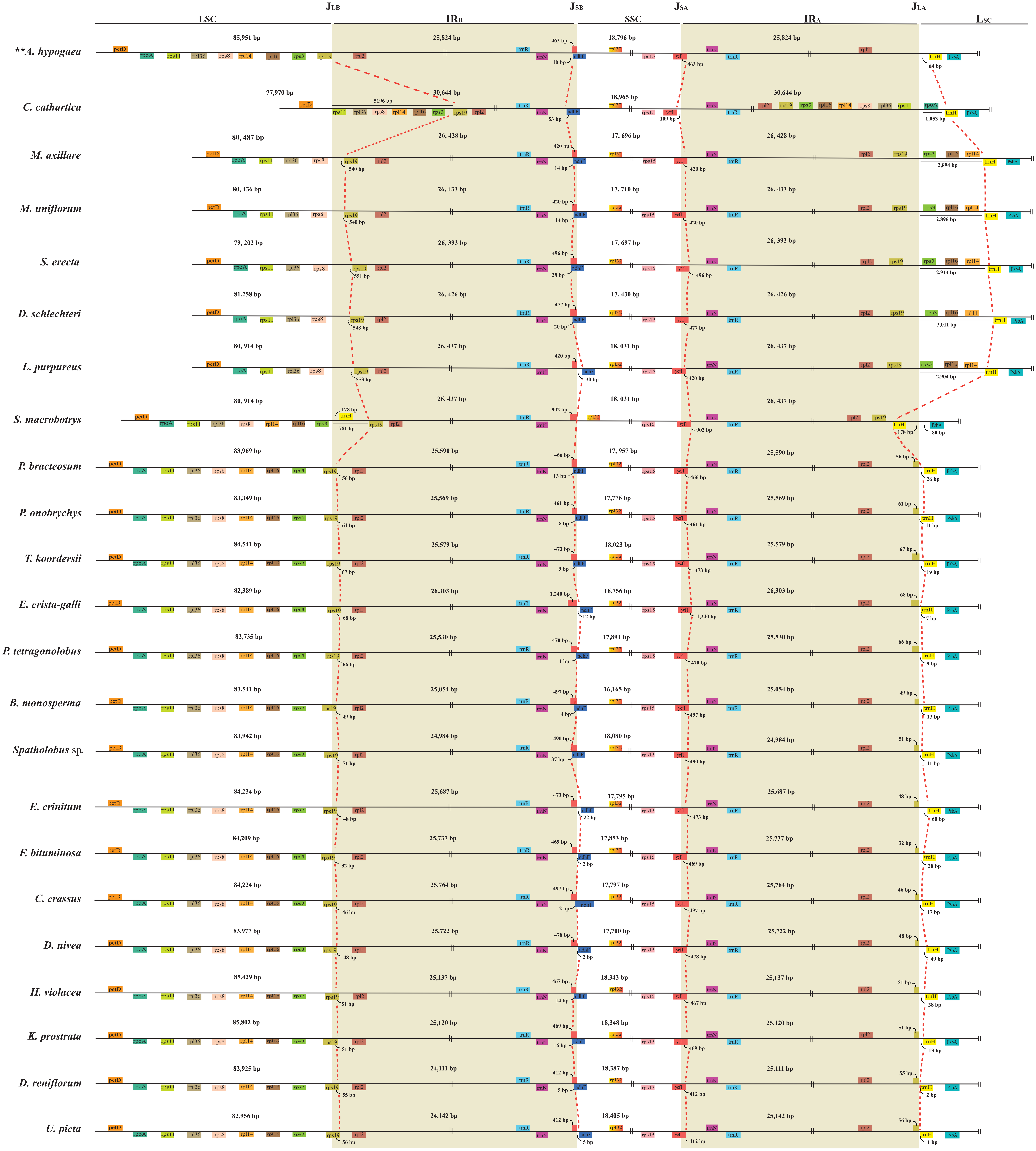

JLB
JSB
JSA
JLA
LSC
IRB
SSC
IRA
LSC
**A. hypogaea
85,951 bp
25,824 bp
18,796 bp
25,824 bp
463 bp
10 bp
463 bp
64 bp
C. cathartica
77,970 bp
5196 bp
30,644 bp
18,965 bp
30,644 bp
53 bp
109 bp
1,053 bp
M. axillare
80, 487 bp
26, 428 bp
17, 696 bp
26, 428 bp
420 bp
540 bp
14 bp
420 bp
2,894 bp
M. uniflorum
80, 436 bp
26, 433 bp
17, 710 bp
26, 433 bp
420 bp
540 bp
14 bp
420 bp
2,896 bp
S. erecta
79, 202 bp
26, 393 bp
17, 697 bp
26, 393 bp
496 bp
551 bp
28 bp
496 bp
2,914 bp
D. schlechteri
81,258 bp
26, 426 bp
17, 430 bp
26, 426 bp
477 bp
548 bp
20 bp
477 bp
3,011 bp
L. purpureus
80, 914 bp
26, 437 bp
18, 031 bp
26, 437 bp
420 bp
553 bp
30 bp
420 bp
2,904 bp
S. macrobotrys
80, 914 bp
26, 437 bp
18, 031 bp
26, 437 bp
178 bp
781 bp
902 bp
902 bp
178 bp
80 bp
P. bracteosum
83,969 bp
25,590 bp
17, 957 bp
25,590 bp
56 bp
466 bp
13 bp
466 bp
56 bp
26 bp
P. onobrychys
83,349 bp
25,569 bp
17,776 bp
25,569 bp
61 bp
461 bp
8 bp
461 bp
61 bp
11 bp
T. koordersii
84,541 bp
25,579 bp
18,023 bp
25,579 bp
67 bp
473 bp
9 bp
473 bp
67 bp
19 bp
E. crista-galli
82,389 bp
26,303 bp
16,756 bp
26,303 bp
68 bp
1,240 bp
12 bp
1,240 bp
68 bp
7 bp
P. tetragonolobus
82,735 bp
25,530 bp
17,891 bp
25,530 bp
66 bp
470 bp
1 bp
470 bp
66 bp
9 bp
B. monosperma
83,541 bp
25,054 bp
16,165 bp
25,054 bp
49 bp
497 bp
4 bp
497 bp
49 bp
13 bp
Spatholobus sp.
83,942 bp
24,984 bp
18,080 bp
24,984 bp
51 bp
490 bp
37 bp
490 bp
51 bp
11 bp
E. crinitum
84,234 bp
25,687 bp
17,795 bp
25,687 bp
48 bp
473 bp
22 bp
473 bp
48 bp
60 bp
F. bituminosa
84,209 bp
25,737 bp
17,853 bp
25,737 bp
32 bp
469 bp
2 bp
469 bp
32 bp
28 bp
C. crassus
84,224 bp
25,764 bp
17,797 bp
25,764 bp
46 bp
497 bp
2 bp
497 bp
46 bp
17 bp
D. nivea
83,977 bp
25,722 bp
17,700 bp
25,722 bp
48 bp
478 bp
2 bp
478 bp
48 bp
49 bp
H. violacea
85,429 bp
25,137 bp
18,343 bp
25,137 bp
51 bp
467 bp
14 bp
467 bp
51 bp
38 bp
K. prostrata
85,802 bp
25,120 bp
18,348 bp
25,120 bp
51 bp
469 bp
16 bp
469 bp
51 bp
13 bp
D. reniflorum
82,925 bp
24,111 bp
18,387 bp
25,111 bp
55 bp
412 bp
5 bp
412 bp
55 bp
2 bp
U. picta
82,956 bp
24,142 bp
18,405 bp
25,142 bp
56 bp
412 bp
5 bp
412 bp
56 bp
1 bp
petD
rpoA
rps11
rpl36
rps8
rpl14
rpl16
rps3
rps19
rpl2
trnR
trnN
ndhF
rpl32
rps15
ycf1
trnH
PsbA
