# Supplementary material for: New Insights Into the Plastome Evolution of the Millettioid/Phaseoloid Clade (Papilionoideae, Leguminosae)
Source: Front Plant Sci. 2020 Mar 10;11:151. doi: 10.3389/fpls.2020.00151 (PMC7076112; doi:10.3389/fpls.2020.00151)
Supplement: Supplementary file 1 [file Figure S2b IR expansion and contraction.PDF › Supplementary/Figure S2b IR expansion and contraction.PDF]

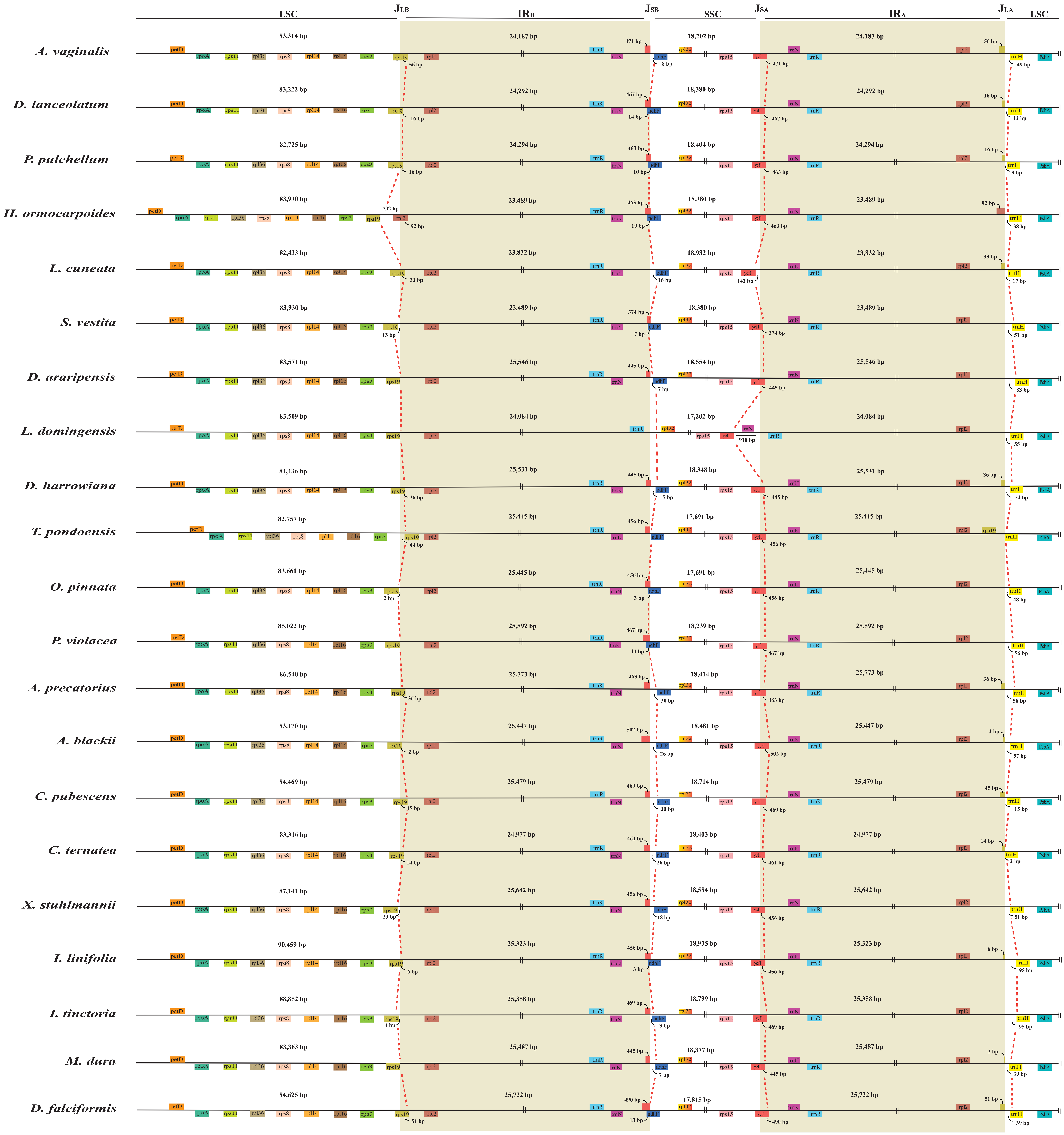
LSC
JLB
IRB
JSB
SSC
JSA
IRA
JLA
LSC
A. vaginalis
83,314 bp
24,187 bp
18,202 bp
24,187 bp
D. lanceolatum
83,222 bp
24,292 bp
18,380 bp
24,292 bp
P. pulchellum
82,725 bp
24,294 bp
18,404 bp
24,294 bp
H. ormocarpoides
83,930 bp
23,489 bp
18,380 bp
23,489 bp
L. cuneata
82,433 bp
23,832 bp
18,932 bp
23,832 bp
S. vestita
83,930 bp
23,489 bp
18,380 bp
23,489 bp
D. araripensis
83,571 bp
25,546 bp
18,554 bp
25,546 bp
L. domingensis
83,509 bp
24,084 bp
17,202 bp
24,084 bp
D. harrowiana
84,436 bp
25,531 bp
18,348 bp
25,531 bp
T. pondoensis
82,757 bp
25,445 bp
17,691 bp
25,445 bp
O. pinnata
83,661 bp
25,445 bp
17,691 bp
25,445 bp
P. violacea
85,022 bp
25,592 bp
18,239 bp
25,592 bp
A. precatorius
86,540 bp
25,773 bp
18,414 bp
25,773 bp
A. blackii
83,170 bp
25,447 bp
18,481 bp
25,447 bp
C. pubescens
84,469 bp
25,479 bp
18,714 bp
25,479 bp
C. ternatea
83,316 bp
24,977 bp
18,403 bp
24,977 bp
X. stuhlmannii
87,141 bp
25,642 bp
18,584 bp
25,642 bp
I. linifolia
90,459 bp
25,323 bp
18,935 bp
25,323 bp
I. tinctoria
88,852 bp
25,358 bp
18,799 bp
25,358 bp
M. dura
83,363 bp
25,487 bp
18,377 bp
25,487 bp
D. falciformis
84,625 bp
25,722 bp
17,815 bp
25,722 bp
